# Dehydropolymerization of Amine−Boranes using Bis(imino)pyridine Rhodium Pre‐Catalysis: σ‐Amine−Borane Complexes, Nanoparticles, and Low Residual‐Metal BN−Polymers that can be Chemically Repurposed

**DOI:** 10.1002/chem.202302110

**Published:** 2023-09-21

**Authors:** Mathew J. Cross, Claire N. Brodie, Dana G. Crivoi, Joe C. Goodall, David E. Ryan, Antonio J. Martínez‐Martínez, Alice Johnson, Andrew S. Weller

**Affiliations:** ^1^ Department of Chemistry University of York York YO10 5DD UK; ^2^ Department of Chemistry University of Oxford Oxford OX1 3TA UK; ^3^ Supramolecular Organometallic and Main Group Chemistry Laboratory CIQSO-Center for Research in Sustainable Chemistry and Department of Chemistry University of Huelva Campus El Carmen 21007 Huelva Spain; ^4^ Department of Biosciences and Chemistry Sheffield Hallam University Howard St Sheffield S1 1WB UK

**Keywords:** amine−borane, dehydropolymerization, mechanism, nanoparticle, rhodium

## Abstract

The sigma amine−borane complexes [Rh(**L1**)(η^2^:η^2^‐H_3_B⋅NRH_2_)][OTf] (**L1**=2,6‐bis‐[1‐(2,6‐diisopropylphenylimino)ethyl]pyridine, R=Me, Et, ^*n*^Pr) are described, alongside [Rh(**L1**)(NMeH_2_)][OTf]. Using R=Me as a pre‐catalyst (1 mol %) the dehydropolymerization of H_3_B ⋅ NMeH_2_ gives [H_2_BNMeH]_n_ selectively. Added NMeH_2_, or the direct use of [Rh(**L1**)(NMeH_2_)][OTf], is required for initiation of catalysis, which is suggested to operate through the formation of a neutral hydride complex, Rh(**L1**)H. The formation of small (1–5 nm) nanoparticles is observed at the end of catalysis, but studies are ambiguous as to whether the catalysis is solely nanoparticle promoted or if there is a molecular homogeneous component. [Rh(**L1**)(NMeH_2_)][OTf] is shown to operate at 0.025 mol % loadings on a 2 g scale of H_3_B ⋅ NMeH_2_ to give polyaminoborane [H_2_BNMeH]_n_ [*M*_n_=30,900 g/mol, Ð=1.8] that can be purified to a low residual [Rh] (6 μg/g). Addition of Na[N(SiMe_3_)_2_] to [H_2_BNMeH]_n_ results in selective depolymerization to form the *eee*‐isomer of *N,N,N*‐trimethylcyclotriborazane [H_2_BNMeH]_3_: the chemical repurposing of a main‐group polymer.

## Graphical abstract

The dehydropolymerization of H_3_B ⋅ NMeH_2_ using bis(imino)pyridine rhodium pre‐catalysis reveals the σ‐amine−borane complexes, nanoparticle formation, and polyaminoboranes with low residual metal content that can be chemically repurposed to *N,N,N*‐trimethylcyclotriborazane.

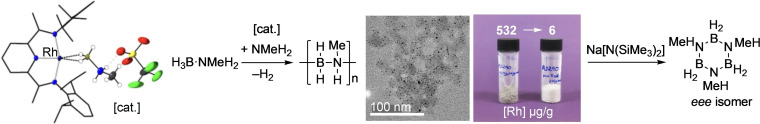

## Introduction

**Scheme 1:**
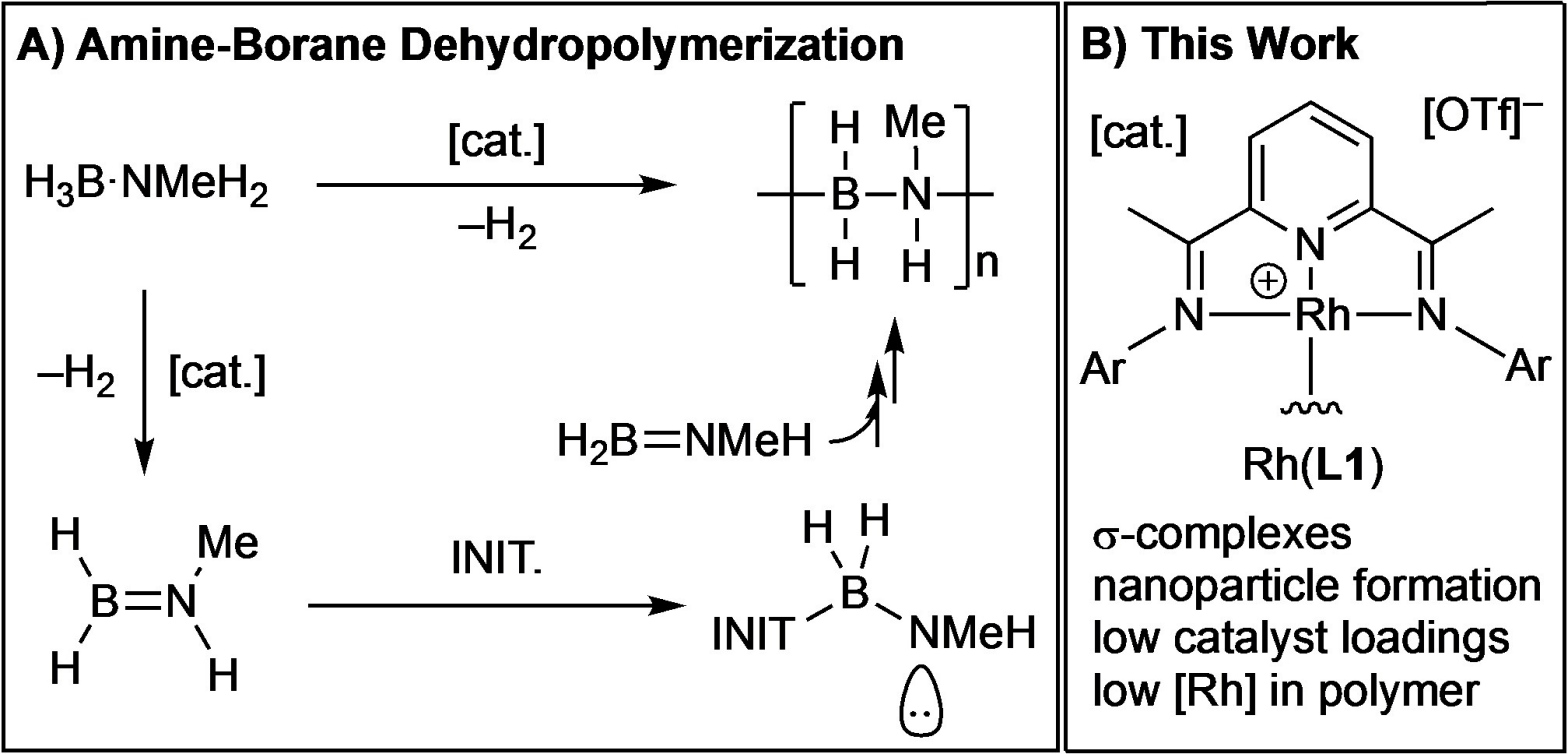
**A)** Amine−borane dehydropolymerization, INIT.=Initiator. **B)** Highlights of this work, Ar=3,5‐^i^Pr_2_C_6_H_3_.

Polyaminoboranes, [H_2_BNRH]_n_, are the main‐group analogs of polyolefins, in which BN‐main chain units replace CC.[^1^, ^2^] However, compared to their organic polymer analogs, polyaminoboranes are essentially unexplored materials.[^3^, ^4^, ^5^] As well as the fundamental interest associated with the generation of main‐group polymers, polyaminoboranes promise to be processable pre‐ceramics to hex‐BN, an important advanced material because of its favorable electronic (wide band gap), materials (tensile strength, thermal management), and chemical (oxidation resistant) properties.[^6^, ^7^, ^8^, ^9^] While non‐catalytic routes to polyaminoboranes are known,[^10^, ^11^, ^12^] metal‐catalyzed routes currently offer the best opportunities for control over the polymerization process.[^2^, ^13^] The catalytic dehydropolymerization of primary amine−boranes, H_3_B ⋅ NRH_2_ (R=alkyl, principally R=Me) was first described in 2008 using the Ir(^t^Bu−POCOP)H_2_ catalyst,[^14^, ^15^, ^16^] and since then a wide variety of catalyst systems have been reported.^[13]^ One current generally accepted mechanism for dehydropolymerization is a cascade‐like^[17]^ polymerization, where the metal centre first dehydrogenates amine−borane to form a very reactive aminoborane,^[11]^ which then undergoes a nucleophilic head to tail chain‐growth polymerization via an amido‐end group of the growing chain, likely initiated by a metal hydride or free amine.[^18^, ^19^, ^20^] Scheme 1A exemplifies this with the H_3_B ⋅ NMeH_2_ pre‐monomer to form *N*‐methylpolyaminoborane, [H_2_BNMeH]_n_. Alternative step‐growth‐like mechanisms have also been reported.[^21^, ^22^] While much emphasis has been placed on studies to understand the mechanism, with the intention of developing controlled dehydropolymerizations,[^21^, ^23^, ^24^, ^25^, ^26^, ^27^, ^28^, ^29^, ^30^] separation of the transition metal catalyst from the isolated polyaminoborane end‐product has been relatively overlooked.[^26^, ^27^, ^30^] If polyaminoboranes are to be developed as viable preceramic precursors for advanced BN materials the control, and mitigation, of the residual metal content is important.

We have previously reported the synthesis of [Rh(**L1**)(η^2^:η^2^‐H_3_B ⋅ NMe_3_)][BAr^F^_4_] [**L1**=2,6‐bis‐[1‐(2,6‐diisopropylphenylimino)‐ethyl]pyridine, Ar^F^=3,5‐(CF_3_)_2_C_6_H_3_] in which the amine−borane interacts with the metal center through two 3c–2e Rh⋅⋅⋅H−B interactions.^[31]^ No onward reaction was observed due to the requirement for an N−H group for dehydrocoupling.^[2]^ As closely related complexes Rh(**L2**)Cl act as precursors to nanoparticles for the dehalogenation of chloroarenes [**L2**=2,6‐bis‐[1‐(4‐(CF_3_)phenylimino)ethyl]pyridine]^[32]^ we were interested to see if these metal‐ligand motifs also promoted amine−borane dehydropolymerization of primary amine−boranes to form polyaminoborane, possibly via nanoparticle formation given the well‐known role of amine−boranes in promoting reduction of metal centers.[^33^, ^34^, ^35^, ^36^, ^37^] The formation of nanoparticles would potentially allow for ease of separation from the polyaminoborane product.

In this contribution we report the use of a pre‐catalyst based upon Rh(**L1**) to selectively dehydropolymerize H_3_B ⋅ NMeH_2_ to form [H_2_BNMeH]_n_, Scheme 1B. The formation of Rh nanoparticles (1‐5 nm) at the end of catalysis is observed, and separation of these from the polymer results in very low (6  μg/g) residual metal loadings in the isolated polymer product. The activation of the pre‐catalyst from intermediate σ‐amine−borane complexes, via base‐promoted hydride transfer processes, is discussed.[^38^, ^39^] We also report the depolymerization of [H_2_BNMeH_2_]_n_, formed with this catalyst system, and others, to selectively give one isomer of cyclic 1,3,5‐trimethyltriborazane.

## Results and Discussion

### Synthesis of σ‐amine−borane and related complexes

**Scheme 2:**
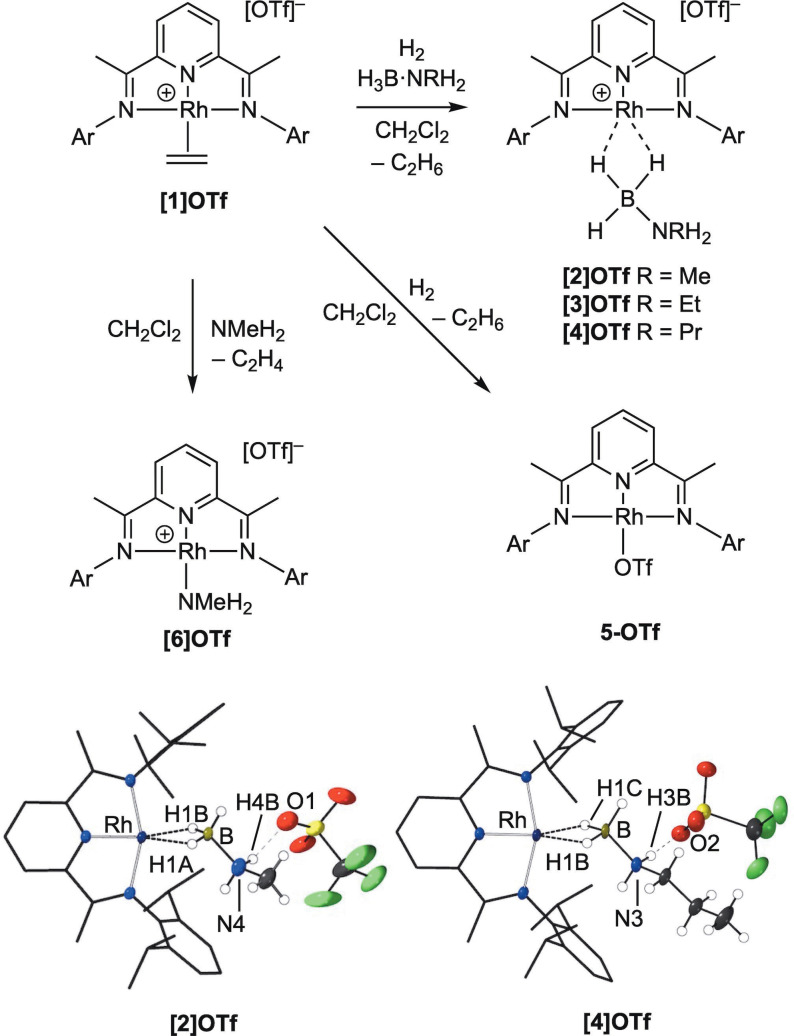
Synthesis of complexes [Rh(**L1**)(ligand)][OTf] and molecular structures of **[2]OTf** and **[4]OTf**. Ar=3,5‐^i^Pr_2_C_6_H_3._ Selected structural metrics (Å): **[2]OTf** Rh−B, 2.253(4); B−N4, 1.581(7); Rh−H1A, 1.89(3); Rh−H1B, 1.88(3); H4B⋅⋅⋅O1, 1.986(4). **[4]OTf** (one of two independent cations in the unit cell) Rh−B, 2.290(4); B−N3, 1.584(5); Rh−H1B, 1.90(5) Å; Rh−H1C, 1.89(4), H3B⋅⋅⋅O2, 2.053(3).

We have previously reported the synthesis of [Rh(**L1**)(η^2^:η^2^‐H_3_B ⋅ NMe_3_)][BAr^F^_4_] by halide abstraction from Rh(**L1**)Cl using Na[BAr^F^_4_]/H_3_B ⋅ NMe_3_. Wishing to use the cheaper and more readily accessible triflate anion (OTf) for this study we have modified this preparative approach to start from the versatile ethene precursor [Rh(**L1**)(H_2_C=CH_2_)][OTf], **[1]OTf** (Scheme 2).

**[1]OTf** is synthesized in good isolated yield (93 %) as analytically pure brown crystals by combining [Ag(**L1)**][OTf]^[31]^ and [Rh(H_2_C=CH_2_)Cl]_2_ in CH_2_Cl_2_ solution, and was characterized by NMR spectroscopy and single‐crystal X‐ray diffraction (Supporting Materials). The resulting data are very similar to those reported for [Rh(**L1**)(H_2_C=CH_2_)][BAr^F^_4_] by Brookhart.^[40]^ The solid‐state structure shows no close approach between the OTf anion and the cation.

Addition of H_2_ to **[1]OTf** in CH_2_Cl_2_ solution in the presence of one equivalent of primary amine−boranes, H_3_B ⋅ NRH_2_, stirring overnight and recrystallization by addition of pentane results in the isolation of the new σ‐amine−borane complexes [Rh(**L1**)(η^2^:η^2^‐H_3_B ⋅ NRH_2_)][OTf] as dark green crystalline materials in moderate to good yield as grown from pentane/CH_2_Cl_2_: R=Me, **[2]OTf** (77 % yield); Et, **[3]OTf** (39 %); ^*n*^Pr, **[4]OTf** (57 %). In solution these complexes are dark blue/green. These new complexes decompose slowly in CD_2_Cl_2_ solution over the timescale of recrystallization, so that – for example – after 3 days at 298 K 20 % conversion to the triflate‐bound complex **5‐OTf** (see below for synthesis) is observed by NMR spectroscopy starting from **[2]OTf**. An NMR spectrum taken after 1 h shows no detectable decomposition. These new complexes have been fully characterized by NMR spectroscopy and single‐crystal X‐ray diffraction (Supporting Materials), Scheme 2 shows the molecular structures of **[2]OTf** and **[4]OTf**. These show that the amine−borane binds in an η^2^:η^2^ motif through two 3c–2e Rh⋅⋅⋅H−B interactions with the Rh(I) center (B−*H* atoms were located in the final difference map), being very similar to that reported for [Rh(**L1**)(η^2^:η^2^‐H_3_B ⋅ NMe_3_)][BAr^F^_4_],^[31]^ e.g Rh⋅⋅⋅B=2.306(5) Å, cf. 2.254(4) and 2.290(4) Å in **[2]OTf** and **[4]OTf** respectively. The triflate anion shows a hydrogen bonding interaction with an N−H proton (~2.0 Å). In the solution (CD_2_Cl_2_) ^1^H NMR spectra the Rh⋅⋅⋅H_3_B interactions are observed as broad, relative integral 3H, signals at ~δ −2, for all three complexes – indicative of rapid site exchange between the three B−H groups.^[41]^ Only two ^*i*^Pr methyl, and one methine, environment are observed, consistent with this exchange process. The N−H⋅⋅⋅OTf hydrogen bonding interaction likely persists in solution, as compared with independently synthesized **[2]BAr^F^**_**4**_ (Supporting Materials) the N−H group is observed to be shifted downfield in **[2]OTf** (δ 2.23 versus δ 2.96 respectively), while the chemical shift of the Rh⋅⋅⋅H_3_B interaction is essentially unchanged (δ −1.90). Finally, in the ^11^B{^1^H} NMR spectra, significantly down‐field shifted resonances compared with free H_3_B ⋅ NRH_2_ are observed (~δ −8 versus ~δ −19, see Supporting Materials) indicative of a η^2^:η^2^ interaction.[^31^, ^42^] All of these data are fully consistent with a σ‐amine−borane complex at a Rh(I) center, and are very similar to those reported for [Rh(**L1**)(η^2^:η^2^‐H_3_B ⋅ NMe_3_)][BAr^F^_4_].^[31]^

Addition of H_2_ to **[1]OTf** in the absence of amine−borane results in the isolation of Rh(**L1**)(κ^1^‐OTf), **5‐OTf**, in which the triflate anion now binds to the metal center. **5‐OTf** decomposes in solution under these conditions but could be characterized by in situ NMR spectroscopy (Supporting Materials). A few crystals were also obtained allowing for analysis by single crystal X‐ray diffraction. **5‐OTf** is closely related to Rh(**L3**)(κ^1^‐OTf)^[43]^ [**L3**=2,6‐bis‐[1‐(2,6‐dimethylphenylimino)ethyl]pyridine]. In CD_2_Cl_2_ solution the [OTf]^−^ anion in **5‐OTf** likely remains bound, as comparison with the ^1^H NMR data reported for [Rh(**L1**)][BAr^F^_4_], in which the [BAr^F^_4_] anion does not bind and most likely CD_2_Cl_2_ acts as a ligand,^[44]^ shows a different set of chemical shifts for each environment. However, that complex **5‐OTf** is not immediately observed when amine−borane, ethene or NMeH_2_ (see below) are present shows that the [OTf]^−^ anion is not competitive for coordination with the metal center compared these ligands. **5‐OTf** is thus unlikely to be relevant on the timescale of catalysis (hours).

Of relevance to the catalytic manifold (see below) is the isolation, as dark brown crystals, of the methylamine adduct [Rh(**L1**)(NMeH_2_)][OTf], **[6]OTf**, by addition of excess NMeH_2_ to **[1]OTf**. The solid‐state structure of **[6]OTf** shows that the [OTf]^−^ anion hydrogen bonds to the NMeH_2_ group (Figure S7). This is not retained in solution to a significant degree as in the ^1^H NMR spectrum of **[6]OTf** the NMe*H*_2_ signal is essentially unchanged compared with the [BAr^F^_4_]^−^ analog: δ 1.86/1.87 respectively.

**Scheme 3:**
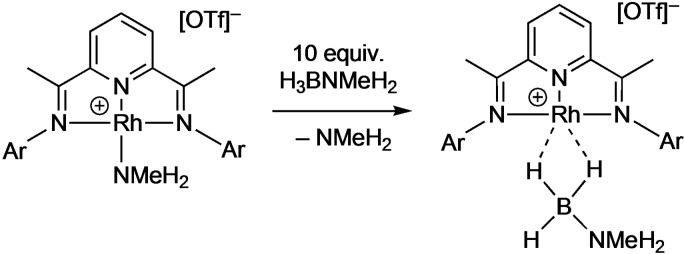
Reaction between **[6]OTf/**H_3_B ⋅ NMeH_2_ and **[2]OTf**/NMeH_2_.

Addition of ten equivalents of H_3_B ⋅ NMeH_2_ to the amine complex **[6]OTf** in a frozen CD_2_Cl_2_ solution and thawing to 298 K over 2 min results in the initial observation of amine−borane complex **[2]OTf** as the major component. These data show that H_3_B ⋅ NMeH_2_ will displace bound NMeH_2_ (Scheme 3). However, further studies were hampered by a slower (minutes) change to a dark solution indicative of colloidal rhodium, and multiple, overlapping, signals being observed in the aromatic region of the ^1^H NMR spectrum that are, as yet, unidentified.

### Dehydropolymerization of H_3_B ⋅ NMeH_2_ using [2]OTf and [6]OTf in 1,2‐F_2_C_6_H_4_ solution: kinetics and role of amine

**Figure 1:**
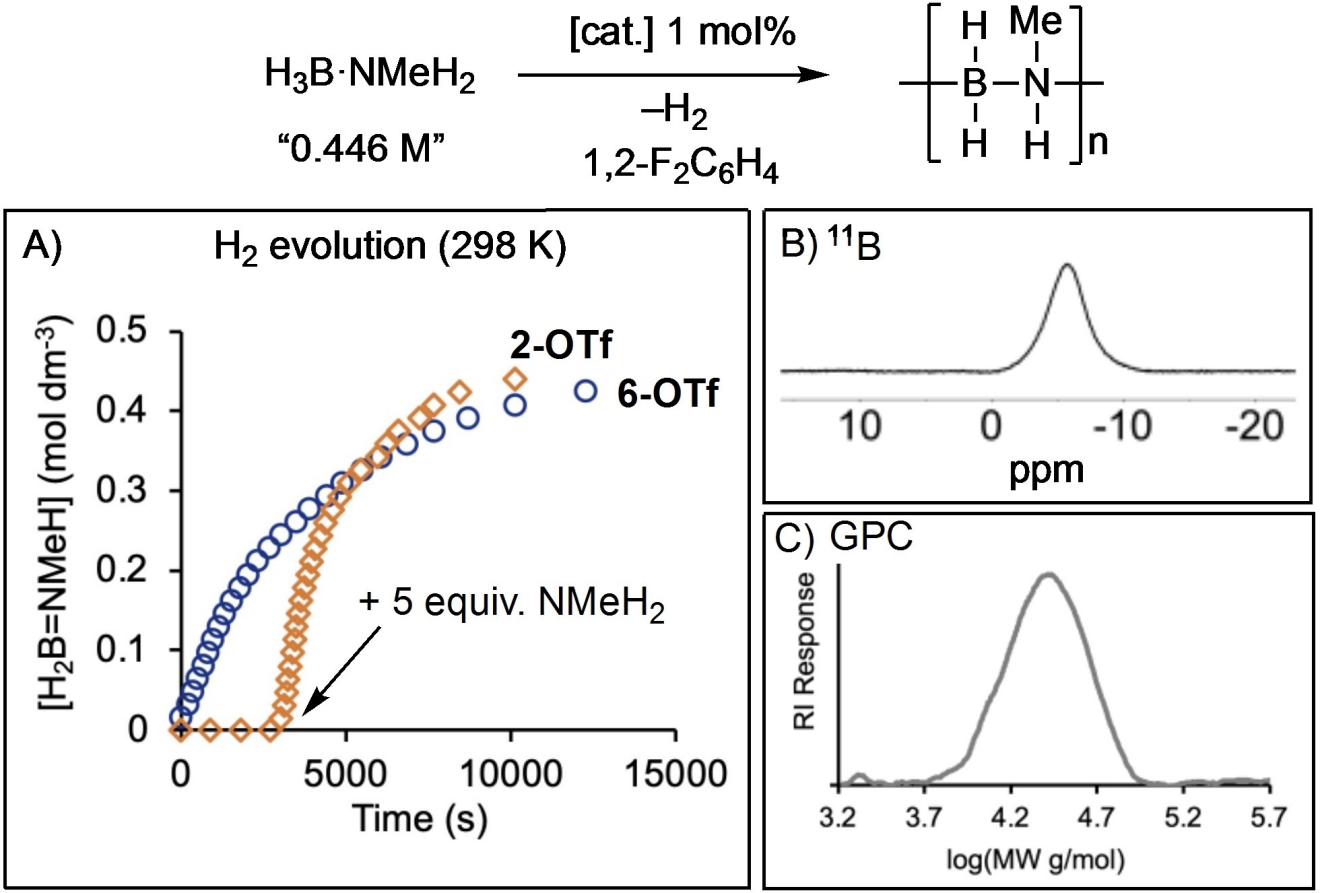
**A)** Time/course plot for H_2_ evolution (stated as the equivalent concentration in H_2_B=NMeH) for use of **[2]OTf** (◊) and **[6]OTf** (**O**) as pre‐catalysts. 1 mol %, [H_3_B ⋅ NMeH_2_]=0.446 M slurry in 1,2‐F_2_C_6_H_4_ **). B)** ^11^B NMR spectrum of polymer isolated using pre‐catalyst **[6]OTf**; **C)** GPC trace of isolated polymer using pre‐catalyst **[6]OTf**.

Initial catalytic studies on the dehydropolymerization of H_3_B ⋅ NMeH_2_ focused in using σ‐amine−borane complex **[2]OTf** as a pre‐catalyst in 1,2‐F_2_C_6_H_4_ solvent, as used previously for other cationic dehydropolymerization systems.[^27^, ^29^, ^30^, ^45^] Catalyst loading was 1 mol % and the nominal concentration of H_3_B ⋅ NMeH_2_ was 0.446 M (~50 mg), although its poor solubility in 1,2‐F_2_C_6_H_4_ meant that the reaction was, in fact, a slurry, with a limiting concentration of ~0.223 M, as reported previously.^[30]^ H_2_ release was measured at 25 °C eudiometrically, as a proxy for the formation of “real” monomer amino−borane, H_2_B=NMeH. The resulting time/conversion plot is shown in Figure 1. To our surprise, complex **[2]OTf** was inactive for at least 45 min, with the solution remaining a dark blue/green color. Speciation measurements using ^1^H NMR spectroscopy were frustrated by the protio‐solvent used and the excess of H_3_B ⋅ NMeH_2_. However a broad, low relative intensity, signal was observed at ~δ −2 assigned to **[2]OTf**. It has previously been shown that induction periods observed with dehydrocoupling/dehydropolymerization of H_3_B ⋅ NMeH_2_, and related amine−boranes, are associated with base‐promoted hydride transfer processes from cationic σ‐amine−borane complexes[^38^, ^46^] to form the active, neutral hydride, catalysts;[^19^, ^26^, ^27^, ^39^, ^47^] although there are cases where such hydride transfer can also form a less active catalyst.^[29]^ For H_3_B ⋅ NMeH_2_ dehydropolymerization the base is NMeH_2_, that comes from the slow dissociation^[48]^ of H_3_B ⋅ NMeH_2_, or possibly trace NMeH_2_ in the starting material – related to that recently proposed for analogous phosphine−borane dehydropolymerization.^[49]^ Generally, addition of excess amine or starting from an amine complex itself, reduces the induction period by quickly generating the active catalyst. Excess amine can also stop the formation of inactive borohydride complexes.^[23]^

In the system under discussion here, additional NMeH_2_ also has such a positive effect. Starting from the amine−borane complex **[2]OTf** (1 mol %) addition of 5 equivalents of NMeH_2_ after 45 min resulted in immediate H_2_ production (Figure 1A). On addition of NMeH_2_ there is also a change from the dark blue/green color of **[2]OTf** to a dark red color that then turns dark brown over 10 min. A dark grey suspension eventually forms over the next 15 min, suggesting the formation of Rh‐nanoparticles. A eudiometric experiment demonstrates that ~1 equivalent of H_2_ is released over 2.5 h. At the end of catalysis a dark‐grey suspension and a pale‐yellow supernatant remains. ^11^B NMR spectroscopy of the crude product showed that a small amount of *N,N,N*‐trimethylcyclotriborazane and other BN‐containing products were formed alongside *N*‐methylpolyaminoborane, that result from unselective dehydrocoupling. The resulting polymer, [H_2_BNMeH]_n_, was isolated without these side products as a grey solid, by precipitation into pentane (68 % yield), the ^11^B NMR spectrum of which (CDCl_3_) shows the expected broad signal at δ −6.6 (Figure 1B).[^14^, ^15^] Analysis by GPC (Gel Permeation Chromatography, relative to polystyrene standards) showed a broadly monomodal distribution, *M*_n_ 29,700 g/mol, Ð=1.4, (Figure 1C). Consistent with the role of amine in productive catalysis, starting from **[6]OTf** (1 mol %) results in immediate H_2_ evolution, and after 3.3 h 0.95 equivalents of H_2_ has been released. The resulting isolated polymer was slightly shorter than with **[2]OTf**: *M*_n_ 20,500 g/mol, Ð=1.4. Added NMeH_2_ resulting in higher degrees of polymerization has been noted before.^[47]^ Similar color changes were noted using **[6]OTf**, and at the end of catalysis a grey suspension also remains. While **[6]OTf** evolves H_2_ with close to first order kinetics, **[2]OTf** does not, and we are reluctant to over‐interpret the data given the evolving system over the timeframe of catalysis and the limited solubility of H_3_B ⋅ NMeH_2_ in 1,2‐F_2_C_6_H_4_. However, the maximum rate measured for **[2]OTf** after excess NMeH_2_ addition is 2.9(1)×10^−4^ M/s, considerably faster than for **[6]OTf** 1.2(5)×10^−4^ M/s, pointing to the role of NMeH_2_ in generating the active catalyst. Recharging the end‐of‐catalysis suspension with 100 equivalents H_3_B ⋅ NMeH_2_ (relative to starting **[6]OTf**) resulted in H_2_ production at a similar rate as observed previously (Figure S41). The crude polymer isolated at the end is also similar: *M*_n_ 26,800 g/mol, Ð=1.5. This shows that the catalyst remains active at the end of polymerization and that the polymerization is not living.

Overall, these data point to a complex set of precatalyst evolution events, the likely formation of nanoparticles, the involvement of NMeH_2_ in catalyst activation, and the selective production of polyaminoboranes.

### Evidence for the formation of colloidal Rh and comments on the activation mechanism

**Figure 2:**
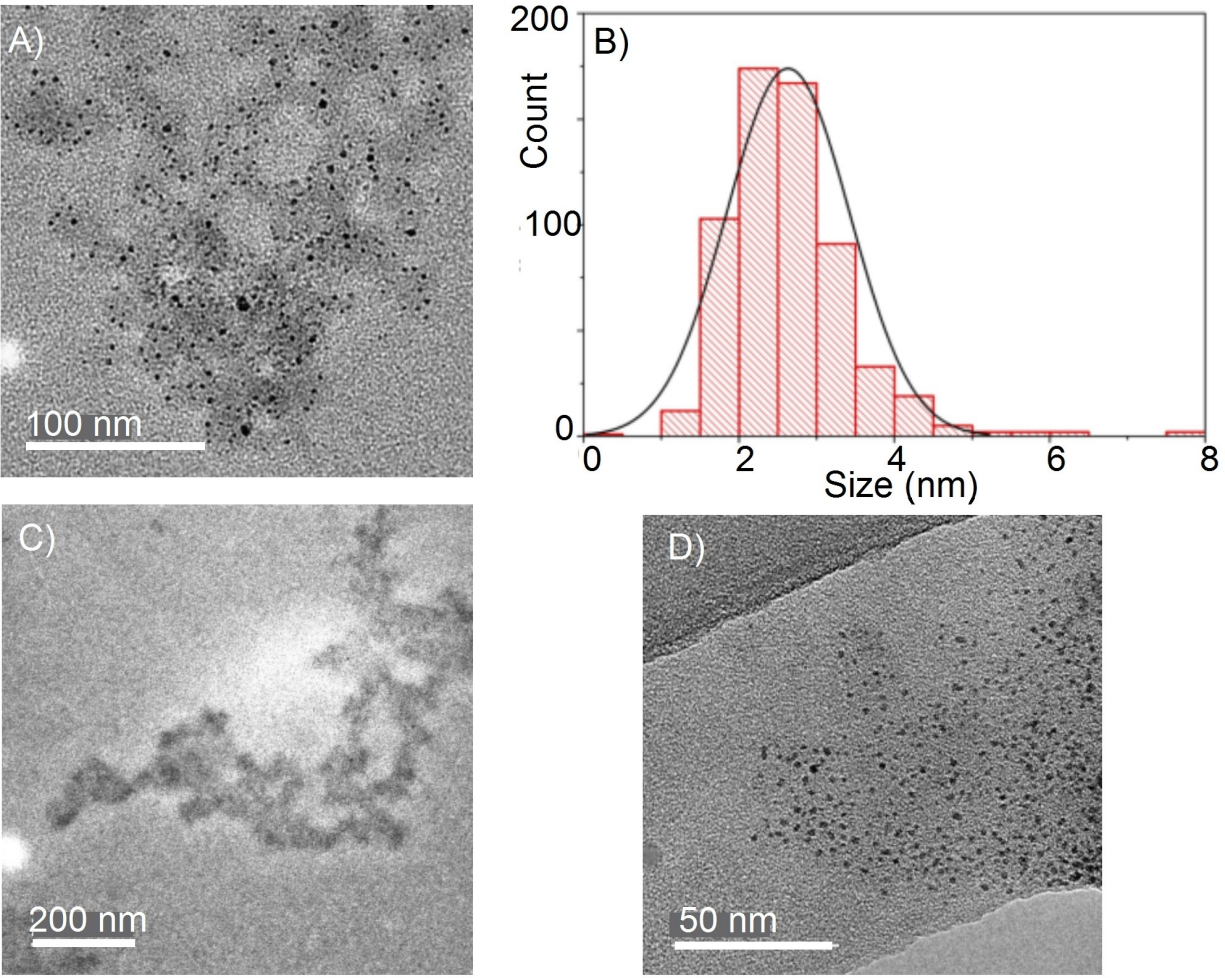
TEM analysis of [H_2_BNMeH]_n_ produced by **[6]OTf**. **(A)** Crude polymer; **(B)** Size distribution histogram; **(C)** Purified polymer; **(D)** Filter cake from 0.2 μm filter.

The formation of a grey precipitate at the end of catalysis, coupled with the evolution of the color of the catalyst solution prior to this over a 25‐minute period, suggested an induction period in which colloidal Rh was formed.^[50]^ Analysis of the isolated polymer generated using **[6]OTf** by TEM (*V*_acc_=200 kV, sample dispersed in THF solution) showed the presence of discreet, evenly dispersed, nanoparticles (Figure 2A) in a polymer matrix, with a size distribution of between 1 nm and 5 nm, with the 2–3 nm being the most common (Figure 2B). Filtration through a 0.2 μm PTFE filter resulted in a white, THF‐soluble material, that analyzed for [H_2_BNMeH]_n_ (^11^B NMR spectroscopy). TEM analysis of the resulting solid (Figure 2C) showed the absence of nanoparticles, while TEM analysis of the filter clearly showed that nanoparticles had been captured by this process (Figure 2D). Analysis of precatalyst **[6]OTf** using TEM resulted in very different material, of much larger size, formed from multiple twinned particles, that also displayed clear lattice fringes of ~0.23 nm assigned to the (111) plane of *fcc* Rh (Figures S67/68).^[51]^ So while it is likely that **[6]OTf** undergoes degradation in the beam under the conditions used, that it is clearly a molecular species prior to this and the particles formed are very different from those generated in catalysis suggests that the polymer entrained nanoparticles observed come from precatalyst evolution rather than from beam‐degradation. Beam‐degradation of molecular precatalysts has been reported previously.[^35^, ^52^, ^53^]

While evidence for the formation of nanoparticles at the end of catalysis is unequivocal, whether these are the active catalytic species is less certain. The Hg‐drop test (albeit a potentially flawed test[^53^, ^54^, ^55^]) showed no significant decrease in reactivity when added after ~25 % conversion. This test has been shown to work in a positive sense for amine−borane dehydrocoupling of H_3_B ⋅ NMe_2_H to identify colloidal Rh when using [Rh(COD)Cl_2_]_2_ as a precatalyst.[^33^, ^52^] While this may suggest a homogeneous catalyst operates, it may also well be that polymer‐encapsulated nanoparticles form (as evident from TEM studies) that are resistant to forming an Hg−amalgam,^[56]^ similar to the attenuation in catalytic activity observed with organic polymer‐coated nanoparticles.^[57]^ Addition of sub‐stoichiometric PMe_3_ (0.3 equivs.), a test for heterogeneous catalysis,^[53]^ did initially halt catalysis – indicative of nanoparticle catalysis. However, after 20 min activity steadily resumed (Figure S46). We suggest this is due to, irreversible, borane‐promoted, phosphine dissociation revealing the active catalyst. Strong donor ligands, similar to the PMe_3_ added here, are known to react with polyaminoboranes by chain‐scission,^[58]^ while slow dissociation of H_3_B ⋅ NMeH_2_ would also provide a source of “BH_3_”. Addition of dibenzocyclooctatetaene (dbcot) to catalysis after 25 % conversion did not slow turnover (Figure S47). As this tub‐shaped diene has been shown to coordinate strongly with, and thus inhibit, homogeneous catalysts in low oxidation states this is additional evidence for the formation of colloidal Rh as the principal catalyst.[^53^, ^59^, ^60^] Finally, filtration of the post‐catalysis mixture though 0.2 μm filter, and use of the filtrate in catalysis restarted turnover, but at a considerably reduced rate. While this may point to a soluble homogeneous component to catalysis, TEM shows nanoparticles are formed in the 1–5 nm size regime, and it is possible that these would not be captured by filtration through a 0.2 μm filter if not entrained in polymer. Analysis of the crude mixture at the end of catalysis by ESI‐MS showed the major component to be partially hydrogenated free ligand, Dipp(N=CMe)(C_5_H_3_)(CMeHNH)Dipp, m/z=483.5 (obs.), 483.4 (calc.). Analysis by ^1^H NMR spectroscopy was frustrated by overlap with signals due to polymer and residual 1,2‐F_2_C_6_H_4_, and there was little evidence for the precursor **[6]OTf**.

Ambiguity as to the active species has been noted previously by Esteruelas and co‐workers, in the use of Rh(**L2**)Cl for the dehalogenation and hydrogenation of chloroarenes.^[32]^ Here, Rh‐nanoparticle formation is unequivocally established using TEM, while the Hg‐test results in a significant, but not complete, drop in activity which was used as evidence for a homogeneous component to the system. Interestingly, in this system, partially hydrogenated ligand was also observed, which is also suggested to stabilize the Rh‐nanoparticles.

**Scheme 4:**
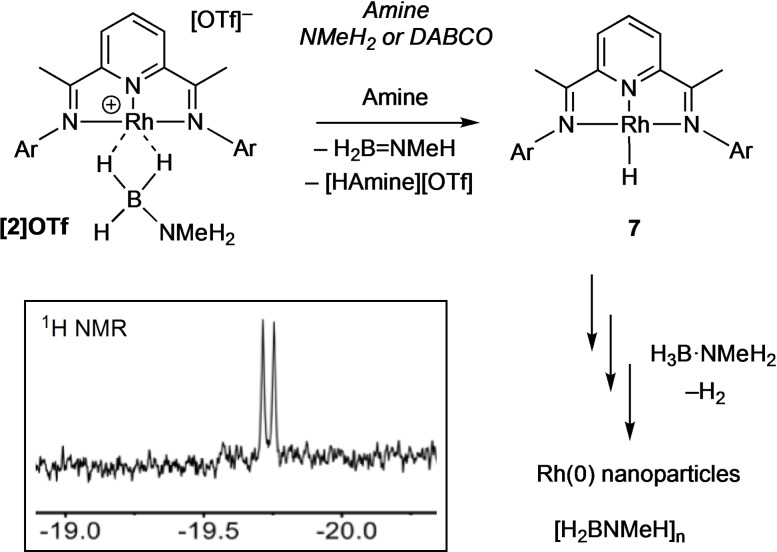
Generation of complex **7** and suggested formation of nanoparticles. Inset shows hydride region of ^1^H NMR spectrum (600 MHz) after 30 min acquisition time (1024 scans). Ar=3,5‐^i^Pr_2_C_6_H_3_.

The speciation associated with the activation of the precatalyst, especially the dark‐red species observed at very early stages, has been studied. While we propose that in situ generated NMeH_2_ acts as a base to deprotonate the N−H bond in [Rh]⋅⋅⋅H_3_B ⋅ NMeH_2_, resulting in hydride transfer to form a neutral active catalyst, addition of precisely controlled amounts of this volatile amine (b.p. −6.3 °C, used in a 2 M solution in THF) is challenging. Instead, the solid‐base DABCO (triethylenediamine) was added to a mixture of **[6]OTf** and H_3_B ⋅ NMeH_2_, in an overall 2 : 1 : 2 ratio, in 1,2‐F_2_C_6_H_4_ solution in an NMR tube under an Ar atmosphere. Under these conditions dark blue/green **[2]OTf** immediately forms (Scheme 3), as identified by a characteristic high field signal observed at δ −2 in the ^1^H NMR spectrum. Over the course of 5 min, under these conditions of excess amine, this signal disappeared to be replaced by a very weak signal at δ −19.7. Over the same period the solution turned to dark red, and then over the next 30 min turned dark brown – as observed in catalysis. An extended ^1^H NMR acquisition was made over this time that revealed the weak signal at δ −19.7 as a doublet coupling to ^103^Rh [*J*(RhH) 24.7 Hz]. We assign this complex to the hydride species Rh(**L1**)H, **7** (Scheme 4), likely being formed at steady state from **[2]OTf** and decomposing to Rh nanoparticles. Related group 9 pincer hydride complexes are known, for example Rh(^t^Bu−Xantphos)H [δ −19.28, *J*(RhH)=34.4 Hz] (green)^[61]^ or Rh(PONOP)H [δ −9.60, *J*(RhH)=19.5 Hz] (red).^[39]^ However, the low intensity of this signal suggests that this is not the major species in solution, and the red‐colour may be due to other non‐hydride containing molecular species or soluble Rh(0) nanoparticles. Nevertheless the identification of **7** is consistent with activation of the precatalyst by hydride transfer from the borane in **[2]OTf**, as reported for other cationic σ‐amine−borane complexes.[^38^, ^39^, ^46^] Once formed, under conditions of catalysis, ligand dissociation (possibility promoted via partial hydrogenation), and the formation of hydride‐bridged multimetallic species would eventually lead to nanoparticle formation via reductive loss of H_2_. Consistent with this red‐solution being catalytically active, when it is transferred to a flask containing 200 equivalents H_3_B ⋅ NMeH_2_ in 1,2‐F_2_C_6_H_4_ (0.5 mol % effective catalyst loading) catalysis started immediately (Figure S38). After 5 h ~1 equivalent of H_2_ had been released, and precipitation into pentanes resulted in the isolation of crude, grey colored, polymer of comparable molecular weight to when using **[6]OTf** as a precatalyst: *M*_n_ 24,500 g/mol, Ð=1.5.

Overall, these data and observations are ambiguous to whether catalysis is solely due to nanoparticles, or if a molecular component such as **7** also contributes – especially during the early stages of catalysts. Whatever the precise nature of the active catalyst, that at the end of catalysis nanoparticles are formed allows for an expedient purification procedure, as described next, to remove residual metal from the polymer. Dehydropolymerization of H_3_B ⋅ NMeH_2_ has previously been reported to be promoted by heterogeneous catalysts, i. e. skeletal nickel,^[62]^ Rh/Al_2_O_3_.^[15]^ Notably [Rh(COD)Cl]_2_ has been reported to be rapidly reduced to colloidal Rh(0) in the presence of H_3_B ⋅ NMeH_2_ (1 mol % [Rh]_total_) to give polymer of moderate molecular weight (*M*_n_ 42,000 g/mol) but very high dispersity (Ð=10.5).[^15^, ^63^]

### Scale up and polymer purification

**Figure 3:**
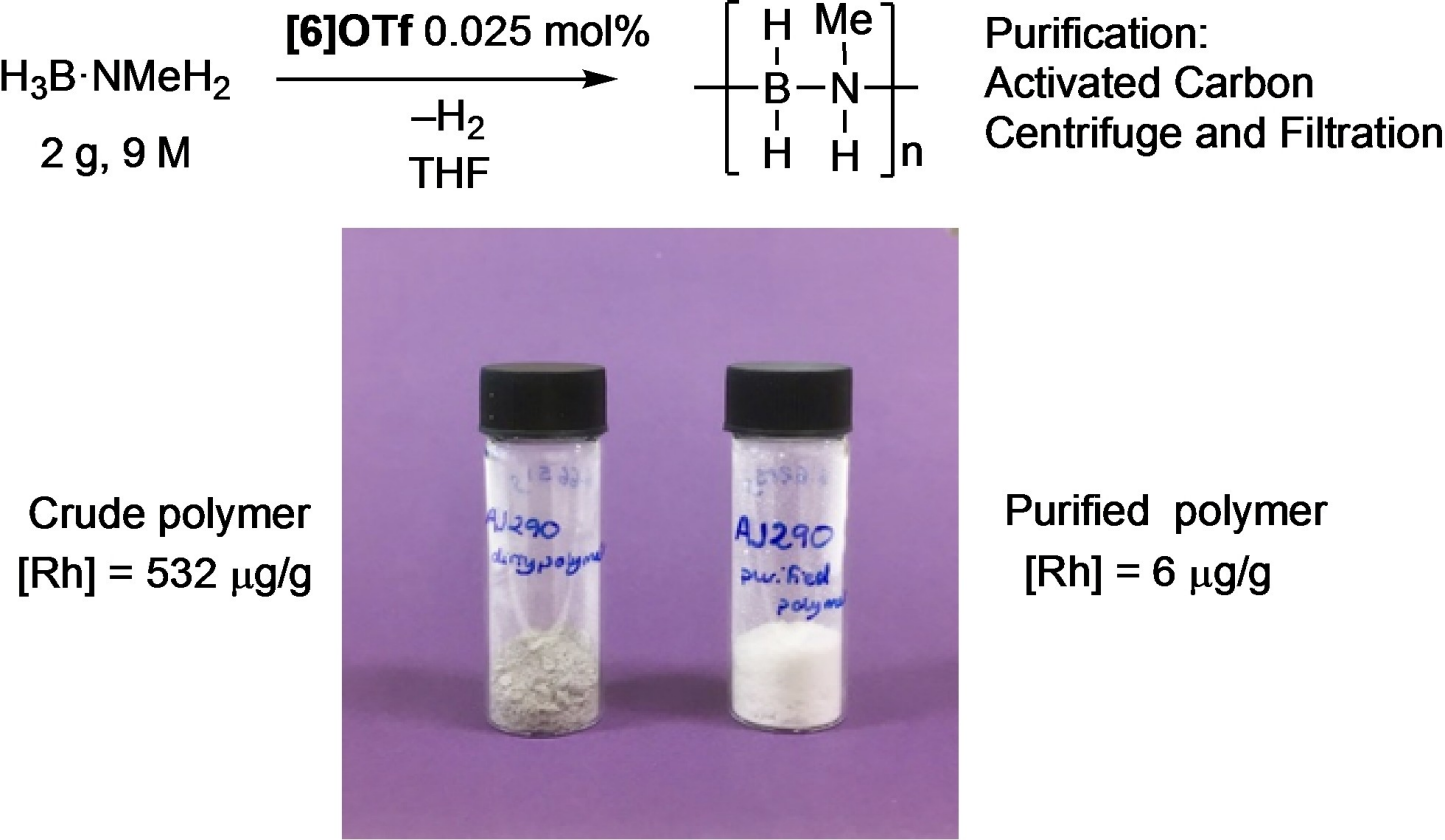
Synthesis and purification of [H_2_B ⋅ NMeH]_n_ on gram scale.

The limited solubility of H_3_B ⋅ NMeH_2_ in 1,2‐F_2_C_6_H_4_ makes this solvent less than suitable for scale up. THF provides significantly better solubility,^[64]^ and we have previously shown that this solvent can be used for production of [H_2_BNMeH]_n_ on 10 g scale.^[28]^ Using **[6]OTf** at low catalyst loading (0.025 mol %) in concentrated THF solution (2 g of H_3_B ⋅ NMeH_2_, in 5 mL THF, ~9 M) resulted in the complete dehydropolymerization to give [H_2_BNMeH]_n_ over 48 h, isolated as a grey powder, *M*_n_ 30,900 g/mol, Ð=1.8. ICP‐MS showed the rhodium content to be 532 μg/g. After significant optimization it was found that residual rhodium was best removed from the crude polymer first by treatment with 1 equivalent w/w of activated carbon and stirred for 30 min in THF (20 cm^3^). Filtration through a 0.2 μm PTFE filter and precipitation from pentane (20 mL) resulted in the isolation, in 46 % yield, of a white solid that analyzed for unchanged [H_2_BNMeH]_n_: *M*_n_ 30,500 g/mol, Ð=1.7, Figure 3. Residual Rh was very low, at 6 μg/g. While the overall yield is moderate, as far as we are aware this is the lowest residual metal content reported in metal‐catalyzed polyaminoborane synthesis,[^26^, ^27^, ^30^] although we note that this analytical metric is often not reported. This methodology thus represents a convenient method to produce *N*‐methylpolyaminoborane with very low residual metal content. Non‐metal/non‐catalytic routes have also been reported.[^10^, ^12^]

### Depolymerization of [H_2_BNMeH]_n_ to form cyclic triborazane, [H_2_BNMeH]_3_: towards chemical repurposing of polyaminoboranes

**Scheme 5:**
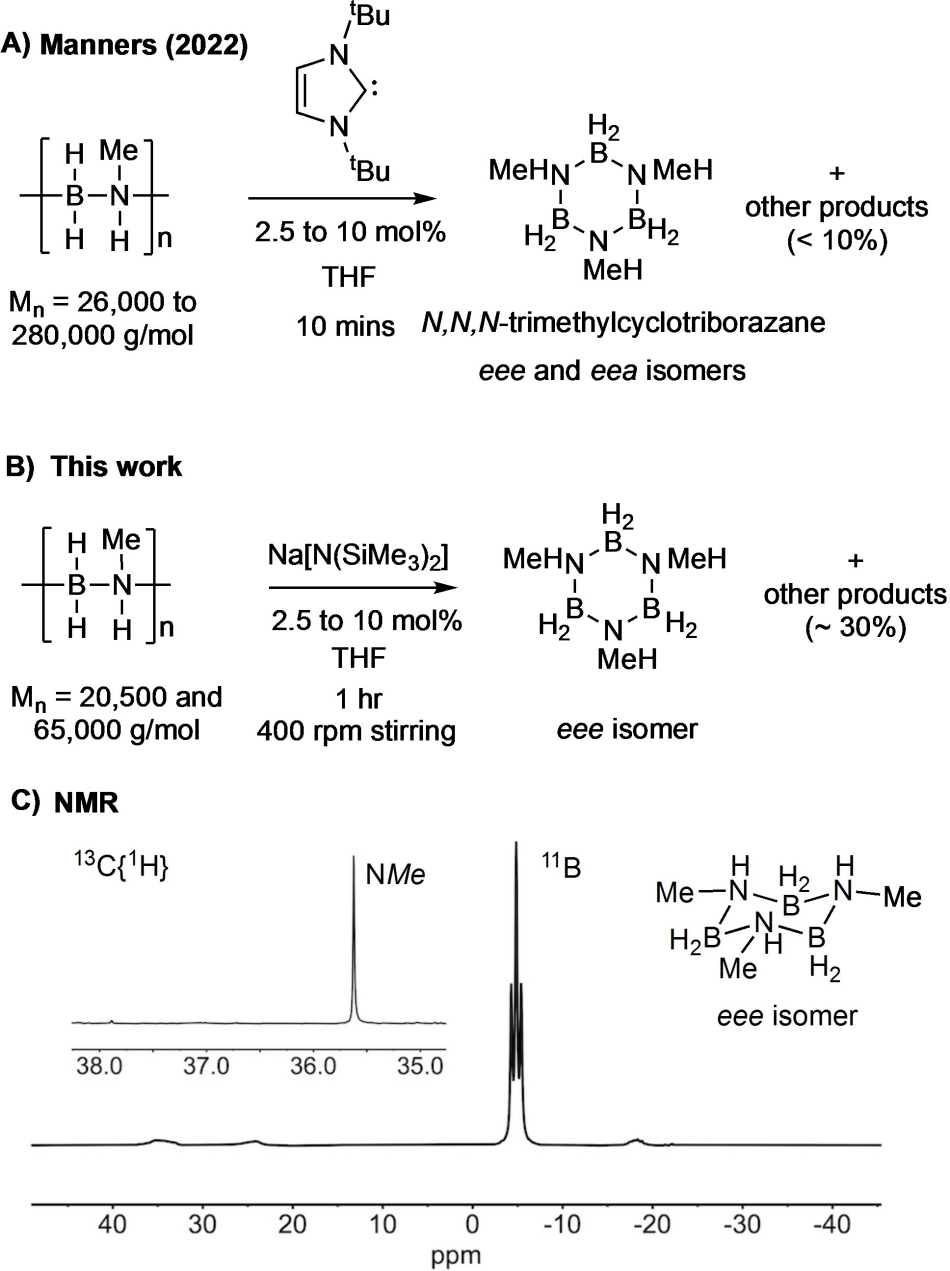
**A)** Manners’ report of depolymerization of [H_2_BNMeH]_n_ using I^t^Bu (I^t^Bu=1,3‐di‐tert‐butylimidazol‐2‐ylidene). **B)** Depolymerization using Na[N(SiMe_3_)_2_]. **C)** ^11^B and ^13^C{^1^H} NMR spectrum of reaction mixture after 1 h showing the selective formation of the *eee*‐isomer of [H_2_BNMeH]_3_ using polymer produced from **[6]OTf**, and 10 mol % Na[N(SiMe_3_)_2_].

The chemical recycling of carbon‐based polymers, or repurposing to other compounds of intrinsic value, using depolymerization strategies, is central to the development of truly closed‐loop circular polymer economies.^[65]^ However, main‐group polymers have received little attention in this regard. Manners’ and co‐workers have recently reported^[66]^ that depolymerization of [H_2_BNMeH_2_]_n_ can be promoted by strongly nucleophilic *N*‐heterocyclic carbenes (e. g. I^t^Bu, I^t^Bu=1,3‐di‐tert‐butylimidazol‐2‐ylidene) to give the cyclic *N,N,N*‐trimethylcyclotriborazane [H_2_BNMeH]_3_,^[67]^ as a mixture of equatorial (*eee*) and equatorial/axial (*eea*) isomers (Scheme 5A) in up to 95 % conversion. We have briefly investigated alternative reagents for this process, using the low‐residual [Rh] polymer generated from both **[6]OTf**, and higher MW polymer produced using our [Rh(^i^Pr−PN^H^P)(NBD)]Cl^[28]^ catalyst that had also been charcoal treated (Scheme 5B). We found sub‐stoichiometric amounts (2.5 mol % to 10 mol %) of the non‐nucleophilic base Na[N(SiMe_3_)_2_] produced the *eee* isomer of [H_2_BNMeH]_3_ on ~70 % selectivity by stirring (400 rpm) in THF for 1 h on a 50 mg scale of polymer (1.1 mmol). The other products include H_3_B ⋅ NHMeBH_2_ ⋅ NMeH_2_ and *N*‐trimethylborazine. Extraction into CDCl_3_ removes the majority of these by‐products.

NMR spectra of the resulting CDCl_3_‐soluble portion of the reaction products showed that a *single* environment was observed in both the ^11^B NMR spectrum [δ −4.8, t, *J*(BH)=107 Hz; lit. −5.4, *J*(BH)=105 Hz, d_6_‐acteone] and the ^13^C{^1^H} NMR spectrum [δ 35.6; Lit. [34].5, d_6_‐acteone], fully consistent with the *eee*‐isomer.^[67]^ This is different from that found by Manners using NHC bases, where mixtures of the *eee* and *eea* isomers were formed. Notably the *eea* isomer displays two signals in the ^13^C{^1^H} NMR spectrum [δ 38.3 and 35.5 d_6_‐acteone^[67]^], which are not observed here.

**Scheme 6:**
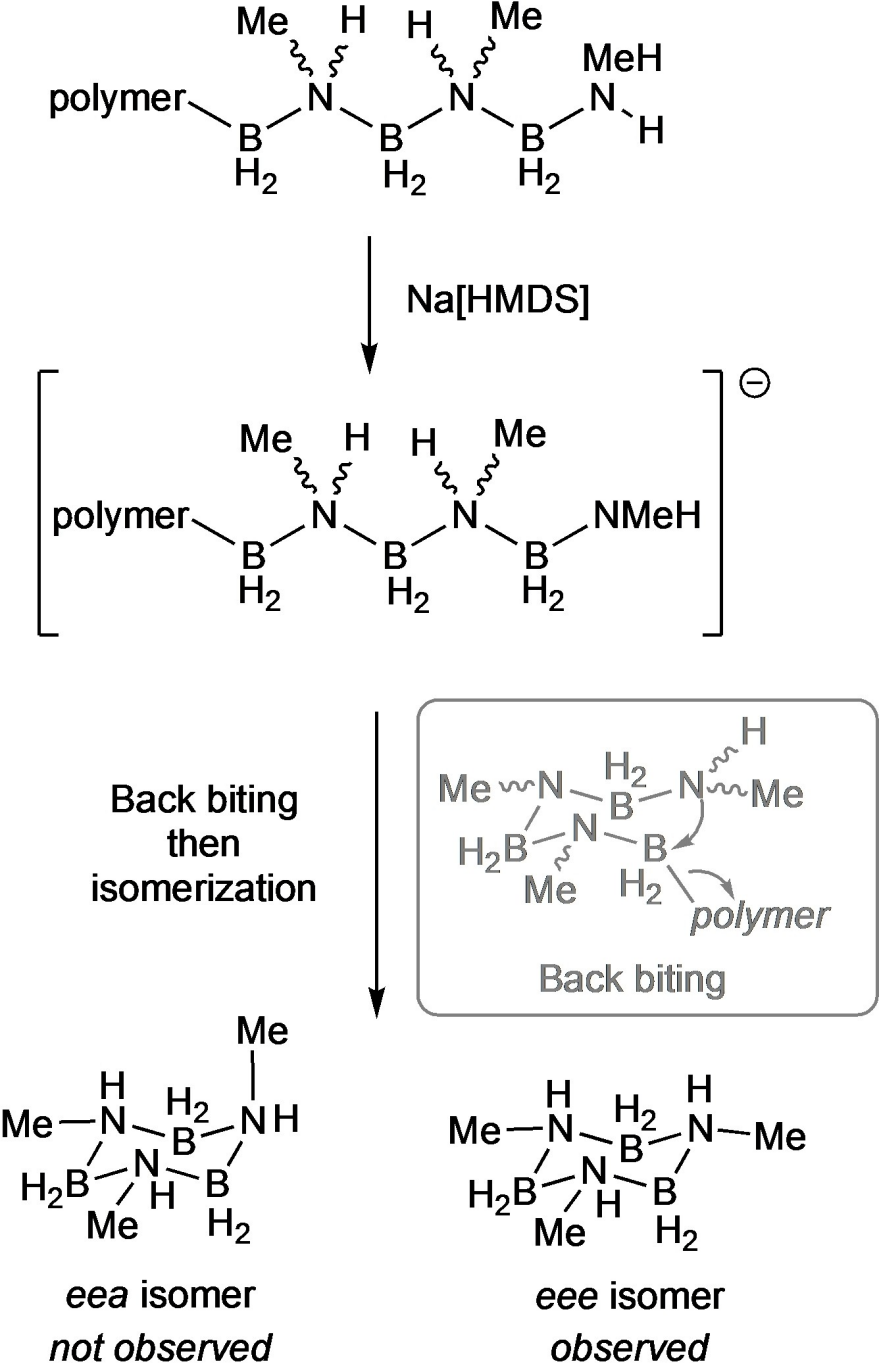
Proposed, simplified, mechanism for the formation of *eee*‐[H_2_BNMeH]_3_. HMDS = [N(SiMe_3_)_2_]^–^

The reasons behind the remarkable selectivity for the *eee* isomer of *N,N,N*‐trimethylcyclotriborazane remain unresolved, especially as the stereochemistry (i. e. tacticity) of the parent polyaminoboranes currently remains opaque – although they are likely atactic, similar to that observed for closely related polyphosphinoboranes.[^68^, ^69^] Our tentative proposal is that deprotonation of an end‐chain ammonium group^[28]^ forms a reactive amido−boryl unit, that then undergoes main‐chain back‐biting in the atactic polymer to form a mixture of *eee* and *eea* isomers of [H_2_BNMeH]_3_ (Scheme 6). A rapid isomerization then occurs. DFT calculations [PBE0/def2‐TZVPP(THF)] show that the *eee* isomer is marginally more stable than the *eea* (by 11 kJ/mol), consistent with this hypothesis. We suggest this isomerization could be base‐promoted, or by reversible transfer dehydrogenation,^[70]^ via a trimethyl‐cyclohexene analog, with concomitantly formed amino−borane, H_2_B=NMeH, that arises from competitive unzipping of the polymer.^[71]^ Calculations suggest that such an unzipping process is both kinetically and thermodynamically accessible.^[18]^ While these elements of depolymerization are closely related to Manners’ proposal for NHC‐promoted formation of [H_2_BNMeH]_3_ from [H_2_BNMeH]_n_,^[66]^ we disfavor a pathway that involves mid‐chain scission, as proposed using NHCs, due to the non‐nucleophilic nature of Na[N(SiMe_3_)_2_]. While the precise mechanistic manifold, and a potential isomerization process, remains to be determined,^[72]^ the overall selectivity observed for the *eee*‐isomer of [H_2_BNMeH]_3_ is remarkable, and suggests opportunities for polyaminoboranes to be chemically repurposed to new BN‐containing materials.

### Dehydropolymerization of H_3_B ⋅ NEtH_2_ and H_3_B ⋅ ^n^PrH_2_

**Scheme 7:**
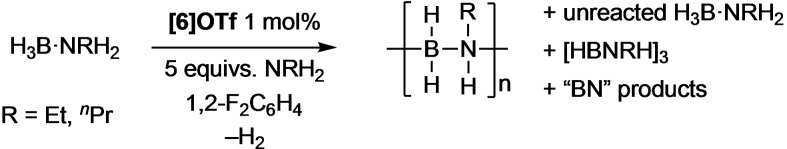
Unselective dehydropolymerization of H_3_B ⋅ NRH_2_ (R=Et, ^*n*^Pr).

Simple alkyl homologues of corresponding *N*‐R‐polyaminoboranes [H_2_B ⋅ NRH]_n_ have been reported using catalytic (R=^*n*^Bu^[14]^) and stoichiometric (R=Et, ^*n*^Pr[^10^, ^12^]) routes. We have used the catalyst/substrate combinations of **[6]OTf**/H_3_B ⋅ NEtH_2_/NEtH_2_ and **[6]OTf**/H_3_B ⋅ N^*n*^PrH_2_/N^*n*^PrH_2_ in an attempt to prepare the corresponding homopolymers [H_2_B ⋅ NEtH]_n_ and [H_2_B ⋅ N^*n*^PrH]_n_, respectively, using the methods developed for H_3_B ⋅ NMeH_2_/**[6]OTf** (Scheme 7). Both substrates underwent dehydropolymerization, releasing just over one equivalent of H_2_ in ~2 hr, at which time the reaction was halted by partial removal of the solvent under vacuum and precipitation into pentane. While GPC data showed the formation of polymer (*M*_n_ 35,500 and 23,400 g/mol respectively), analysis by ^11^B NMR spectroscopy showed that mixtures of oligomer, polymer, unreacted starting material, borazines and other BN‐containing products had formed, in an unselective dehydropolymerization.

## Conclusions

The use of the simple to prepare bis(imino)pyridine rhodium pre‐catalyst [Rh(**L1**)(NMeH_2_)][OTf], **[6]OTf**, results in the efficient dehydropolymerization of H_3_B ⋅ NMeH_2_ to selectively form *N*‐methylpolyaminoborane. The formation of Rh nanoparticles during this process – while adding mechanistic complexity *–* allows for the easy separation of residual catalyst from the polymer, so that only very low levels of [Rh] remain. Such low levels of metal contamination may well be important when looking forward to applications of polyaminoboranes as pre‐ceramic precursors to few‐layer hex‐BN, an exciting electronic material due to its close similarity with graphene, but with a high band gap and thus insulating properties.^[8]^ Moreover, our demonstration that *N*‐methylpolyaminoborane can be depolymerized to selectively produce a single isomer of the corresponding cyclic triborazane suggests opportunities for the chemical repurposing of main‐group polymers – an under explored area. In addition to understanding the mechanism of dehydropolymerization, or the controlled production of polymer on scale, issues such as residual catalyst and recycling are important topics if polyaminoboranes are to establish themselves as technologically useful main‐group polymeric materials.

## Supporting Information

Full experimental details and characterization data are given. In the Supporting Information.

Deposition Numbers 22711697, **[1]OTf**;, 1993415 **[2]OTf**; 1993414, **[2]BAr** ^**F**^_**4**_; 2271698, **[3]OTf**; 2271699, **[4]OTf**; 1993413, **5‐OTf**; 1993419, **[6]OTf**; 2271696, **[6]BAr** ^**F**^_**4**_ contain the supplementary crystallographic data for this paper. These data are provided free of charge by the joint Cambridge Crystallographic Data Centre and Fachinformationszentrum Karlsruhe Access Structures service.

## Supporting Information

The Authors have cited additional references within the Supporting Information.[^73^, ^74^, ^75^, ^76^, ^77^, ^78^, ^79^, ^80^, ^81^, ^82^, ^83^, ^84^, ^85^, ^86^, ^87^, ^88^, ^89^, ^90^]

## Conflict of interest

The authors declare no conflict of interest.

1

## Supporting information

As a service to our authors and readers, this journal provides supporting information supplied by the authors. Such materials are peer reviewed and may be re‐organized for online delivery, but are not copy‐edited or typeset. Technical support issues arising from supporting information (other than missing files) should be addressed to the authors.

Supporting Information

## Data Availability

The data that support the findings of this study are available in the supplementary material of this article.
