# Supplementary material for: Dehydropolymerization of Amine−Boranes using Bis(imino)pyridine Rhodium Pre‐Catalysis: σ‐Amine−Borane Complexes, Nanoparticles, and Low Residual‐Metal BN−Polymers that can be Chemically Repurposed
Source: Chemistry. 2023 Sep 21;29(60):e202302110. doi: 10.1002/chem.202302110 (PMC10947130; doi:10.1002/chem.202302110)
Supplement: Supplementary file 1 — Supporting Information [file CHEM-29-0-s001.pdf]

Chemistry–A European Journal

Supporting Information

Dehydropolymerization of Amine–Boranes using Bis(imino)pyridine Rhodium Pre-Catalysis: σ-Amine–Borane Complexes, Nanoparticles, and Low Residual-Metal BN–Polymers that can be Chemically Repurposed

Mathew J. Cross, Claire N. Brodie, Dana G. Crivoi, Joe C. Goodall, David E. Ryan, Antonio J. Martínez-Martínez, Alice Johnson,* and Andrew S. Weller*

Table of Contents

1. Experimental 3

1.1. General Procedures 3

1.2. Syntheses 4

1.2.1. Synthesis of [1]OTf 4

1.2.2. Synthesis of [2]OTf 5

1.2.3. Synthesis of [2]BAr$^F_4$ 6

1.2.4. Synthesis of [3]OTf 7

1.2.5. Synthesis of [4]OTf 7

1.2.6. Synthesis of 5-OTf 8

1.2.7. Synthesis of [6]OTf 9

1.2.8. Synthesis of [6]BAr$^F_4$ 10

1.3. Crystallography 12

1.3.1. Additional crystallographic details 12

1.3.2. Crystallographic tables 14

1.4. NMR spectra 16

1.4.1. NMR spectra for [1]OTf 16

1.4.2. NMR spectra for [2]OTf 16

1.4.3. NMR spectra for [2]BAr$^F_4$ 18

1.4.4. NMR spectra for [3]OTf 19

1.4.5. NMR spectra for [4]OTf 21

1.4.6. NMR spectra for 5-OTf 22

1.4.7. NMR spectra for [6]OTf 23

1.4.8. NMR spectra for [6]BAr$^F_4$ 24

1.5. NMR-scale experiments 25

1.5.1. NMR investigation into the initial reaction of [6]OTf with Methylamine-Borane 25

1.5.2. Solution-stability investigation of [2]OTf in CD_2Cl_2 25

1.6. Catalysis 27

1.6.1. Dehydropolymerisation of Methylamine-borane with [2]OTf 27

1.6.2. Dehydropolymerisation of methylamine-borane with [6]OTf 30

1.6.3. Dehydropolymerisation of Methylamine-Borane with [6]OTf – Pre-activated catalyst with NMR investigation 34

1.6.4. Dehydropolymerisation of Methylamine-Borane with [6]OTf – Recharge experiment 36

1.6.5. Dehydropolymerisation of Methylamine-Borane with [6]OTf – Mercury drop test for nanoparticles 38

1.6.6. Dehydrocoupling of dimethylamine-borane with [6]OTf - Mercury drop test for nanoparticles 39

1.6.7. Dehydropolymerisation of methylamine-borane with [6]OTf – PMe_3 poisoning test for heterogenous catalysis 40

1.6.8. Dehydropolymerisation of methylamine-borane with [6]OTf – poisoning with Dibenzo(a,e)cyclooctene (DBCOT) to test for homogenous catalysis 41

1.6.9. Dehydropolymerisation of methylamine-borane with [6]OTf – Recharge experiment with filtration, test for homogenous and heterogenous catalysis 42

1.6.10. Large-scale dehydropolymerisation of methylamine-borane with [6]OTf and polymer purification 43

1.6.11. Depolymerisation of poly(N-methylaminoborane) with catalytic NaHMDS... 45

1.6.12. Dehydropolymerisation of Ethylamine-Borane with [6]OTf - Pre-activated catalyst 49

1.6.13. Dehydropolymerisation of *n*-Propylamine-Borane with [6]OTf - Pre-activated catalyst 50

1.7. TEM Rh-nanoparticle images 52

1.8. Computational Calculations using Density Functional Theory (DFT) Methods 57

1.8.1. DFT Optimised *xyz* Coordinates and Collated Energies of 1e,3e,5a-trimethylborazine 57

1.8.2. DFT Optimised *xyz* Coordinates and Collated Energies of 1e,3e,5e-trimethylborazine 60

1. Experimental

1.1. General Procedures

All manipulations, unless otherwise stated, were performed under an argon atmosphere using standard Schlenk line and glove-box techniques. Glassware was oven dried at 130 °C overnight and flamed under vacuum prior to use. CH_2Cl_2 and pentane were dried using a Grubbs-type solvent purification system (MBraun SPS-800) and degassed by three successive freeze-pump-thaw cycles. CD_2Cl_2 and 1,2-$C_6H_4F_2$ (pre-treated with alumina) were dried over CaH_2, vacuum distilled and stored over 3 Å molecular sieves. $H_3B{\cdot}NEtH_2$, $H_3B{\cdot}N^nPrMeH_2$, Na[BArF$_4$], **L1**, [Rh(**L1**)Cl], were prepared by literature methods.[1–3] DABCO, and $H_3B{\cdot}NMe_2H$ were purchased from Aldrich and sublimed before use (5 × 10^{-2} Torr, 298 K). Dibenzo(a,e)cyclooctene was purchased from Tokyo Chemical Industry and used as supplied. $H_3B{\cdot}NMeH_2$ was purchased from Boron Specialities and recrystallized twice from Et_2O at −18 °C. $BH_3{\cdot}$THF (1.0 M in THF), $NEtH_2$ (2.0 M in THF), N^nPrH_2 (neat), and $NMeH_2$ (2.0 M in THF) were purchased from Fisher Scientific and used as received to form solutions in THF solvent of the desired concentrations.

NMR spectra were recorded on a Bruker Avance III 500 MHz NMR spectrometer, a Bruker Avance III HD 600 Ultrashield NMR spectrometer or a Bruker Avance III HD nanobay 400 MHz NMR spectrometer at 298 K. Residual protio solvent was used as reference for ^{1}H spectra in deuterated solvent samples. ^{31}P NMR spectra were externally referenced to 85% H_3PO_4. All chemical shifts (δ) are quoted in ppm and coupling constants (*J*) in Hz.

Mass Spectrometry ESI-MS for purified materials were recorded on a Bruker micrOTOF instrument interfaced with a glove-box in +ve mode using CH_2Cl_2 as a solvent, as detailed previously.[4] The *in-situ* ESI-MS of the dehydropolymerisation of $H_3B{\cdot}NH_2Me$ with **[6]OTf** was recorded by Mr Karl Heaton in the Centre of Excellence in Mass Spectrometry at the University of York. Samples were dissolved in **CH_2Cl_2** and carried in 1:1 MeOH:H_2O.

ICP-MS analyses on were performed at the School of Life Sciences, University of Sussex by Dr Alaa Abdul-Sada. 10 mg samples were dissolved in 10 ml nitric acid (20%). Solutions were diluted by a factor of 10 with dilute hydrochloric acid prior to analysis. Samples were analysed 3 times using an Agilent Technologies X series 2Q ICP-MS. Elemental microanalyses were performed by Stephen Boyer and Orfhlaith McCullough at London Metropolitan University.

Transmission electron microscopy (TEM). Samples were dispersed in THF and cast onto copper grids coated with carbon mesh. The air sensitive samples were prepared in the glovebox. TEM was performed on JEOL 2100 microscope with an accelerating voltage of 200kV.

Gel permeation chromatography (GPC) was performed on a Malvern Viscotek GPCmax chromatograph fitted with a refractive index (RI) detector. The triple-column (plus guard column) setup was contained within an oven (35 °C) and consisted of a porous styrene divinylbenzene copolymer with a maximum pore size of 1.500 Å. THF containing 0.1% w/w $[NBu_4]Br$ was used as the eluent at a flow rate of 1.0 mL min^{-1} . Samples were dissolved in the eluent (2.0 mg mL^{-1}), filtered (0.2 µm pore size) and run immediately. The calibration was conducted using a series of monodisperse polystyrene standards (Mn = 474 – 476,800 g mol^{-1}) obtained from Sigma–Aldrich.

1.2. Syntheses

1.2.1. Synthesis of [1]OTf

To a solution of [Ag(**L1**)(OTf)] (295.4 mg, 0.4 mmol) in dichloromethane (10 ml) was added $[Rh(C_2H_4)_2Cl]_2$ (77.8 mg, 0.2 mmol) and the mixture stirred in the dark for 2 h. The dark brown solution was filtered by cannula to remove AgCl and concentrated under reduced pressure to approx. 1 ml. Pentane (10 ml) was added to precipitate a brown solid which was washed with further pentane and vacuum dried to give the product (283.0 mg, 93%).

^{1}H NMR (400 MHz, CD_2Cl_2) δ 8.43 (t, $^3J_{HH}$ = 8.0 Hz, 1H, Py), 8.11 (d, $^3J_{HH}$ = 8.0 Hz, 2H, Py), 7.35 – 7.19 (m, 6H, Ph), 3.57 (s, 4H, CH_2=CH_2), 3.03 (hept, $^3J_{HH}$ = 6.7 Hz, 4H, CH(^{i}Pr)), 2.09 (s, 6H, Me), 1.28 (d, $^3J_{HH}$ = 6.8 Hz, 12H, CH_3(^{i}Pr)), 1.12 (d, $^3J_{HH}$ = 6.8 Hz, 12H, CH_3(^{i}Pr)). ^{19}F NMR (376 MHz, CD_2Cl_2) δ -78.84 (s, OTf).

HRMS (ESI/QTOF) m/z: [M]+ Calcd for $C_{35}H_{47}N_3Rh$ 212.2820. Found 212.2799. Elem. anal. Calcd for $C_{36}H_{47}N_3F_3O_3S_1Rh$: C, 56.76; H, 6.22; N, 5.52. Found: C, 56.66; H, 5.94; N, 5.48.

Molecular Structure:

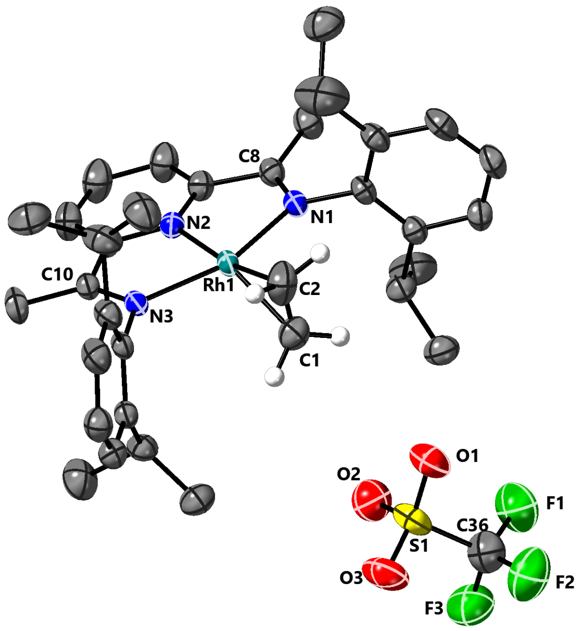

Figure S1. Molecular structure of **[1]OTf** determined by single crystal X-ray diffraction. Hydrogen atoms other than those at the ethene are omitted for clarity. Selected bond lengths [Å] and angles [°]: Rh(1)-N(1) 2.0586(18), Rh(1)-N(2) 1.960(2), Rh(1)-N(3) 2.0578(19), Rh(1)-C(1) 2.174(3), Rh(1)-C(2) 2.165(4), N(1)-C(8) 1.300(3), N(3)-C(10) 1.297(4), N(3)-Rh(1)-N(1) 156.98(8), N(2)-Rh(1)-C(1) 158.81(11), N(2)-Rh(1)-C(2) 165.7 (1).

1.2.2. Synthesis of [2]OTf

To a solution of **[1]OTf** (38.1 mg, 0.05 mmol) in dichloromethane (10 ml) was added $H_3B{\cdot}NMeH_2$ (2.2 mg, 0.05 mmol) and the solution immediately placed under and atmosphere of hydrogen (1 atm H_2 by freeze-thaw pump cycles). The solution as stirred overnight during which time the colour changed from dark brown to dark green. The solution was concentrated under reduced pressure to approx. 1 ml and pentane (10 ml) added to precipitate a dark green solid which was collected and vacuum dried to give the product (30.0 mg, 77%).

^{1}H NMR (400 MHz, CD_2Cl_2) δ 8.28 (t, $^3J_{HH}$ = 8.0 Hz, 1H, Py), 7.86 (d, $^3J_{HH}$ = 8.0 Hz, 2H, Py), 7.41 – 7.26 (m, 6H, Ph), 2.96 (s, 2H, NH_2), 2.90 (hept, $^3J_{HH}$ = 6.7 Hz, 4H, CH(^{i}Pr)), 2.00 (t, $^3J_{HH}$ = 6.0 Hz, 3H, NMe), 1.98 (s, 6H, Me), 1.15 (d, $^3J_{HH}$ = 6.7 Hz, 12H, CH_3(^{i}Pr)), 1.12 (d, $^3J_{HH}$ = 6.7 Hz, 12H, CH_3(^{i}Pr)), -1.98 (s, 3H, BH_3 (in the ^{1}H{^{11}B} NMR spectrum this is observed as a doublet $^1J_{RhH}$ = 19.7 Hz)). ^{11}B NMR (128 MHz, CD_2Cl_2) δ -7.77 (br s, BH_3). ^{19}F NMR (376 MHz, CD_2Cl_2) δ -79.00 (s, OTf).

Elem. anal. Calcd for $C_{35}H_{51}BF_3N_4O_3RhS$: C, 53.99; H, 6.60; N, 7.20. Found: C, 53.59; H, 6.46; N, 7.17.

Molecular structure:

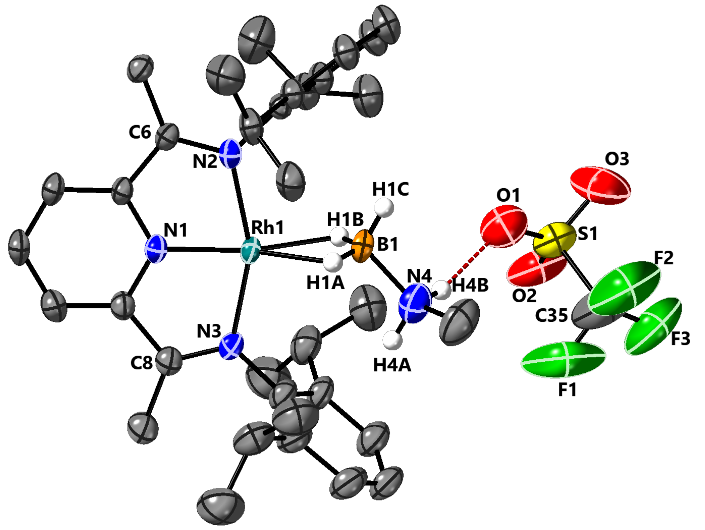

Figure S2. Molecular structure of **[2]OTf** determined by single crystal X-ray diffraction. Hydrogen atoms other than those at the $H_3B{\cdot}NMeH_2$ are omitted for clarity. Selected bond lengths [Å] and angles [°]: Rh(1)-N(1) 1.903(3), Rh(1)-N(2) 2.039(3), Rh(1)-N(3) 2.030(3), Rh(1)-B(1) 2.254(4), Rh(1)-H(1A) 1.89(2), Rh(1)-H(1B) 1.89(2), N(4)-B(1) 1.580(6), N(2)-C(6) 1.315(4), N(3)-C(8) 1.303(5), N(1)-Rh(1)-B(1) 176.73(15), N(1)-Rh(1)-H(1A) 156.1(9), N(1)-Rh(1)-H(1B) 155.5(9), N(3)-Rh(1)-N(2) 157.48(12).

1.2.3. Synthesis of [2]BAr$^F_4$

To a solution of [Rh(**L1**)Cl] (31.0 mg, 0.05 mmol) in dichloromethane (10 ml) was added Na[BAr$^F_4$] (44.3 mg, 0.05 mmol) and $H_3B{\cdot}NMeH_2$ (2.2 mg, 0.05 mmol) and the mixture stirred for 2 h. The dark green solution was filtered by cannula to remove NaCl, concentrated under reduced pressure to approx. 1 ml and pentane (10 ml) added to precipitate a dark green solid which was washed with pentane and vacuum dried to give the product (46.0 mg, 62%).

^{1}H NMR (400 MHz, CD_2Cl_2) δ 8.26 (t, $^3J_{HH}$ = 8.0 Hz, 1H, Py), 7.84 (d, $^3J_{HH}$ = 8.0 Hz, 2H, Py), 7.73 (s, 8H, BAr$^F_4$), 7.56 (s, 4H, BAr$^F_4$), 7.37 – 7.24 (m, 6H, Ph), 2.93 (hept, $^3J_{HH}$ = 6.9 Hz, 4H, CH(^{i}Pr)), 2.23 (s, 2H, NH_2), 2.05 (t, $^3J_{HH}$ = 6.1 Hz, 3H, NMe), 1.98 (s, 6H, Me), 1.13 (d, $^3J_{HH}$ = 6.9 Hz, 24H, CH_3(^{i}Pr)), -1.87 (s, 3H, BH_3 (in the ^{1}H{^{11}B} NMR spectrum this is observed as a doublet $^1J_{RhH}$ = 18.3 Hz)). ^{11}B NMR (128 MHz, CD_2Cl_2) δ -6.62 (BAr$^F_4$), -9.72 (br s, BH_3).

HRMS (ESI/QTOF) m/z: [M]+ Calcd for $C_{34}H_{51}BN_4Rh$ 629.3262; Found 629.3262.

Elem. anal. Calcd for $C_{66}H_{63}B_2F_{24}N_4Rh$: C, 53.11; H, 4.25; N, 3.75. Found: C, 52.96; H, 4.15; N, 3.73.

Molecular structure:

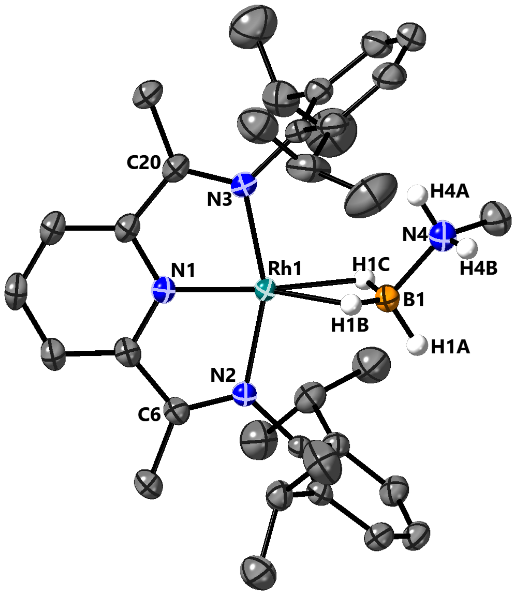

Figure S3. Molecular structure of **[2]BAr$^F_4$** determined by single crystal X-ray diffraction. Hydrogen atoms other than those at the $H_3B{\cdot}NMeH_2$ are omitted for clarity. Selected bond lengths [Å] and angles [°]: Rh(1)-N(1) 1.9002(15), Rh(1)-N(2) 2.0402(15), Rh(1)-N(3) 2.0353(15), Rh(1)-B(1) 2.277(3), Rh(1)-H(1B) 1.90(3), Rh(1)-H(1C) 2.04(3), N(2)-C(6) 1.305(2), N(3)-C(20) 1.308(2), N(4)-B(1) 1.596(4), N(3)-Rh(1)-N(2) 157.55(7), N(1)-Rh(1)-B(1) 174.82(10), N(1)-Rh(1)-H(1B) 146.5(10), N(1)-Rh(1)-H(1C) 155.3(10).

1.2.4. Synthesis of [3]OTf

To a solution of **[1]OTf** (19.0 mg, 0.025 mmol) in dichloromethane (5 ml) was added to $H_3B{\cdot}NEtH_2$ (1.5 mg, 0.025 mmol) and the solution immediately placed under an atmosphere of hydrogen (4 atm H_2 by freeze-pump-thaw cycles). The dark green solution was stirred overnight. The solution was then concentrated under reduced pressure to approx. 0.5 ml and layered with pentane (10 ml) to precipitate a dark green crystalline solid. This solid was dried in-vacuo to give the product (8 mg, 39%).

^{1}H NMR (600 MHz, CD_2Cl_2) δ 8.28 (t, $^3J_{HH}$ = 8.0 Hz, 1H, Py), 7.87 (d, $^3J_{HH}$ = 8.0 Hz, 2H, Py), 7.40 – 7.25 (m, 6H, Ph), 2.92 (s, br, 2H, NH_2), 2.91 (hept, $^3J_{HH}$ = 6.8 Hz, 4H, CH(^{i}Pr)), 2.30 (m, 2H, CH_2(NEt)), 1.98 (s, 6H, Me), 1.14 (d, $^3J_{HH}$ = 6.8 Hz, 12H, CH_3(^{i}Pr)), 1.12 (d, $^3J_{HH}$ = 6.8 Hz, 12H, CH_3(^{i}Pr)), 0.85 (t, $^3J_{HH}$ = 7.5 Hz, 3H, CH_3(NEt)), -1.97 (s, br, 3H, BH_3). ^{11}B NMR (192 MHz, CD_2Cl_2) δ -8.64 (s, br, BH_3). Elem. Anal. Calcd for $C_{36}H_{53}BF_3N_4O_3RhS \cdot (C_5H_{12})_{0.5}$: C, 55.80; H, 7.18; N, 6.76. Found: C, 55.88; H, 6.78; N, 6.74.

Molecular structure:

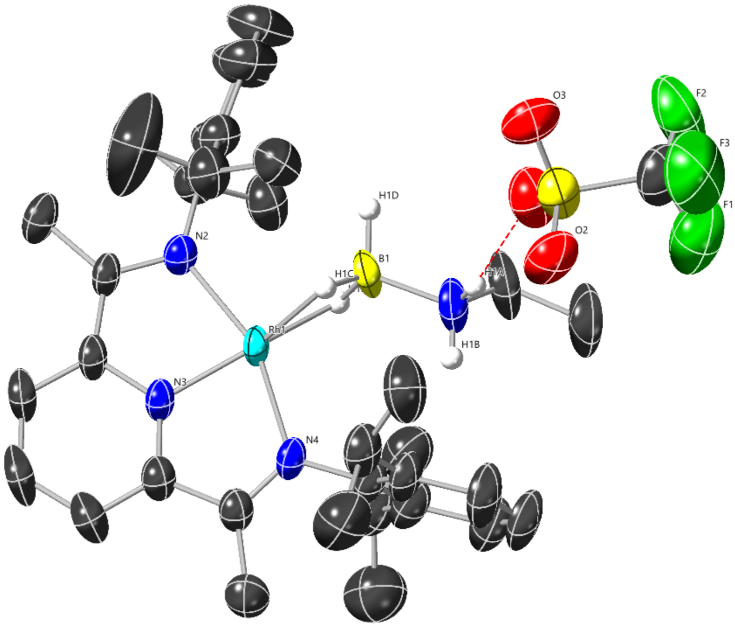

Figure S4. Molecular structure of **[3]OTf** determined by single crystal X-ray diffraction. Solved from a twinned crystal. Hydrogen atoms are omitted for clarity. Selected bond lengths [Å] and angles [°]: Rh(1)-N(2) 2.051(5), Rh(1)-N(3) 1.900(5), Rh(1)-N(4) 2.041(5), Rh(1)-H(1C) 1.89(12), Rh(1)-H(1E) 1.89(7), N(2)-C(15) 1.324(9), N(4)-C(22) 1.297(10), N(1)-B(1) 1.581(12), N(2)-Rh(1)-N(4) 157.7(2), N(4)-Rh(1)-H(1C) 145(3), N(4)-Rh(1)-H(1E) 147.0(19).

1.2.5. Synthesis of [4]OTf

To a solution of **[1]OTf** (19.0 mg, 0.025 mmol) in dichloromethane (5 ml) was added to $H_3B{\cdot}N^nPrH_2$ (2 mg, 0.025 mmol) and the solution immediately placed under an atmosphere of hydrogen (4 atm H_2 by freeze-pump-thaw cycles). The dark blue-green solution was stirred

overnight. The solution was then concentrated under reduced pressure to approx. 0.5 ml and layered with pentane (10 ml) to precipitate a dark green crystalline solid. This solid was dried in-vacuo to give the product (12 mg, 57%).

^{1}H NMR (500 MHz, CD_2Cl_2) δ 8.29 (t, $^3J_{HH}$ = 8.0 Hz, 1H, Py), 7.88 (d, $^3J_{HH}$ = 8.0 Hz, 2H, Py), 7.40 – 7.24 (m, 6H, Ph), 2.91 (hept, $^3J_{HH}$ = 6.8 Hz, 4H, CH(^{i}Pr)), 2.89 (s, br, 2H, NH_2), 2.19 (m, 2H, CH_2(N^{n}Pr)), 1.98 (s, 6H, Me), 1.18 (m, 2H, CH_2(N^{n}Pr), 1.14 (d, $^3J_{HH}$ = 7.0 Hz, 12H, CH_3(^{i}Pr)), 1.12 (d, $^3J_{HH}$ = 7.0 Hz, 12H, CH_3(^{i}Pr)), 0.72 (t, $^3J_{HH}$ = 7.3 Hz, 3H, CH_3(N^{n}Pr)), -1.95 (s, br, 3H, BH_3). ^{11}B NMR (160 MHz, CD_2Cl_2) δ -8.53 (s, br, BH_3). Elem. Anal. Calcd for $C_{37}H_{55}BF_3N_4O_3RhS$: C, 55.09; H, 6.80; N, 6.84. Found: C, 55.26; H, 6.61; N, 6.89.

Molecular structure:

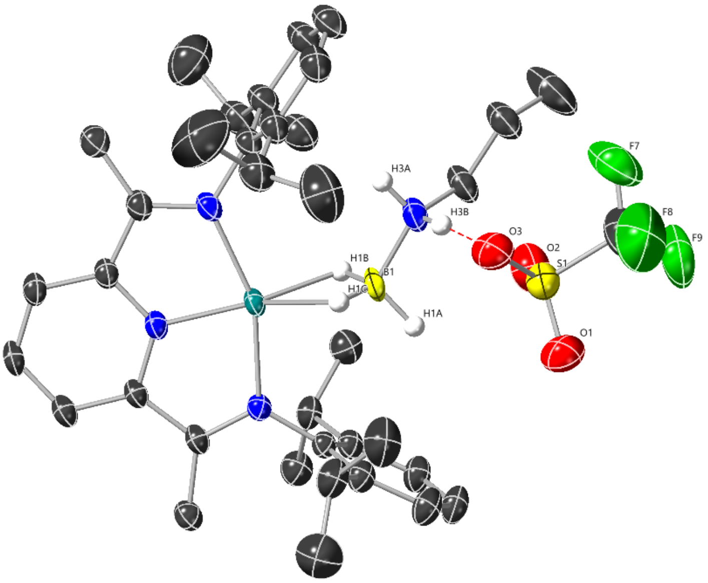

Figure S5. Molecular structure of **[4]OTf** determined by single crystal X-ray diffraction. Hydrogen atoms are omitted for clarity. Selected bond lengths [Å] and angles [°]: Rh(1)-N(1) 2.0432(18), Rh(1)-N(2) 2.0321(18), Rh(1)-N(20) 1.903(3), Rh(1)-H(1B) 1.90(5), Rh(1)-H(1C) 1.89(4), N(1)-C(13) 1.316(4), N(2)-C(21) 1.316(5), N(3)-B(1) 1.584(5), N(1)-Rh(1)-N(2) 157.43(11), N(20)-Rh(1)-H(1B) 151.5(12), N(20)-Rh(1)-H(1C) 148.2(13).

1.2.6. Synthesis of 5-OTf

Complex **5-OTf** was highly unstable but could be observed by NMR spectroscopy in the following reaction.

In a high-pressure NMR tube a solution of **[1]OTf** (~10 mg) in CD_2Cl_2 (0.4 ml) was hydrogenated (1 atm H_2 by freeze-thaw pump cycles). The solution was stirred for 24 h (NMR tube spinner) and NMR spectrum measured after this time showing the formation of ethane and the product. Crystals of suitable quality to be measured by single crystal X-ray diffraction were grown in the NMR tube under an atmosphere of hydrogen (1 atm).

^{1}H NMR (400 MHz, CD_2Cl_2) δ 8.43 (t, $^{3}J_{HH}$ = 8.0 Hz, 1H, Py), 7.61 (d, $^{3}J_{HH}$ = 8.0 Hz, 2H, Py), 7.30 – 7.21 (m, 6H, Ph), 3.15 (hept, $^{3}J_{HH}$ = 6.7 Hz, 4H, CH(^{i}Pr)), 1.81 (s, 6H, Me), 1.15 (d, $^{3}J_{HH}$ = 6.8 Hz, 24H, CH_3(^{i}Pr)). ^{19}F NMR (376 MHz, CD_2Cl_2) δ -79.10 (s, OTf).

Molecular structure:

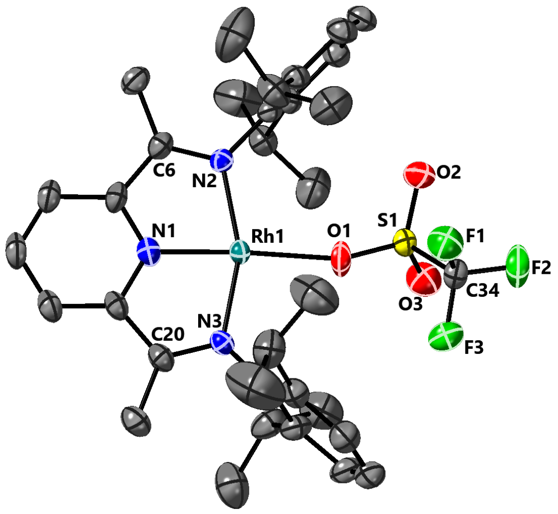

Figure S6. Molecular structure of **5-OTf** determined by single crystal X-ray diffraction. Hydrogen atoms are omitted for clarity. Selected bond lengths [Å] and angles [°]: Rh(1)-O(1) 2.103(2), Rh(1)-N(1) 1.888(2), Rh(1)-N(2) 2.033(2), Rh(1)-N(3) 2.029(2), N(2)-C(6) 1.307(4), N(3)-C(20) 1.305(4), N(1)-Rh(1)-O(1) 175.07(11), N(3)-Rh(1)-N(2) 158.92(10).

1.2.7. Synthesis of [6]OTf

To a solution of **[1]OTf** (38.1 mg, 0.05 mmol) in dichloromethane (10 ml) was added NH_2Me (0.25 ml, 0.2 M solution in THF, 0.05 mmol) and the mixture stirred for 2 h. The solution was concentrated under reduced pressure to approx. 1 ml and pentane (10 ml) added to precipitate a dark brown solid which was washed with pentane and vacuum dried to give the product (25.1 mg, 66%).

^{1}H NMR (400 MHz, CD_2Cl_2) δ 8.43 (t, $^{3}J_{HH}$ = 8.0 Hz, 1H, Py), 7.86 (d, $^{3}J_{HH}$ = 7.8 Hz, 2H, Py), 7.42 – 7.24 (m, 6H, Ph), 3.14 (hept, $^{3}J_{HH}$ = 6.7 Hz, 4H, CH(^{i}Pr)), 1.98 (s, 6H, Me), 1.86 (s, 2H, NH_2), 1.34 (t, $^{3}J_{HH}$ = 6.8 Hz, 3H, Me), 1.19 (d, $^{3}J_{HH}$ = 6.7 Hz, 12H, CH_3(^{i}Pr)), 1.11 (d, $^{3}J_{HH}$ = 6.7 Hz, 12H, CH_3(^{i}Pr)). ^{19}F NMR (376 MHz, CD_2Cl_2) δ -78.93 (s, OTf).

Elem. anal. Calcd for $C_{35}H_{48}F_3N_4O_3RhS$: C, 54.97; H, 6.33; N, 7.33. Found: C, 54.72; H, 6.18; N, 7.31.

Molecular Structure:

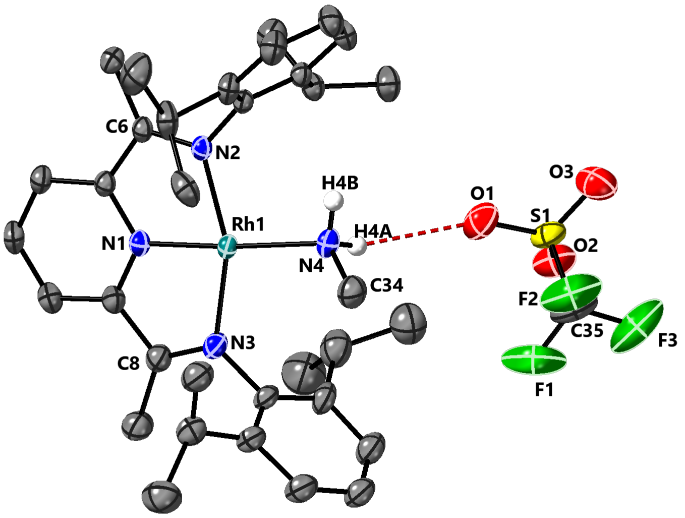

Figure S7. Molecular structure of **[6]OTf** determined by single crystal X-ray diffraction. Hydrogen atoms other than those at the $NMeH_2$ are omitted for clarity. Selected bond lengths [Å] and angles [°]: Rh(1)-N(1) 1.9084(17), Rh(1)-N(2) 2.0372(18), Rh(1)-N(3) 2.0537(19), Rh(1)-N(4) 2.1342(18), N(2)-C(6) 1.310(3), N(3)-C(8) 1.309(3), N(1)-Rh(1)-N(4) 175.13(8), N(2)-Rh(1)-N(3) 157.81(7).

1.2.8. Synthesis of [6]BAr$^F{}_4$

To a solution of **1** (31.0 mg, 0.05 mmol) in dichloromethane (10 ml) was added Na[BAr$^F{}_4$] (44.3 mg, 0.05 mmol) and NH_2Me (0.25 ml, 0.2 M solution in THF, 0.05 mmol) and the mixture stirred for 2 h. The dark green/brown solution was filtered by cannula to remove NaCl, concentrated under reduced pressure to approx. 1 ml and pentane (10 ml) added to precipitate a green/brown solid which was washed with pentane and vacuum dried to give the product (35.5 mg, 48%).

^{1}H NMR (400 MHz, CD_2Cl_2) δ 8.33 (t, $^3J_{HH}$ = 8.0 Hz, 1H, Py), 7.76 (d, $^3J_{HH}$ = 8.0 Hz, 2H, Py), 7.72 (s, 8H, BAr$^F{}_4$), 7.56 (s, 4H, BAr$^F{}_4$), 7.44 – 7.20 (m, 6H, Ph), 3.11 (hept, $^3J_{HH}$ = 6.8 Hz, 4H, CH(^{i}Pr)), 1.96 (s, 6H, Me), 1.87 (s, 2H, NH_2), 1.34 (t, $^3J_{HH}$ = 6.7 Hz, 3H, NMe), 1.18 (d, $^3J_{HH}$ = 6.8 Hz, 12H, CH_3(^{i}Pr)), 1.10 (d, $^3J_{HH}$ = 6.8 Hz, 12H, CH_3(^{i}Pr)). ^{11}B NMR (128 MHz, CD_2Cl_2) δ -6.62 (s, BAr$^F{}_4$). ^{19}F NMR (376 MHz, CD_2Cl_2) δ -62.90 (s, BAr$^F{}_4$).

HRMS (ESI/QTOF) m/z: [M]+ Calcd for $C_{34}H_{48}N_4Rh$ 615.2929; Found 615.2937.

Elem. anal. Calcd for $C_{66}H_{60}BF_{24}N_4Rh$: C, 53.60; H, 4.08; N, 3.79. Found: C, 53.65; H, 4.08; N, 3.63.

Molecular Structure:

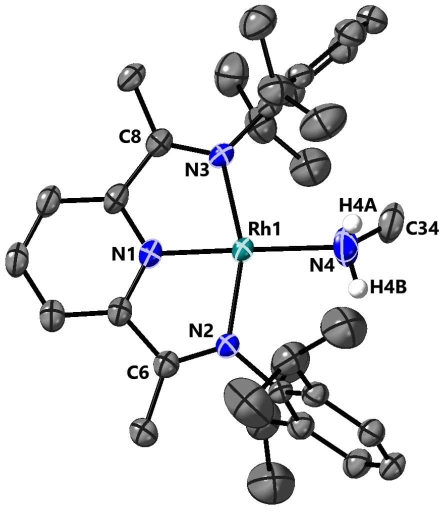

Figure S8. Molecular structure of **[6]BArF$_4$** determined by single crystal X-ray diffraction. Hydrogen atoms other than those at the NMeH2 are omitted for clarity. Selected bond lengths [Å] and angles [°]: Rh(1)-N(1) 1.890(18), Rh(1)-N(2) 2.0196(17), Rh(1)-N(3) 2.0372(18), Rh(1)-N(4) 2.124(3), N(2)-C(6) 1.309(3), N(3)-C(8) 1.305(3), N(1)-Rh(1)-N(4) 175.64(10), N(2)-Rh(1)-N(3) 158.43(8).

1.3. Crystallography

Single-crystal X-Ray diffraction data were collected (ω-scans) on either an Oxford Diffraction/Agilent SuperNova diffractometer (Cu-Kα radiation, λ = 1.54184 Å) at the Oxford Chemical Crystallography Service at the University of Oxford, a Rigaku SuperNova diffractometer (Cu-Kα radiation, λ = 1.54184 Å) at the University of York, or at the UK National Crystallography Service from the University of Southampton, using a Rigaku 007HF diffractometer equipped with Varimax confocal mirrors (Cu-Kα radiation, λ = 1.54184 Å), an AFC11 goniometer and a HyPix 6000 detector. All diffractometers were equipped with nitrogen gas Oxford Cryostreams Cryostream units.[5] Diffraction images from raw frame data were reduced using the CryAlisPro suite of programmes. The structures were solved using SHELXT[6] and refined by full convergence on F^2 against all independent reflections by full-matrix least-squares using SHELXL[7] (version 2018/3) through the Olex2 GUI.[8] All non-hydrogen atoms were refined anisotropically and hydrogen atoms were geometrically placed and allowed to ride on their parent atoms, unless otherwise stated (see specific details for each molecular structure in the text and supplementary CIF information). CF_3 groups on the $BAr^F{}_4{}^-$ and $^-$OTf anions were necessarily modelled as disordered over two main domains and restrained to maintain sensible geometries. Disordered groups and solvent molecules were restrained to maintain sensible chemical geometries. Selected crystallographic data are summarised in **Table S1** & **S2** and full details are given in the supplementary deposited CIF files (CCDC 1993413-15, 1993419 & 2271696-2271699). These data can be obtained free of charge from the Cambridge Crystallographic Data Centre.

1.3.1. Additional crystallographic details

Compound **[1]OTf** crystallised with 0.5 molecules of *n*-pentane within the asymmetric unit. The chemical occupancies were constrained to an approximate value of 0.5, and geometries restrained with DFIX, SIMU and RIGU restraints to a sensible chemical geometry. The $^-$OTf anion was also modelled over two main domains, and geometries restrained with SADI, SIMU and RIGU restraints to sensible chemical geometries.

Compound **[2]OTf** crystallised with 0.5 molecules of 1,2-$F_2C_6H_4$ within the asymmetric unit which was restrained with SADI, RIGU and ISOR restraints to a sensible chemical geometry. The $MeH_2N{\cdot}BH_3$ unit was found to be disordered; however, it could not be satisfactorily modelled. The H-atoms on the BH_3 unit were located in the Fourier difference map and restrained with SADI restraints to sensible Rh-H-B distances. One of the 2,6-di-iso-propylphenyl groups were disordered over two positions and restrained with SADI, SIMU and RIGU restraints to sensible chemical geometries.

Compound **[2]BAr$^F_4$** crystallised with one molecule of *n*-pentane within the asymmetric unit which was modelled as two disordered components, each constrained to chemical occupancies of 0.5. The disordered *n*-pentane was restrained with DFIX, DANG, SIMU and RIGU restraints to a sensible chemical geometry. The $MeH_2N{\cdot}BH_3$ unit was modelled as disordered over two main domains with 0.86:14 refined chemical occupancies. B-N, Rh-B and C-N bond lengths were restrained to refine to similar metrics on both disordered components. The H atoms on the BH_3 group were located in the difference Fourier difference map for the major component (i.e. 86% occupancy) and allowed to ride on the parent B atom, whereas they were placed at expected positions for the minor component.

Compound **[3]OTf** crystallised with 0.5 molecules of *n*-pentane within the asymmetric unit. The chemical occupancies were constrained to an approximate value of 0.5, and the geometries were restrained with EADP, RIGU, SADI, DFIX and DANG restraints to a sensible chemical geometry. The H-atoms on the BH_3 group were located in the Fourier difference map and freely refined.

Compound **[4]OTf** crystallised with two crystallographically independent units of [Rh(**L1**)($H_2BHN(^nPr)H_2$)][OTf] per asymmetric unit. Within the asymmetric unit was located CH_2Cl_2 which was freely refined, then constrained at 14.2 % occupancy. One of the $^-$OTf anions in the asymmetric unit was modelled over two main domains and the geometries restrained with SADI restraints to a sensible chemical geometry. The H-atoms on the BH_3 groups were found in the Fourier difference map and freely refined.

Compound **5-OTf** crystallised with 1.5 molecules of 1,2-$F_2C_6H_4$ within the asymmetric unit. The geometries were restrained with DFIX, SADI, DANG, FLAT, DELU, SIMU and RIGU restraints to sensible chemical geometries.

Compound **[6]OTf** crystallised with one molecule of 1,2-$F_2C_6H_4$ within the asymmetric unit with the geometry restrained using SADI, FLAT, SIMU and RIGU restraints to reasonable chemical geometries. One of the 2,6-di-iso-propylphenyl groups were disordered over two positions and restrained with SADI, SIMU and RIGU restraints to sensible chemical geometries.

Compound **[6]BAr$^F_4$** crystallised with one molecule of *n*-pentane within the asymmetric unit which could not be satisfactorily modelled, and was treated with the Olex2 implementation of the BYPASS solvent mask.[9] Disordered *iso*-propyl methyls on the 2,6-di-*iso*-propylphenyl groups were modelled over two sites and restrained with SADI, SIMU and RIGU restraints to sensible chemical geometries.

1.3.2. Crystallographic tables

Table S1

	**[1]OTf**	**[2]OTf**	**[2]BAr$^F_4$**	**[3]OTf**
CCDC Number	22711697	1993415	1993414	2271698
Formula	$C_{38.5}H_{53}F_3N_3O_3RhS$	$C_{38}H_{53}BF_4N_4O_3RhS$	$C_{71}H_{75}B_2F_{24}N_4Rh$	$C_{38.5}H_{59}BF_3N_4O_3RhS$
M_w	797.81	835.62	1564.88	828.67
Crystal System	Triclinic	monoclinic	monoclinic	Monoclinic
Space Group	*P*-1	C21/c	P21/c	*C*2/c
T / K	150.15	150(1)	150(1)	110.00(10)
a / Å	10.3593(3)	31.9289(5)	17.51072(11)	31.377(5)
b / Å	12.6796(3)	13.9251(2)	18.22696(9)	14.514(2)
c / Å	16.3022(4)	20.3414(3)	24.15311(13)	19.769(4)
α / °	92.647(2)	90	90	90
β / °	102.000(2)	106.046(2)	107.7367(6)	105.514(18)
γ / °	92.877(2)	90	90	90
V / Å^3	2088.37(9)	8691.7(2)	7342.45(8)	8675(3)
Z	2	8	4	8
ρ_{calc} / g cm^{-3}	1.269	1.277	1.416	1.269
μ / mm^{-1}	4.184	4.083	2.803	4.050
2Θ range / °	5.552 to 153.386	2.880 to 76.590	3.592 to 76.221	7.742 to 158.332
Reflns collected	35651	40316	101603	38641
R_{int}	0.0327	0.0394	0.0319	0.0626
Completeness / %	99.8	99.9 %	100.0 %	99.6
Data/restr/param	8667/419/554	9069 / 814 / 638	15299 / 902 / 1167	8886/29/506
R_1 [I > 2σ(I)]	0.0355	0.0582	0.0363	0.0888
wR_2 [all data]	0.1030	0.1775	0.0974	0.2153
GooF	1.071	1.069	1.030	1.042
Largest pk/hole / eÅ^{-3}	1.00 / -0.42	1.831 and -0.992	0.620 and -0.445	2.31 / -1.34

Table S2

	[4]OTf	5-OTf	[6]OTf	[6]BAr$^F_4$
CCDC Number	2271699	1993413	1993419	2271696
Formula	$C_{37.07}H_{55.14}BCl_{0.14}F_3N_4O_3RhS$	$C_{43}H_{49}F_6N_3O_3RhS$	$C_{41}H_{52}F_5N_4O_3RhS$	$C_{68.5}H_{66}BF_{24}N_4Rh$
M_w	812.66	904.82	878.83	1514.97
Crystal System	Triclinic	triclinic	monoclinic	Monoclinic
Space Group	*P*-1	P–1	C2/c	*P*2$_1$/n
T / K	110.15	150(1)	150(1)	150.15
a / Å	14.4178(2)	10.9023(3)	31.5501(3)	11.98940(10)
b / Å	17.0136(3)	14.2433(5)	13.46510(10)	17.42840(10)
c / Å	19.9412(3)	15.9876(5)	20.6980(2)	34.0961(2)
α / °	73.1840(10)	105.922(3)	90	90
β / °	88.6930(10)	105.546(3)	106.6940(10)	91.8660(10)
γ / °	66.8010(10)	106.987(3)	90	90
V / Å^3	4280.62(12)	2113.30(13)	8422.43(14)	7120.81(8)
Z	4	2	8	4
ρ_{calc} / g cm^{-3}	1.261	1.422	1.386	1.413
μ / mm^{-1}	4.174	4.314	4.282	2.875
2Θ range / °	6.928 to 154.044	3.106 to 76.482	4.002 to 76.282	5.696 to 153.504
Reflns collected	49895	17316	50354	142185
R_{int}	0.0334	0.0426	0.0269	0.0413
Completeness / %	99.9	100.0 %	100%	99.9
Data/restr/param	17384/19/1048	8758 / 604 / 633	8737 / 1296 / 693	14924/534/1006
R_1 [I > 2σ(I)]	0.0375	0.0412	0.0325	0.0409
wR_2 [all data]	0.0940	0.1096	0.0834	0.1087
GooF	1.043	1.034	1.027	1.029
Largest pk/hole / eÅ^{-3}	0.69 / -0.94	1.009 and -0.599	0.714 and -0.740	1.20 / -0.84

1.4. NMR spectra

1.4.1. NMR spectra for [1]OTf

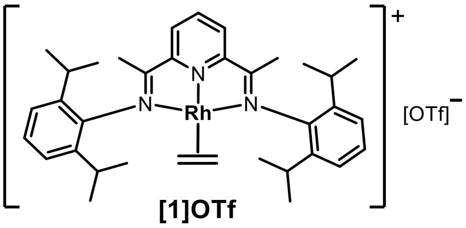

[1]OTf

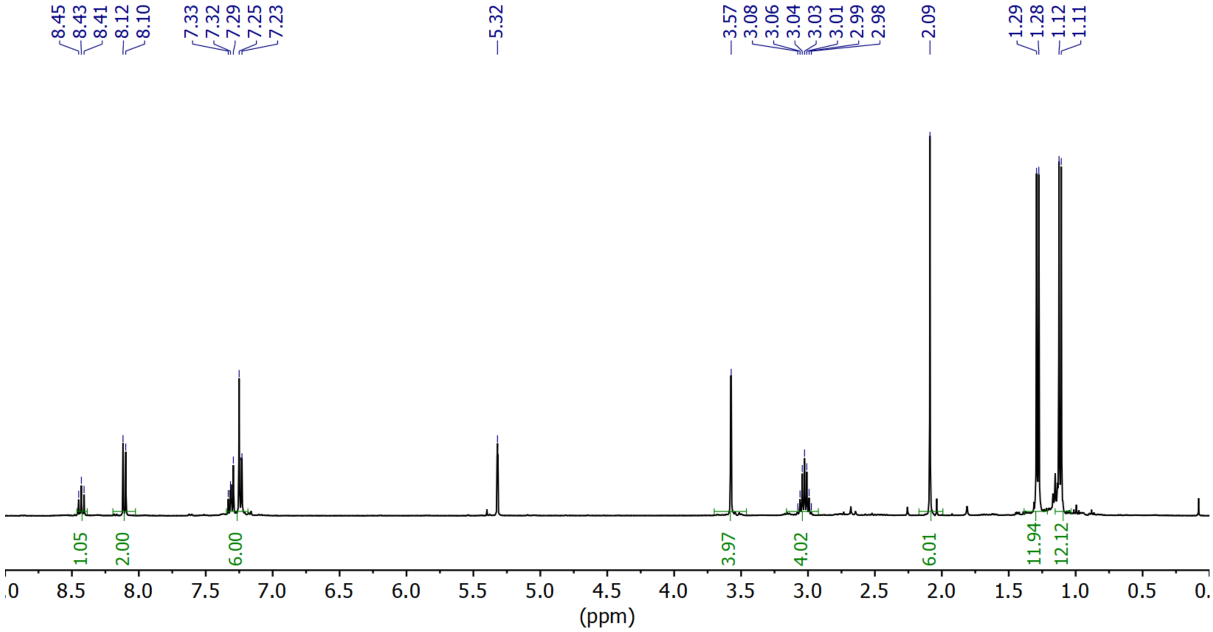

Figure S9. ^{1}H NMR of **[1]OTf** (400 MHz, CD_2Cl_2)

1.4.2. NMR spectra for [2]OTf

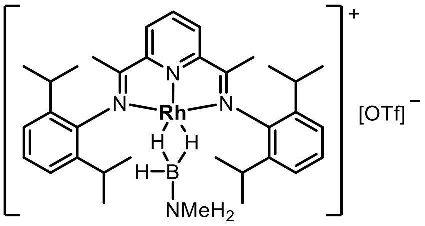

[2]OTf

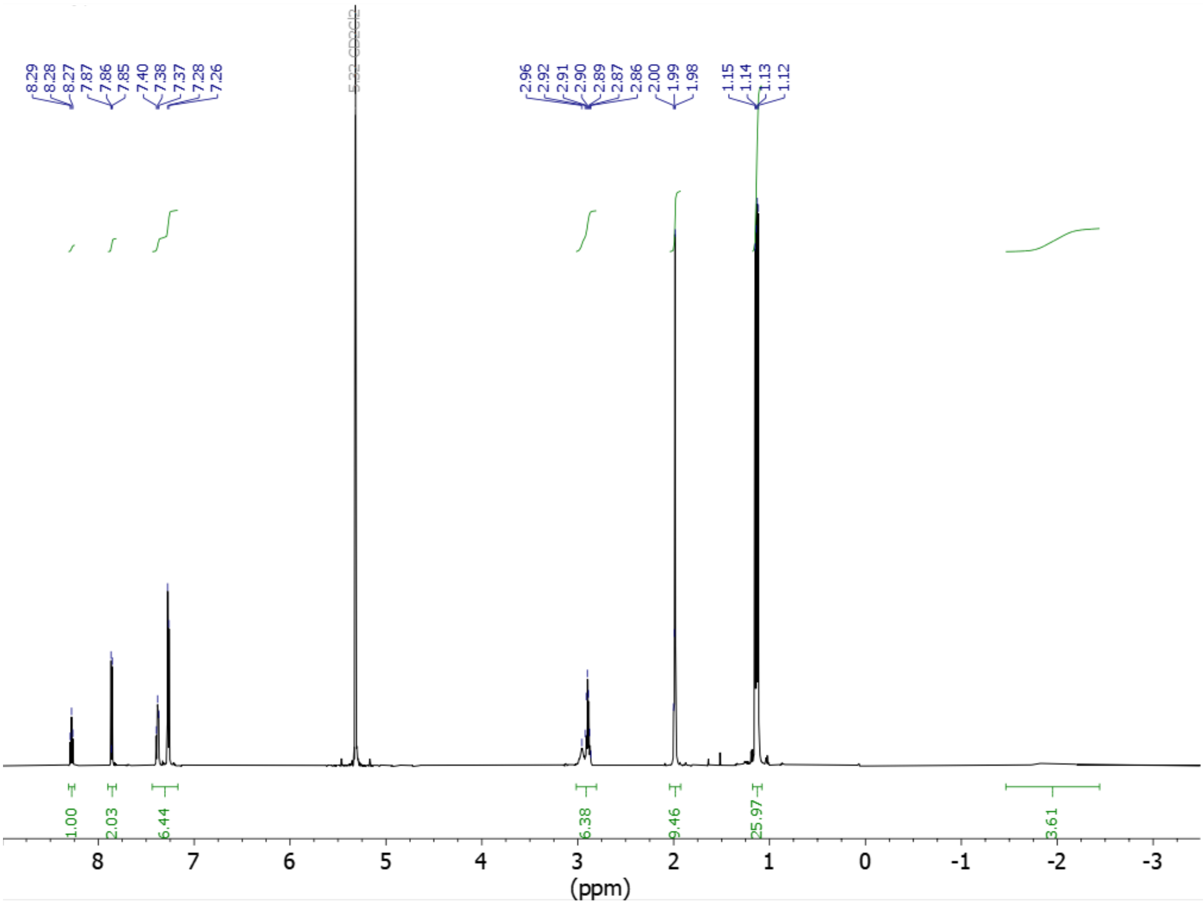

Figure S10. 1H NMR of **[2]OTf** (400 MHz, CD2Cl2)

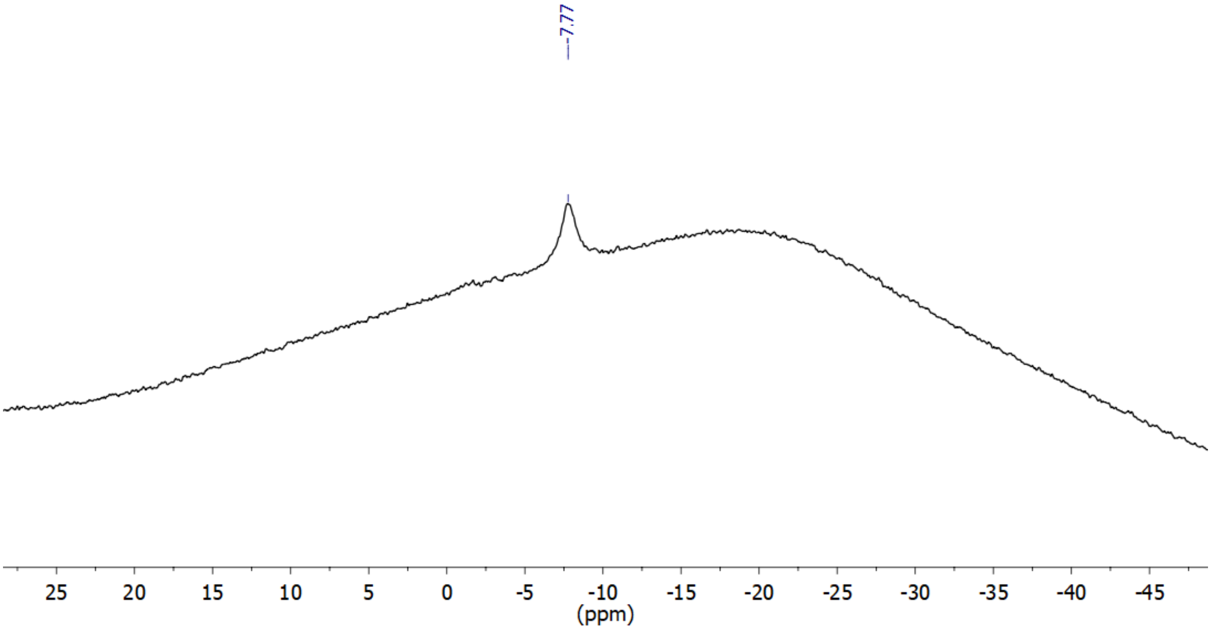

Figure S11. ^{11}B NMR of **[2]OTf** (128 MHz, CD_2Cl_2)

1.4.3. NMR spectra for [2]BAr$^F_4$

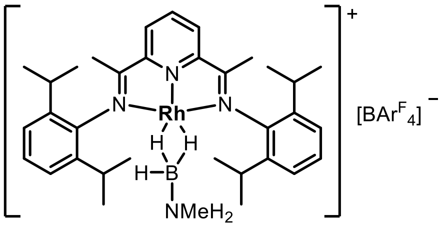

[2]BAr$^F_4$

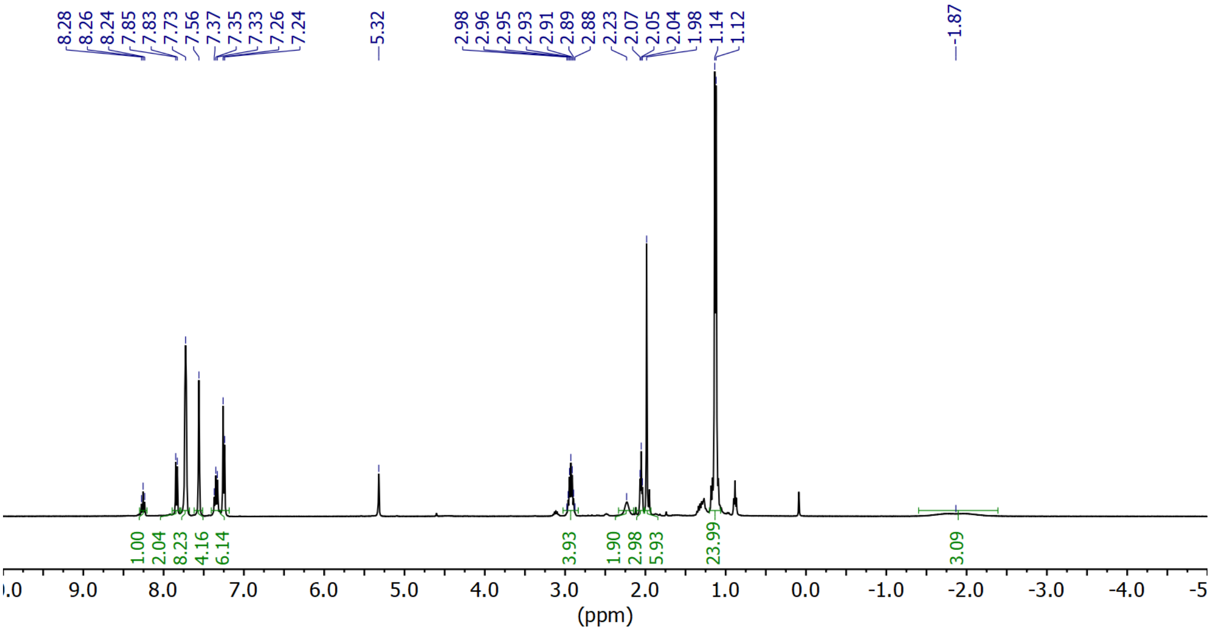

Figure S12. ^{1}H NMR of **[2]BAr$^F_4$** (400 MHz, CD_2Cl_2)

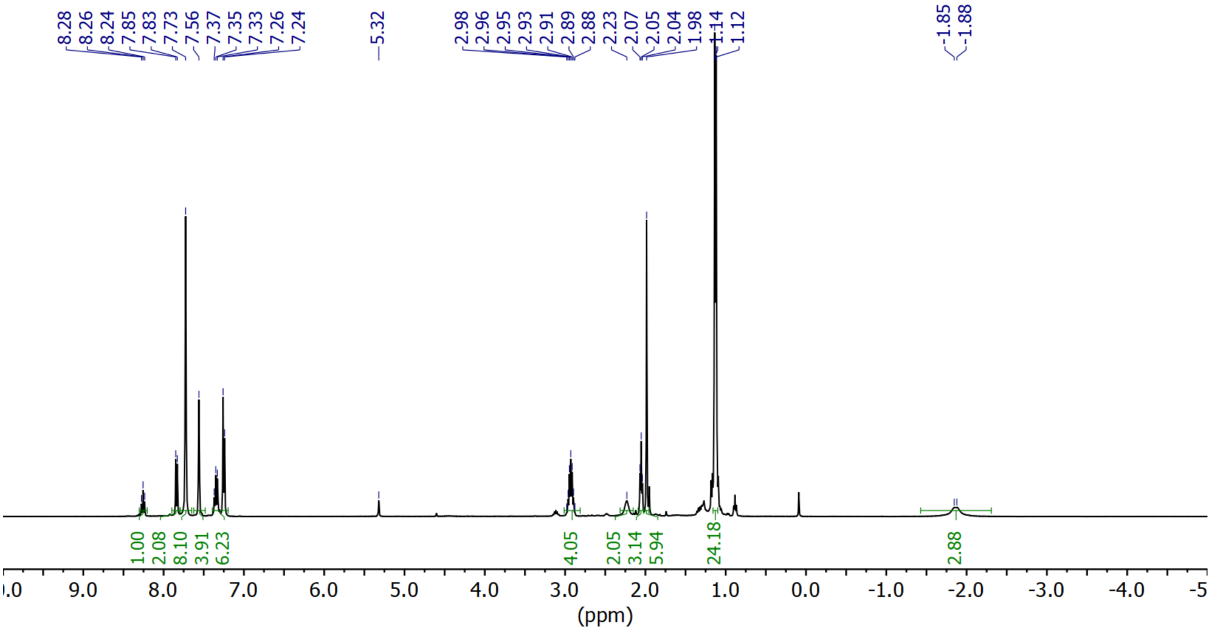

Figure S13. ^{1}H{^{11}B} NMR of **[2]BAr$^F_4$** (400 MHz, CD_2Cl_2)

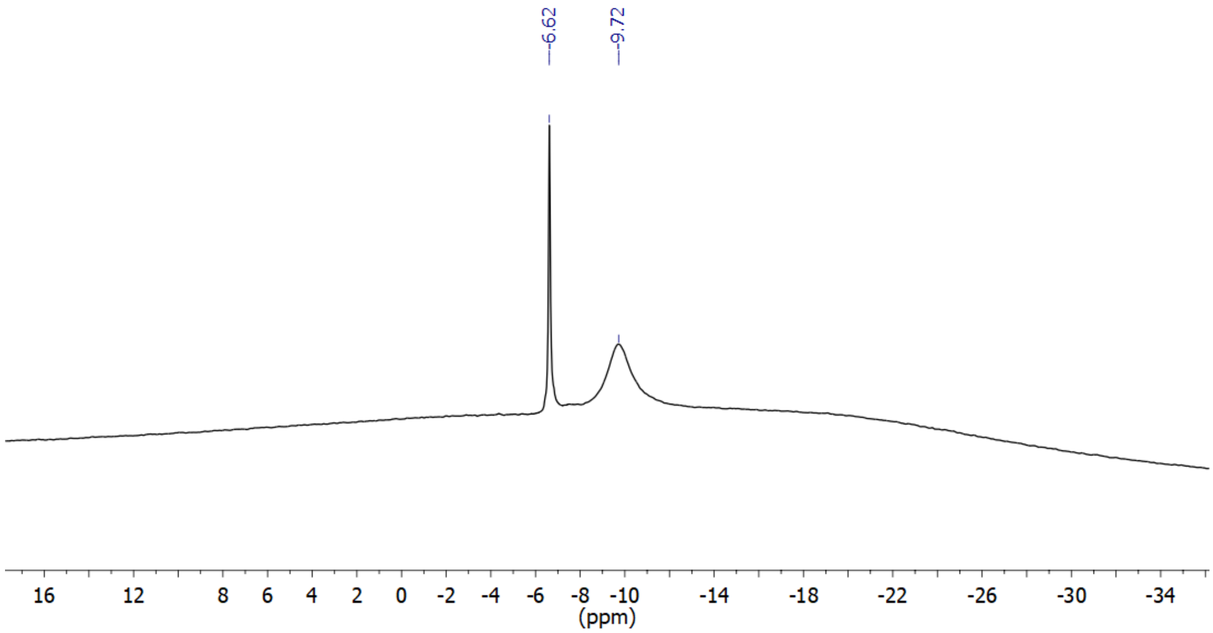

Figure S14. ^{11}B NMR of **[2]BAr^{F}$_4$** (128 MHz, CD_2Cl_2)

1.4.4. NMR spectra for [3]OTf

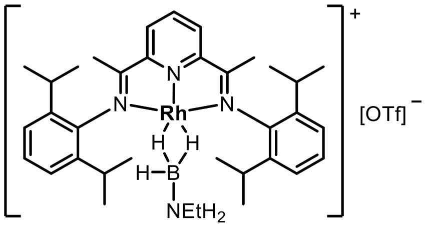

[3]OTf

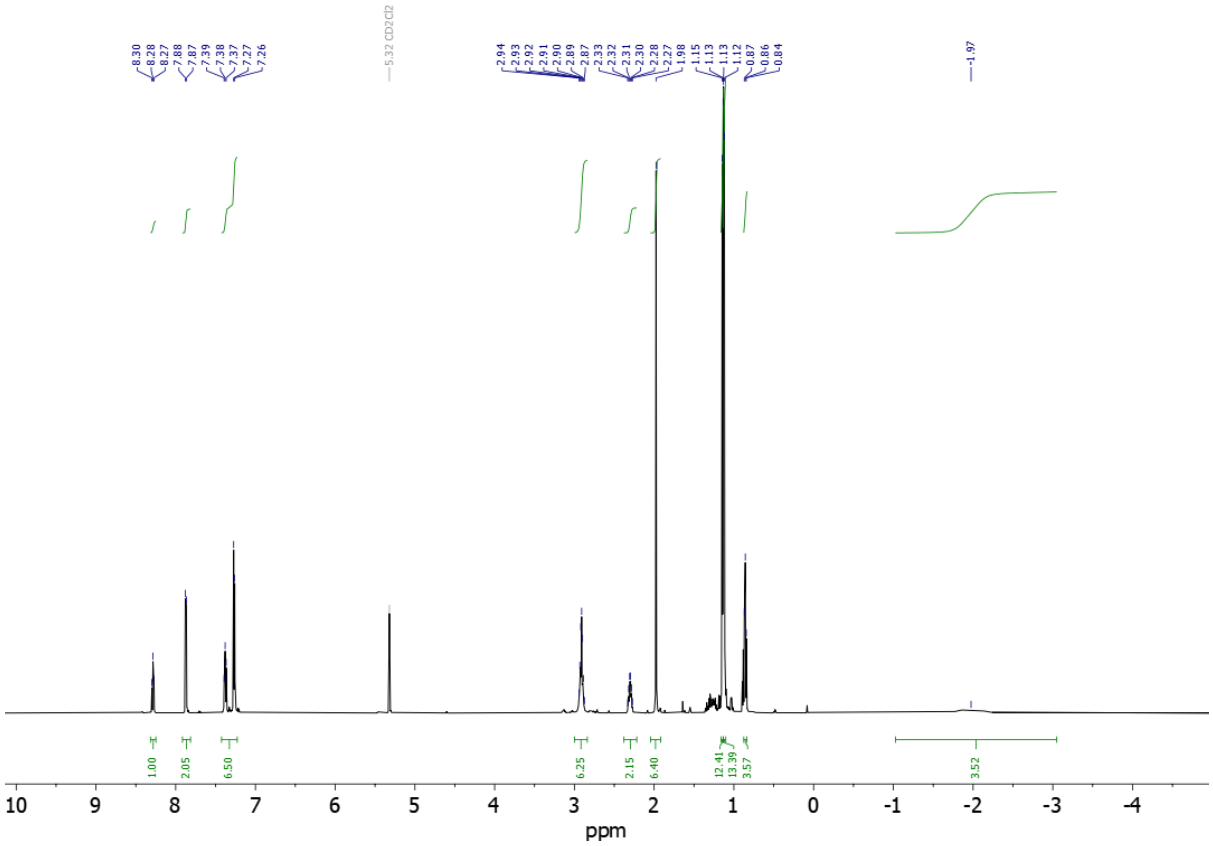

Figure S15. ^{1}H NMR of **[3]OTf** (600 MHz, CD_2Cl_2)

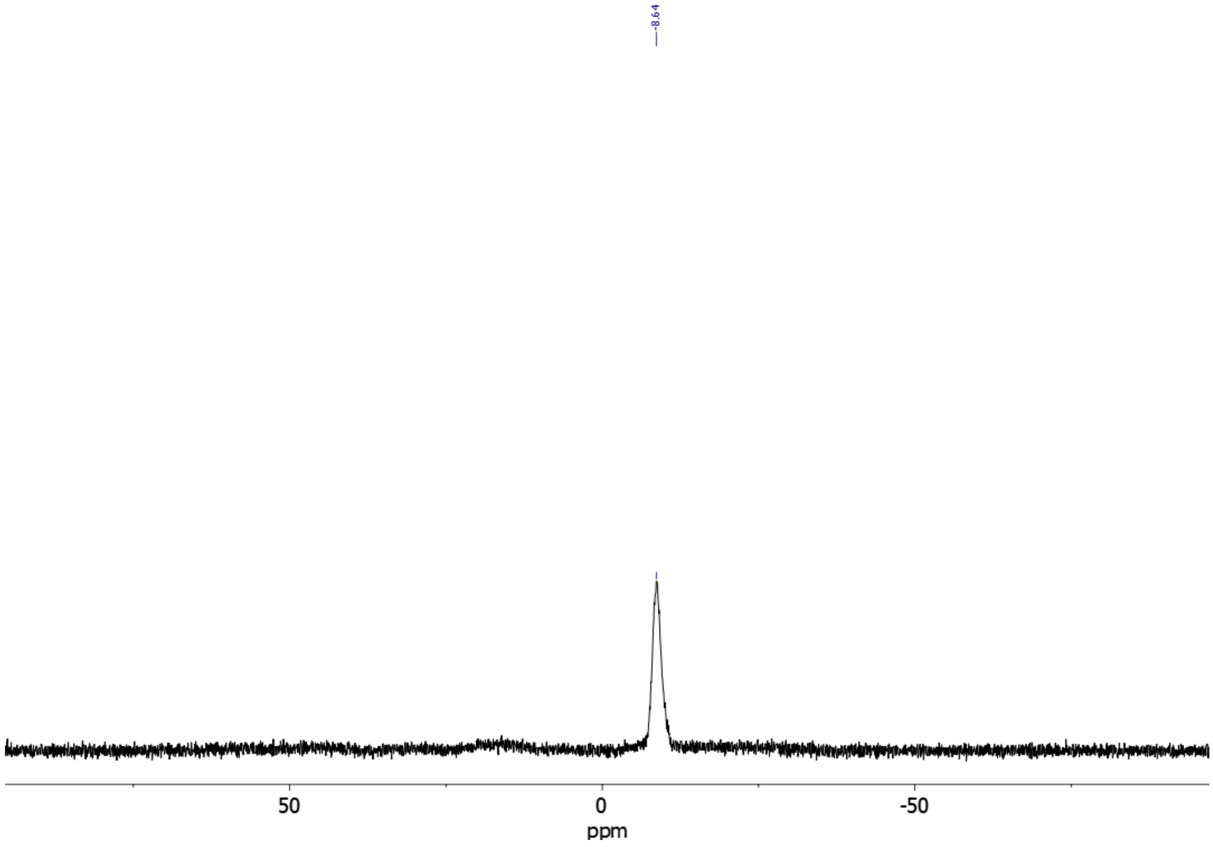

Figure S16. ^{11}B NMR of **[3]OTf** (192 MHz, CD_2Cl_2), Baseline corrected by the subtraction of the borosilicate glass ^{11}B signal.

1.4.5. NMR spectra for [4]OTf

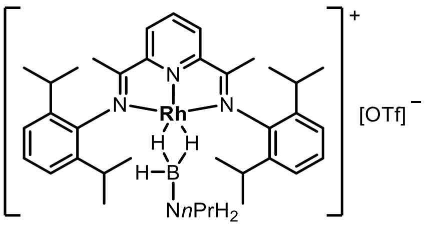

[4]OTf

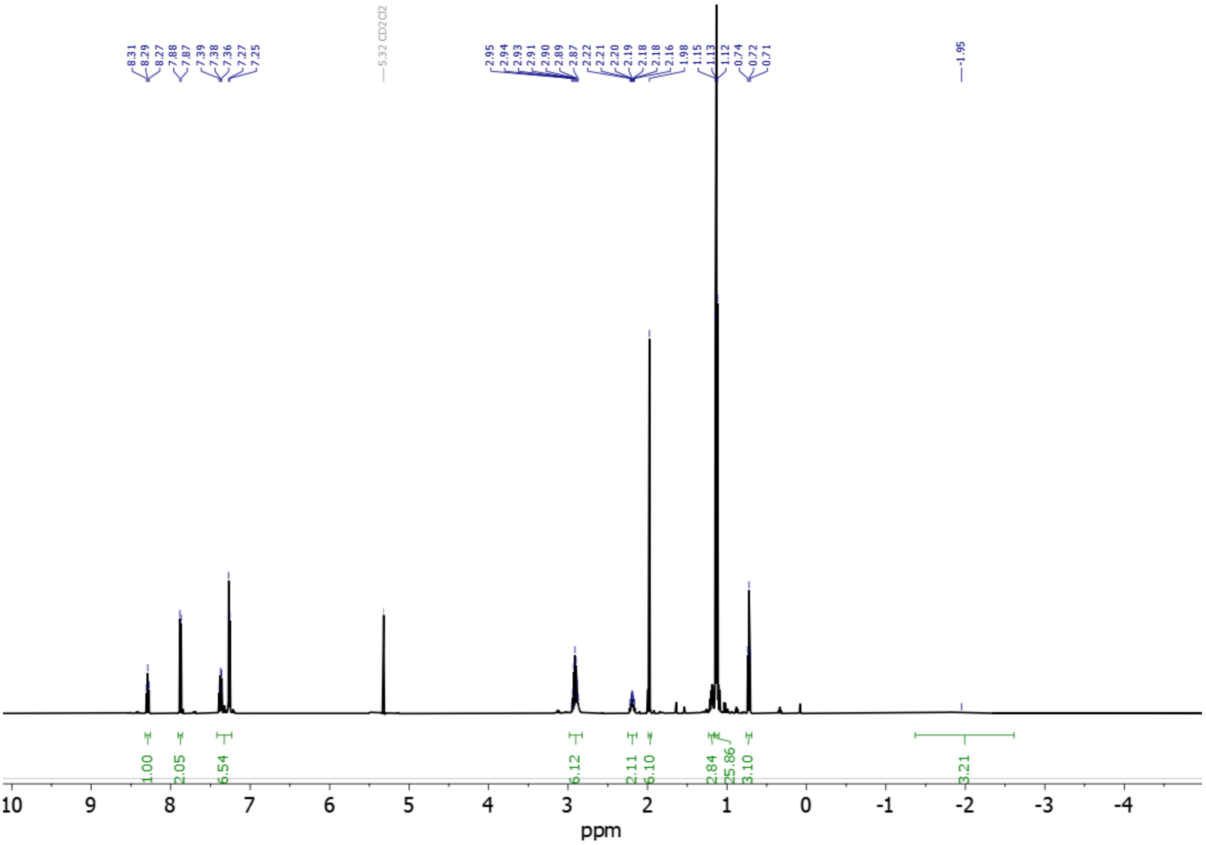

Figure S17. ^{1}H NMR of **[4]OTf** (500 MHz, CD_2Cl_2)

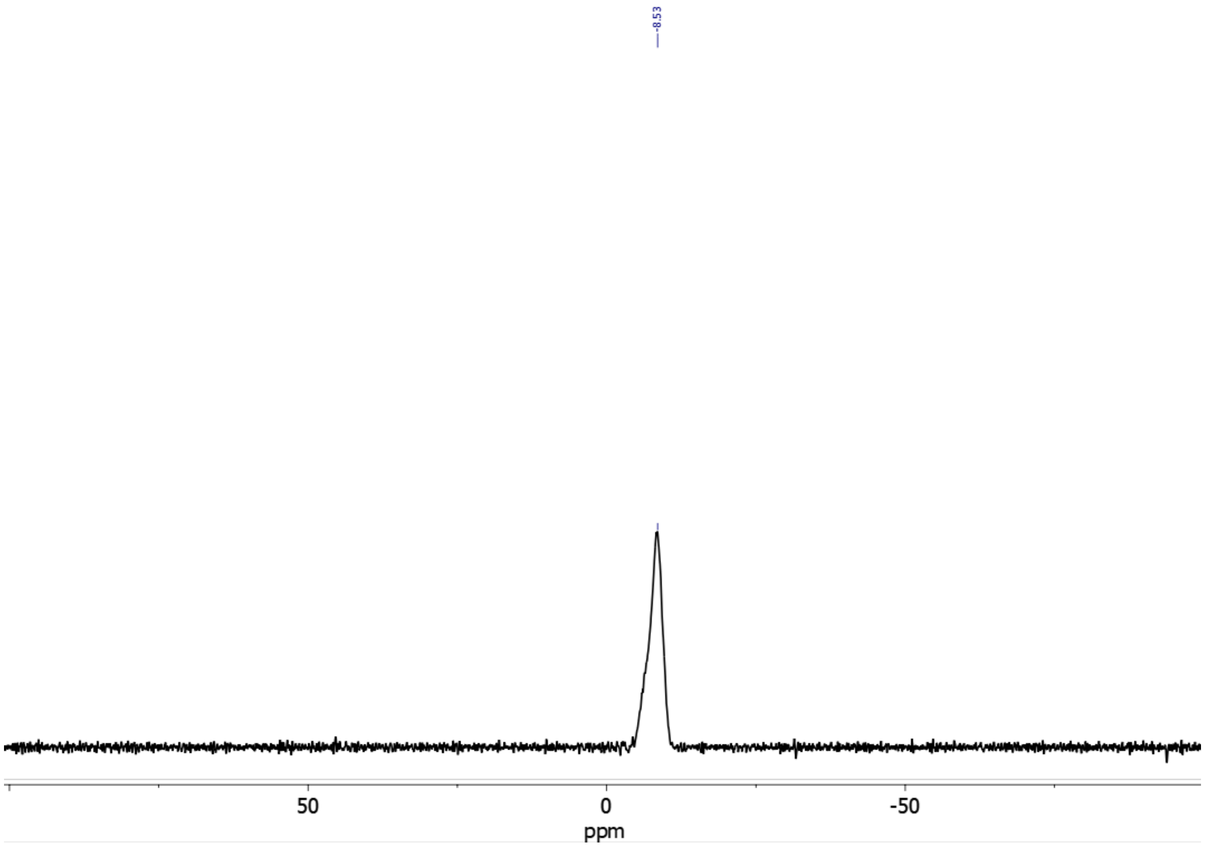

Figure S18. ^{11}B NMR of **[4]OTf** (160 MHz, CD_2Cl_2). Baseline corrected by the subtraction of the borosilicate glass ^{11}B signal.

1.4.6. NMR spectra for 5-OTf

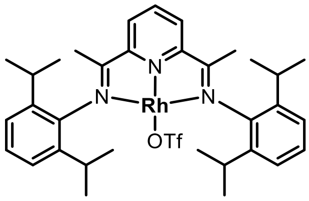

5-OTf

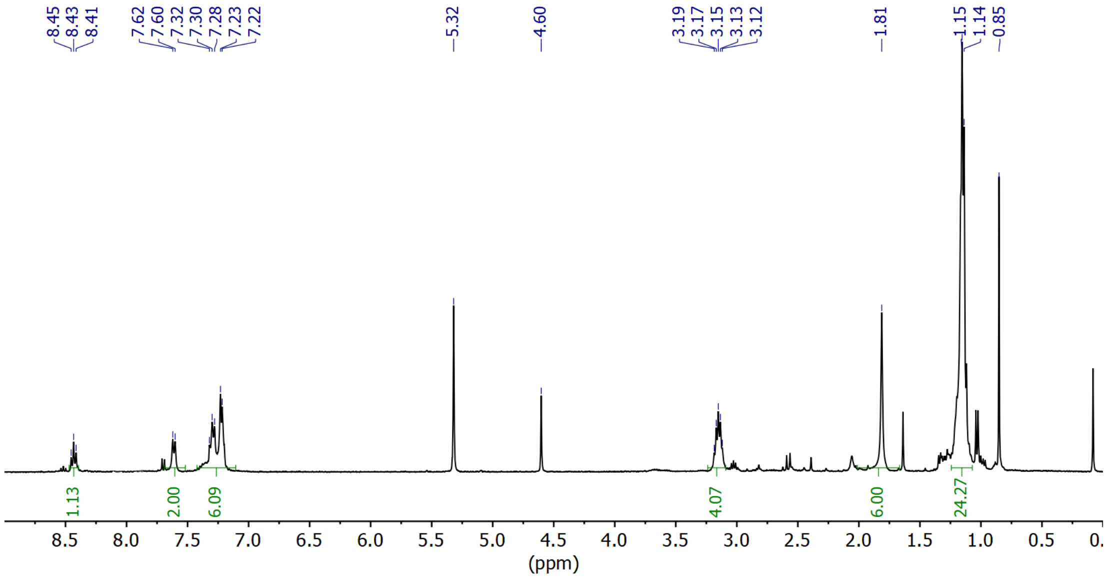

Figure S19. ^{1}H NMR of **5-OTf** (400 MHz, CD_2Cl_2)

1.4.7. NMR spectra for [6]OTf

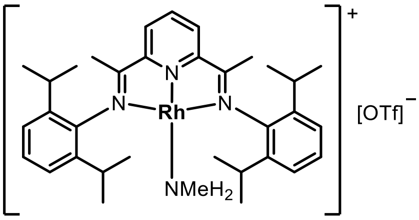

[6]OTf

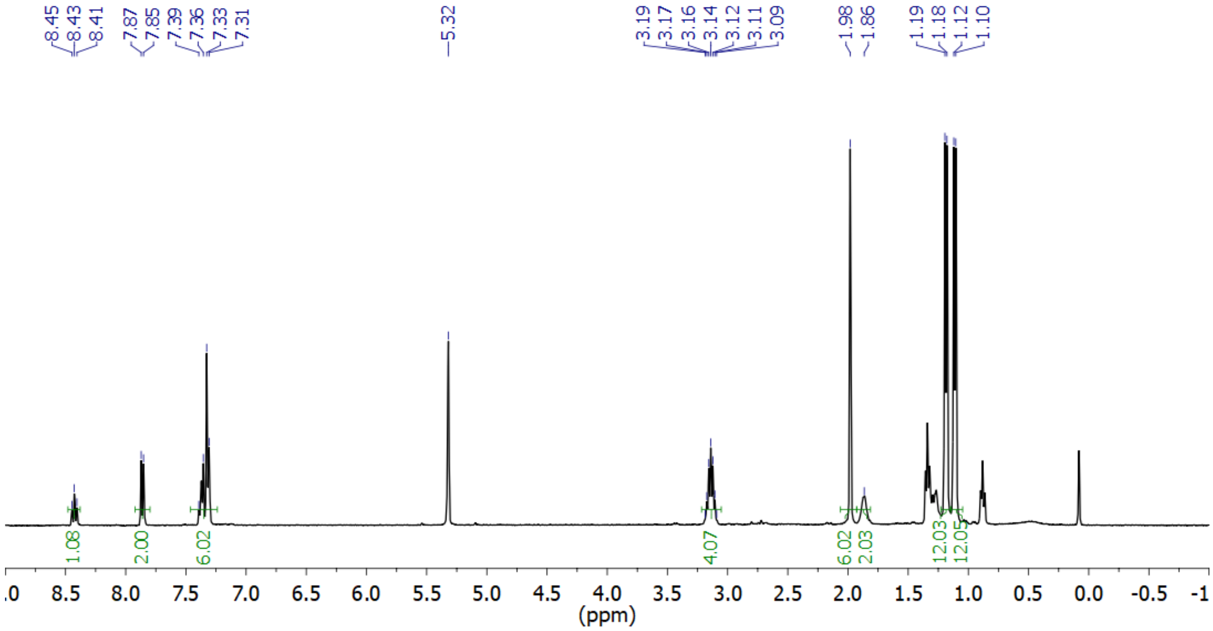

Figure S20. ^{1}H NMR of **[6]OTf** (400 MHz, CD_2Cl_2)

1.4.8. NMR spectra for [6]BAr^F$_4$

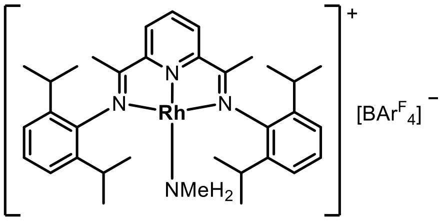

[6]BAr^F$_4$

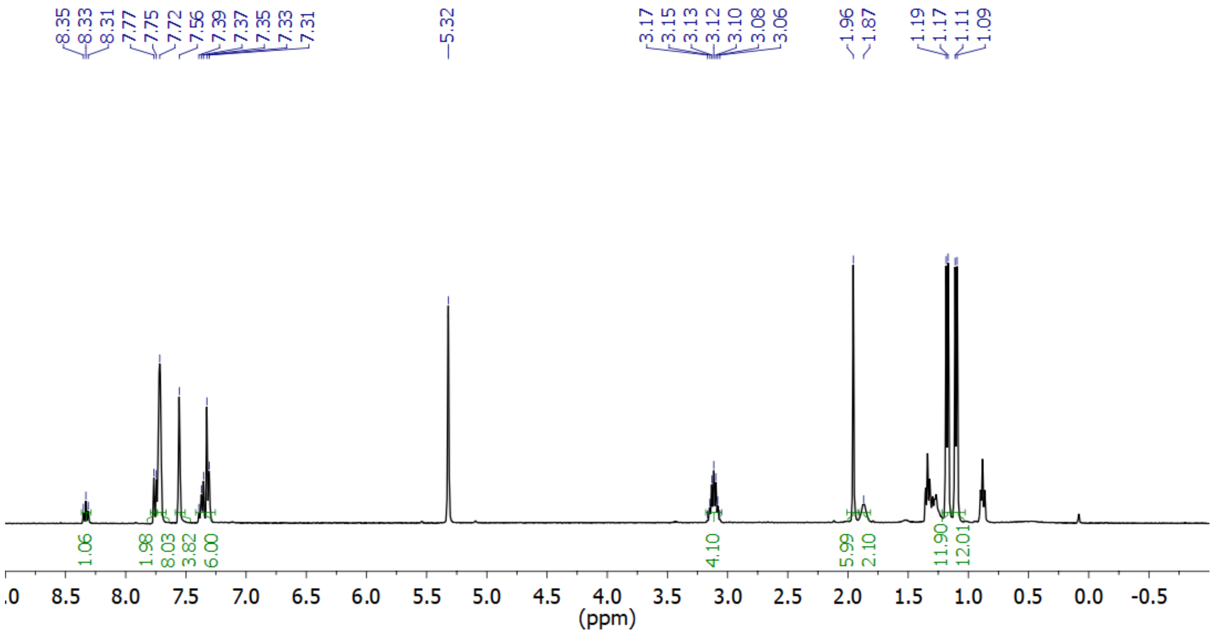

Figure S21. ^{1}H NMR of **[6]BAr^F$_4$** (400 MHz, CD_2Cl_2)

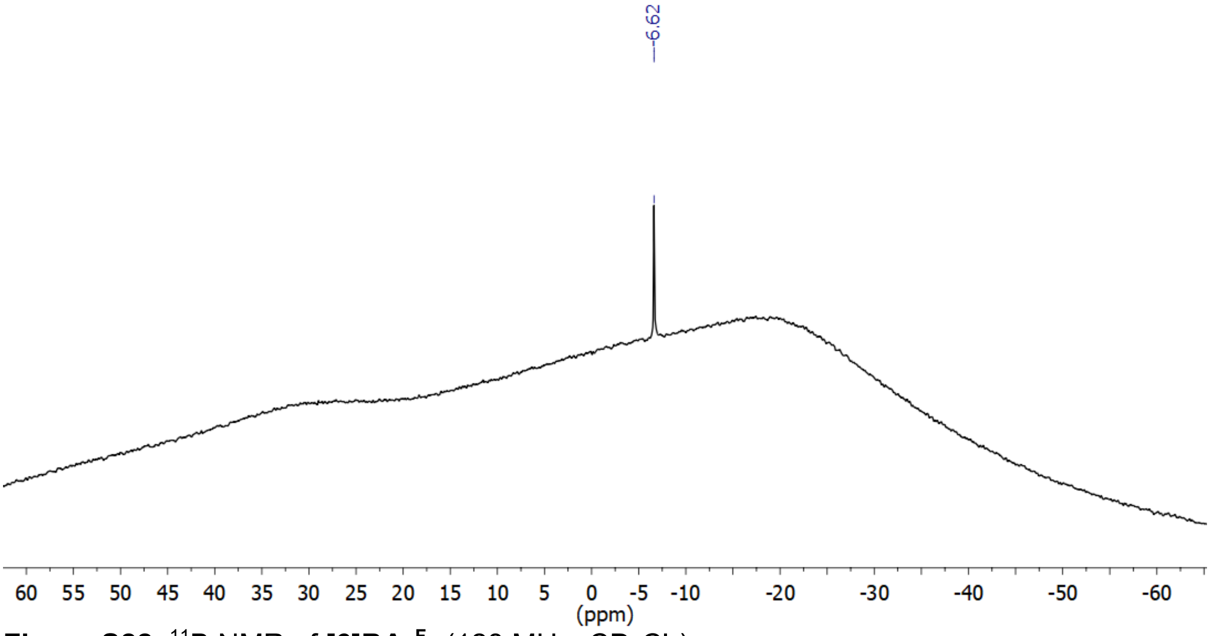

Figure S22. ^{11}B NMR of **[6]BAr^F$_4$** (128 MHz, CD_2Cl_2)

1.5. NMR-scale experiments

1.5.1. NMR investigation into the initial reaction of [6]OTf with Methylamine-Borane

[6]OTf (1.0 mg, 0.0013 mmol) and $H_3B{\cdot}NMeH_2$ (0.6 mg, 0.013 mmol) were added to a Youngs tap NMR tube and CD_2Cl_2 (~0.5 ml) was vacuum transferred in. The solution was then immediately frozen. Upon thawing to 298 K a ^{1}H NMR spectrum as immediately recorded, showing the presence of **[2]OTf** and an unidentified **L1**-containing species.

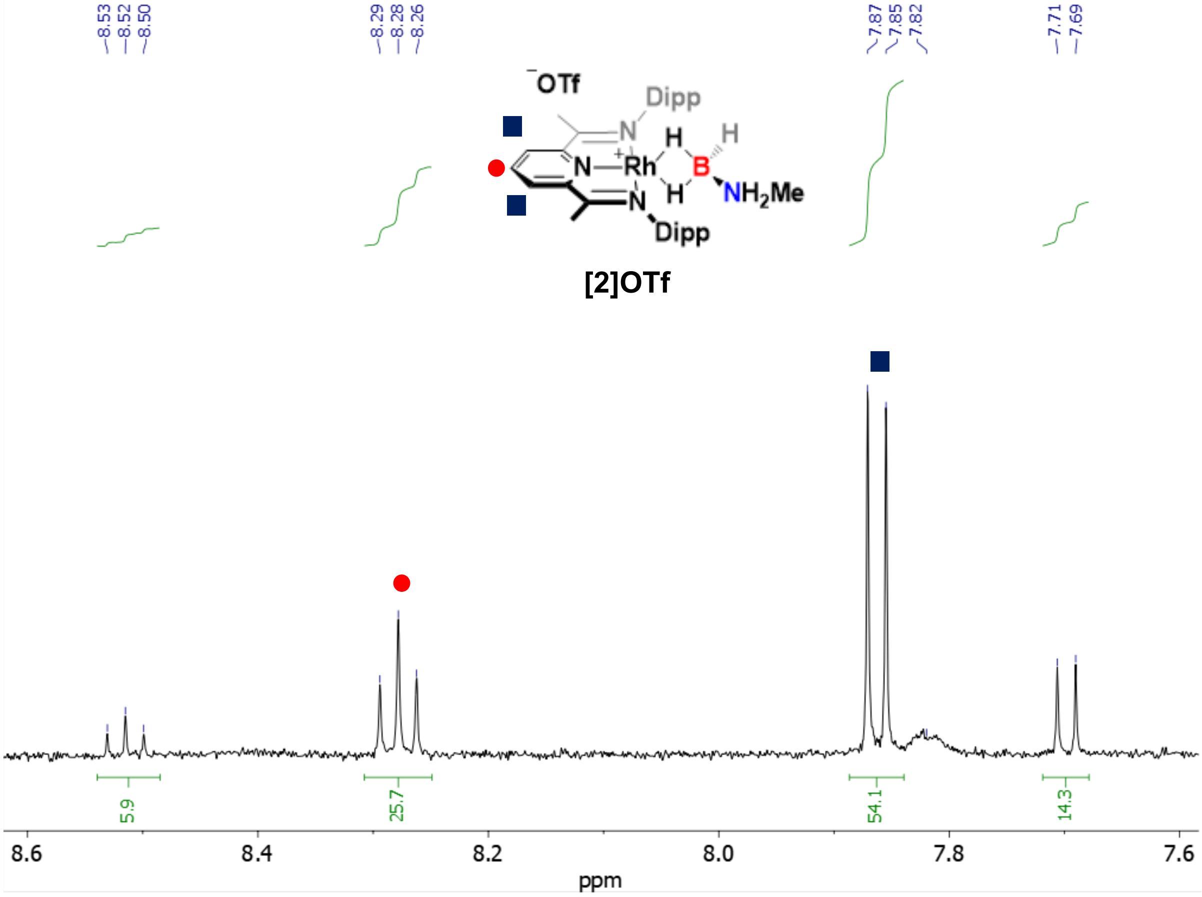

Figure S23. ^{1}H NMR (CD_2Cl_2) spectrum of **[6]OTf** + 10 Eq. of $H_3B{\cdot}NMeH_2$, expanded aromatic region showing pyridyl para- (triplets) and meta- (doublets) protons of **[2]OTf** and another unknown L1-containing species. Protons corresponding to **[6]OTf** are absent.

1.5.2. Solution-stability investigation of [2]OTf in CD_2Cl_2

[2]OTf (~1.0 mg, 0.0013 mmol) was added to a Youngs tap NMR tube and CD_2Cl_2 (~0.5 ml) was vacuum transferred in. A ^{1}H NMR spectrum was recorded after 1 hour, then repeated ^{1}H NMR spectra were recorded upon standing for a further 3 and 11 days.

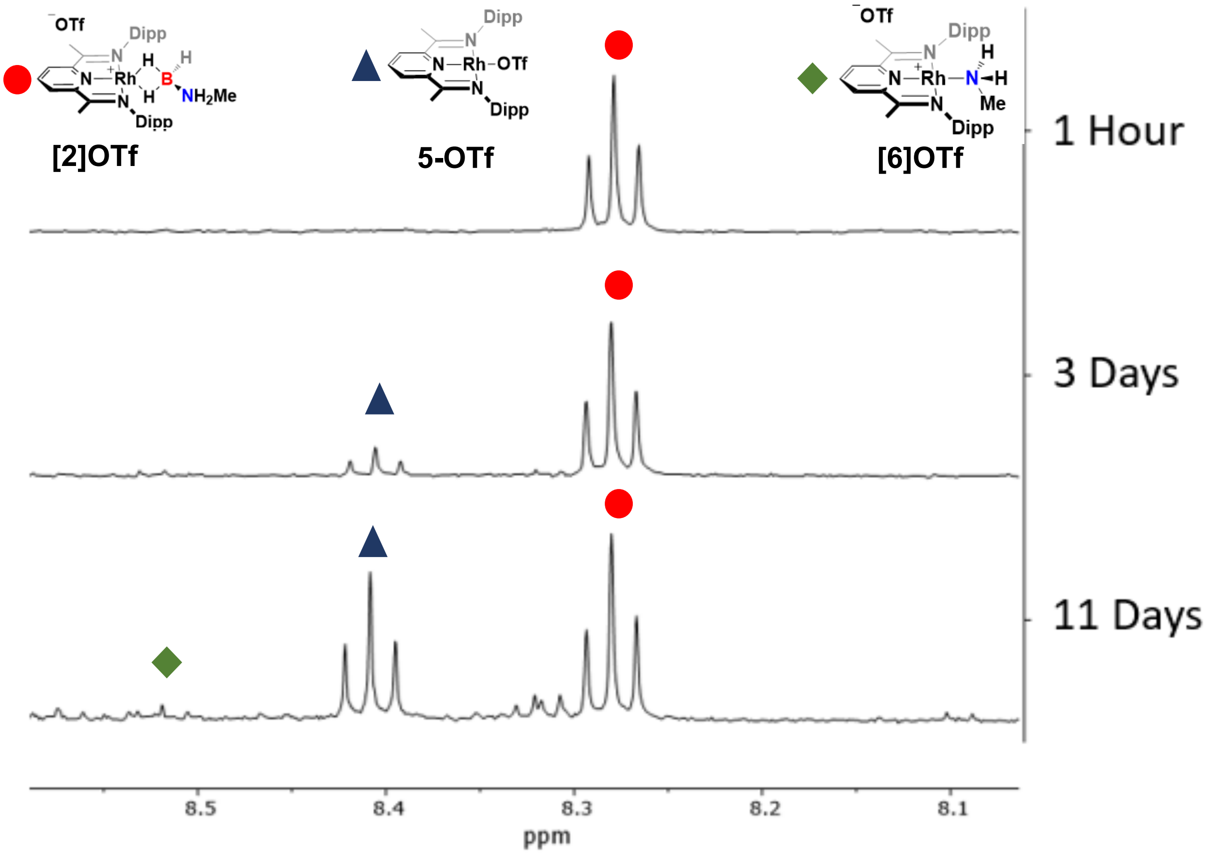

Figure S24. Stacked ^{1}H NMR (CD_2Cl_2) showing the slwo decomposition of **[2]OTf** in solution. Expanded aromatic region showing pyridyl para- protons (triplets) of **[2]OTf**, **5-OTf** and **[6]OTf**. Initially only starting **[2]OTf** is present. After 3 days **[2]OTf** and a small amount of **5-OTf** are present. After 11 days, **[2]OTf**, **5-OTf** and a small amount of **[6]OTf** are present.

1.6. Catalysis

1.6.1. Dehydropolymerisation of Methylamine-borane with [2]OTf

$H_3B{\cdot}NMeH_2$ (50 mg, 1.11 mmol) was suspended in 1,2-$C_6H_4F_2$ (0.5 ml) in a jacketed three-necked Schlenk flask connected to a recirculating cooler and the temperature set to 25 °C. The jacketed Schlenk was then sealed off from the Ar supply and connected to a water-filled gas burette. Separately, **[2]OTf** (8.7 mg, 0.011 mmol) was dissolved in 1,2-$C_6H_4F_2$ (2 ml) to give a dark-green solution. The **[2]OTf** precatalyst solution (1 mol% relative to $H_3B{\cdot}NMeH_2$) was then transferred into the jacketed Schlenk and the resulting dark blue-green suspension was stirred at 400 rpm. The total volume of 1,2-$C_6H_4F_2$ was 2.5 ml, giving a nominal $H_3B{\cdot}NMeH_2$ concentration of 0.446 M. The time taken for H_2 gas to be evolved was recorded. After 45 minutes of stirring, no gas had been evolved and so $MeNH_2$ (25 µl, 2 mol dm^{-3} in THF, 5 Eq. to precatalyst) was added, resulting in immediate H_2 evolution and dark-red colouration of the reaction mixture, which changed to brown and finally grey over the course of H_2 evolution. Upon completion of gas evolution, the produced poly(N-methylaminoborane) was precipitated by the addition of pentane (~50 ml), which was filtered and dried under vacuum overnight yielding a grey solid (30 mg, 68 %). The molecular weight of the polymer produced was investigated by GPC: M_n: 29,700 (Da), M_w: 41,900 (Da), PDI: 1.4.

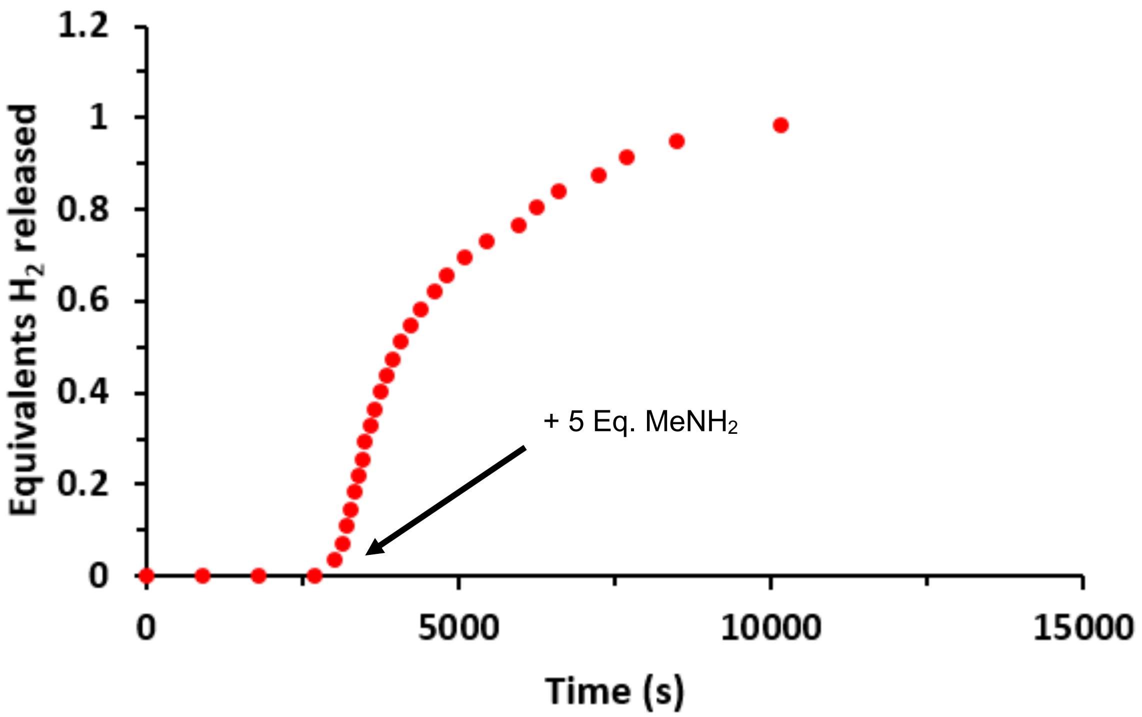

Figure S25. Equivalents of H_2 released over time for dehydropolymerisation of $H_3B{\cdot}NMeH_2$ (0.446 M) in 1,2-difluorobenzene, using **[2]OTf** (1 mol% cat), with $MeNH_2$ (25 µl, 2 mol dm^{-3}, 5 Eq. to precatalyst) added after 45 minutes.

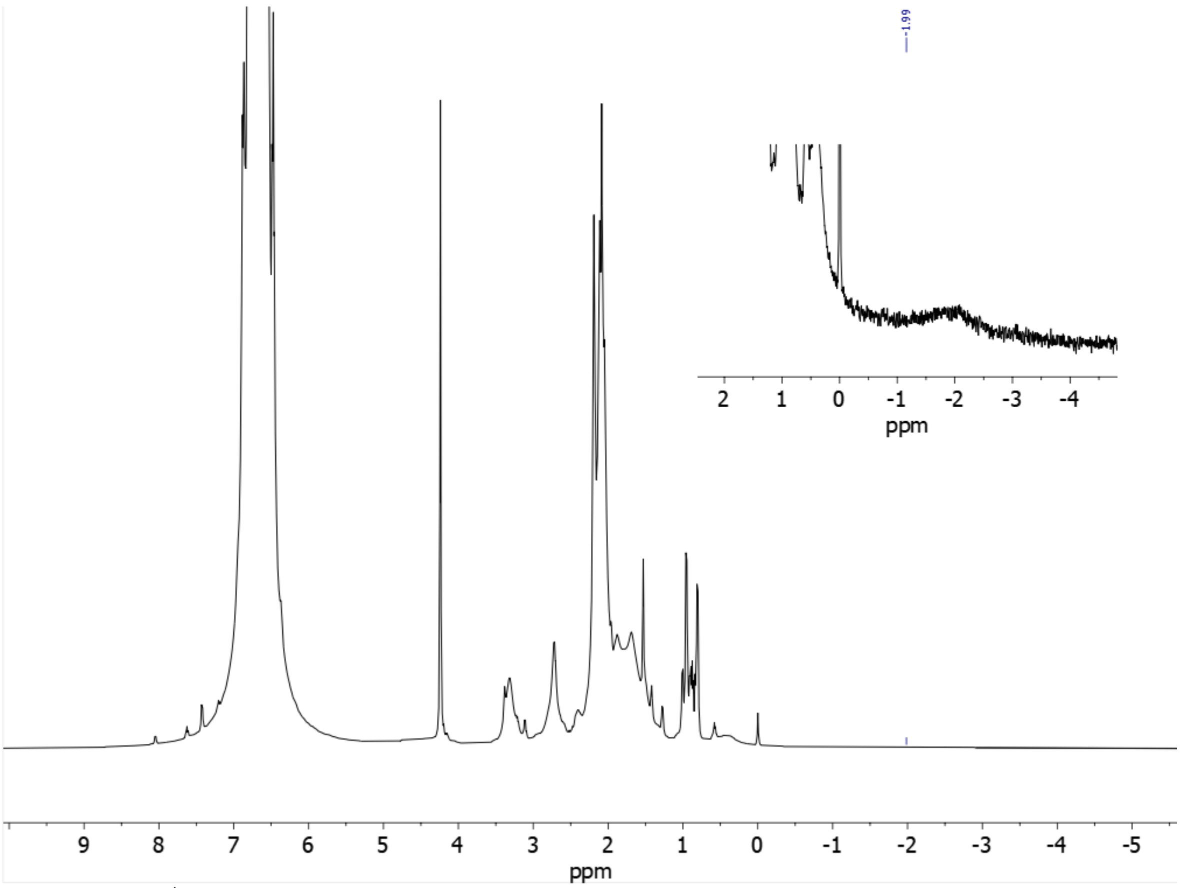

Figure S26. ^{1}H NMR (600 MHz, 1,2-$C_6H_4F_2$) *in-situ* of catalysis reaction mixture before the addition of amine (at 45 minutes, 2000 seconds) showing obscured aliphatic and aromatic regions by $H_3B{\cdot}NMeH_2$ and 1,2-$C_6H_4F_2$ respectively. Expansion showing the presence of a broad, low-intensity signal at ~ –2 ppm, likely corresponding to unreacted **[2]OTf**.

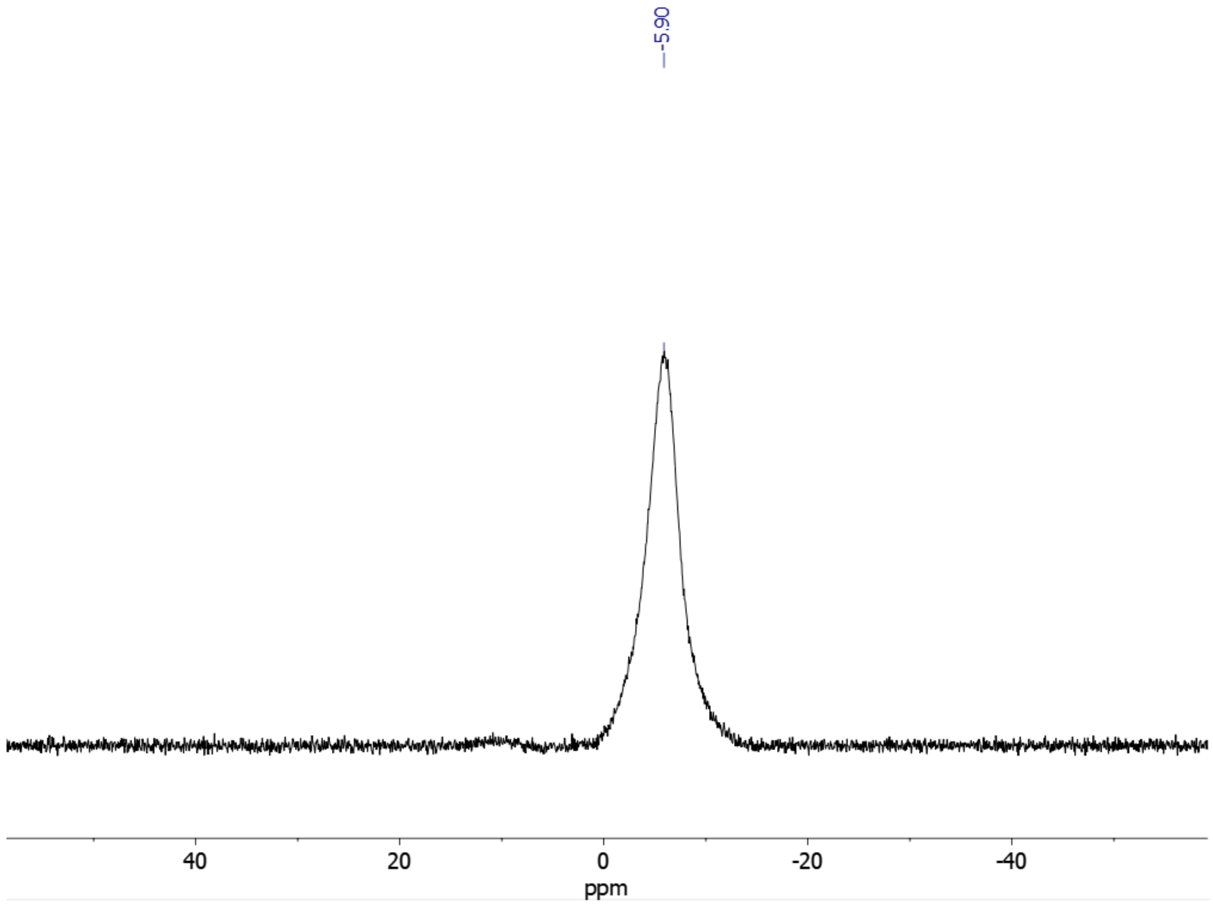

Figure S27. ^{11}B NMR (192 MHz, $CDCl_3$) spectrum of the polymer precipitated by the addition of pentane to the catalysis mixture, $(H_2B{\cdot}NMeH)_n$. Baseline corrected by the subtraction of the borosilicate glass [11]B signal.

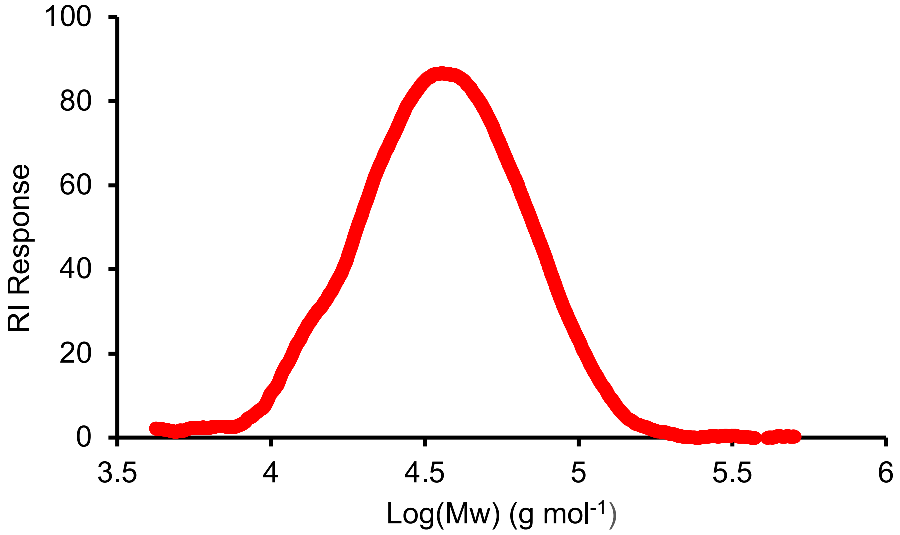

Figure S28. GPC trace of polymer, $(H_2B{\cdot}NMeH)_n$, from the dehydropolymerisation of $H_3B{\cdot}NMeH_2$ with **[2]OTf** in the presence of excess amine (5 Eq. to precatalyst).

1.6.2. Dehydropolymerisation of methylamine-borane with [6]OTf

$H_3B{\cdot}NMeH_2$ (50 mg, 1.11 mmol) was suspended in 1,2-$C_6H_4F_2$ (2 ml) in a jacketed three-necked Schlenk flask connected to a recirculating cooler and the temperature set to 25 °C. The jacketed Schlenk was then sealed off from the Ar supply and connected to a water-filled gas burette. Separately, **[6]OTf** (8.5 mg, 0.011 mmol) was dissolved in 1,2-$C_6H_4F_2$ (0.5 ml). The **[6]OTf** precatalyst solution (1 mol% relative to $H_3B{\cdot}NMeH_2$) was then transferred into the jacketed Schlenk and the resulting dark blue-green suspension was stirred at 400 rpm. The total volume of 1,2-$C_6H_4F_2$ was 2.5 ml, giving a nominal $H_3B{\cdot}NMeH_2$ concentration of 0.446 M. The time taken for H_2 gas to be evolved was recorded. Immediate H_2 evolution was observed and over 15 minutes the reaction mixture turned dark-red, which changed to brown and finally grey over the course of H_2 evolution. Upon completion of gas evolution, the produced poly(N-methylaminoborane) was precipitated by the addition of pentane (~50 ml), which was filtered and dried under vacuum overnight yielding a grey solid (37 mg, 77 %). The molecular weight of the polymer produced was investigated by GPC: M_n: 20,500 (Da), M_w: 28,300 (Da), PDI: 1.4.

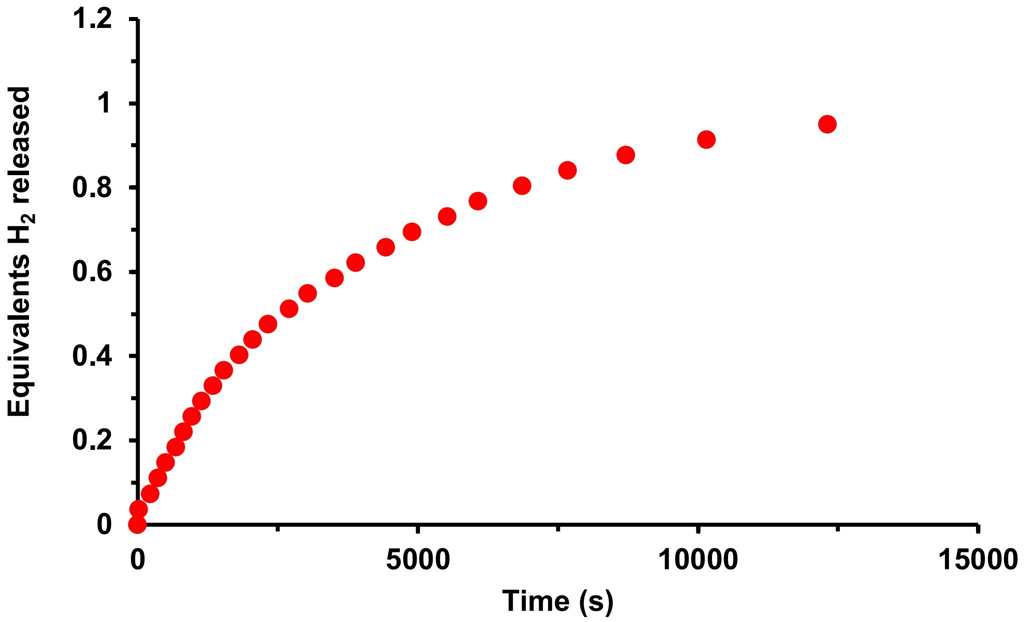

Figure S29. Equivalents of H_2 released over time for dehydropolymerisation of $H_3B{\cdot}NMeH_2$ (0.446 M) in 1,2-difluorobenzene, using **[6]OTf** (1 mol% cat).

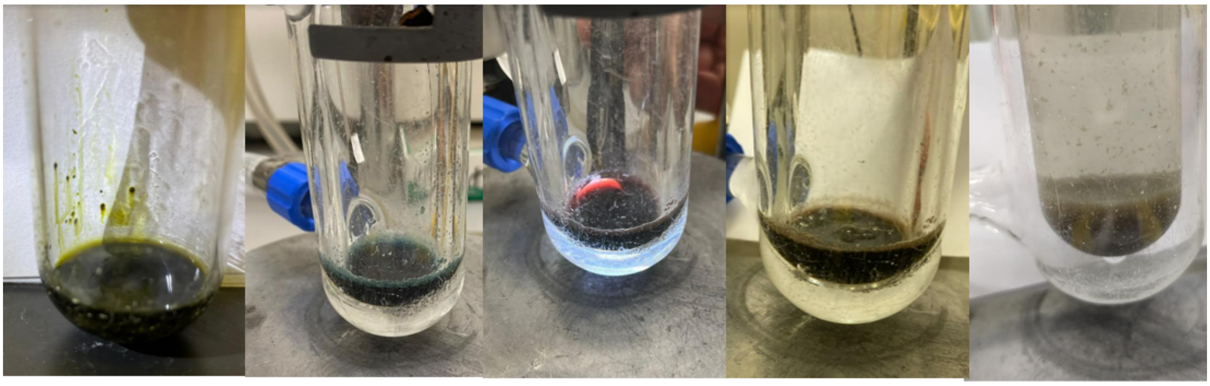

Figure S30. Images of the catalysis mixture at varying timepoints of the dehydropolymerisation of $H_3B·NMeH_2$ with **[6]OTf**. (Left to right) 1: **[6]OTf** precatalyst solution. 2: Blue-green colouration upon addition of the **[6]OTf** precatlyst solution to methylamine-borane. 3: Dark-red colouration at 15 minutes post precatalyst addition. 4: Brown colouration one-hour post precatalyst addition. 5: Grey precipitate $(H_2B·NMeH)_n$ and yellow supernatant after addition of excess pentane to the reaction mixture.

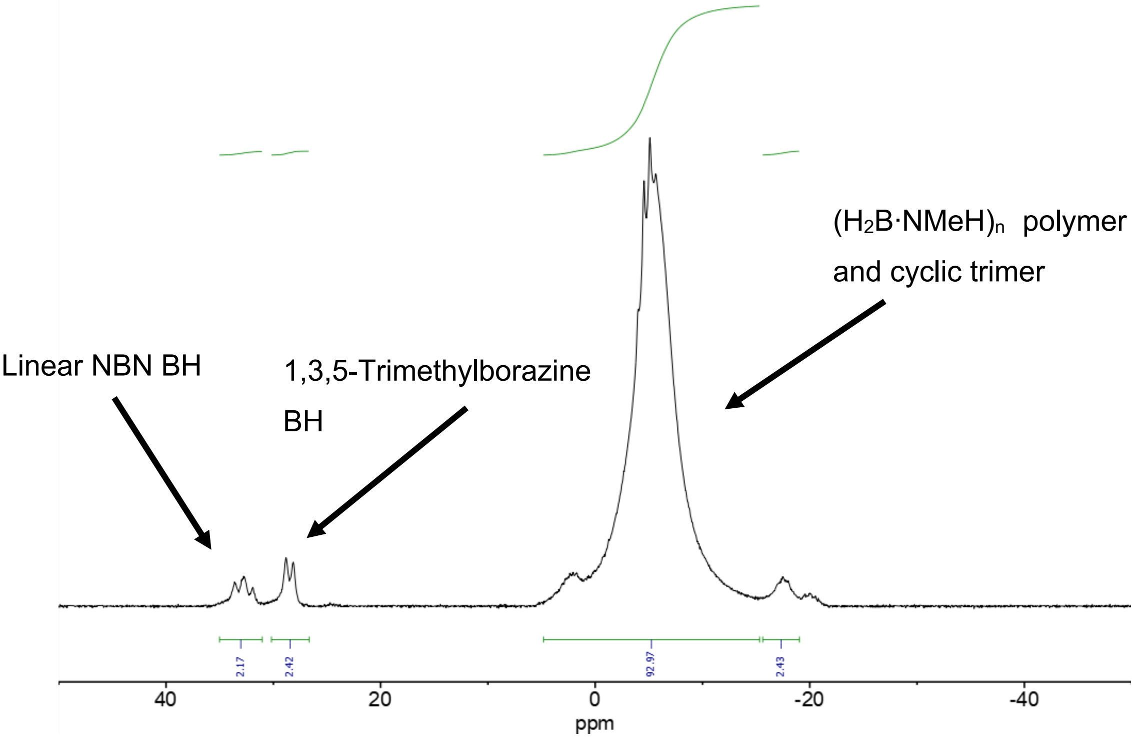

Figure S31. ^{11}B NMR (192 MHz, 1,2-$C_6H_4F_2$) *in-situ* of catalysis reaction mixture after 15,000 seconds showing polymer $(H_2B·NMeH)_n$ and other BN side products. Baseline corrected by the subtraction of the borosilicate glass ^{11}B signal.

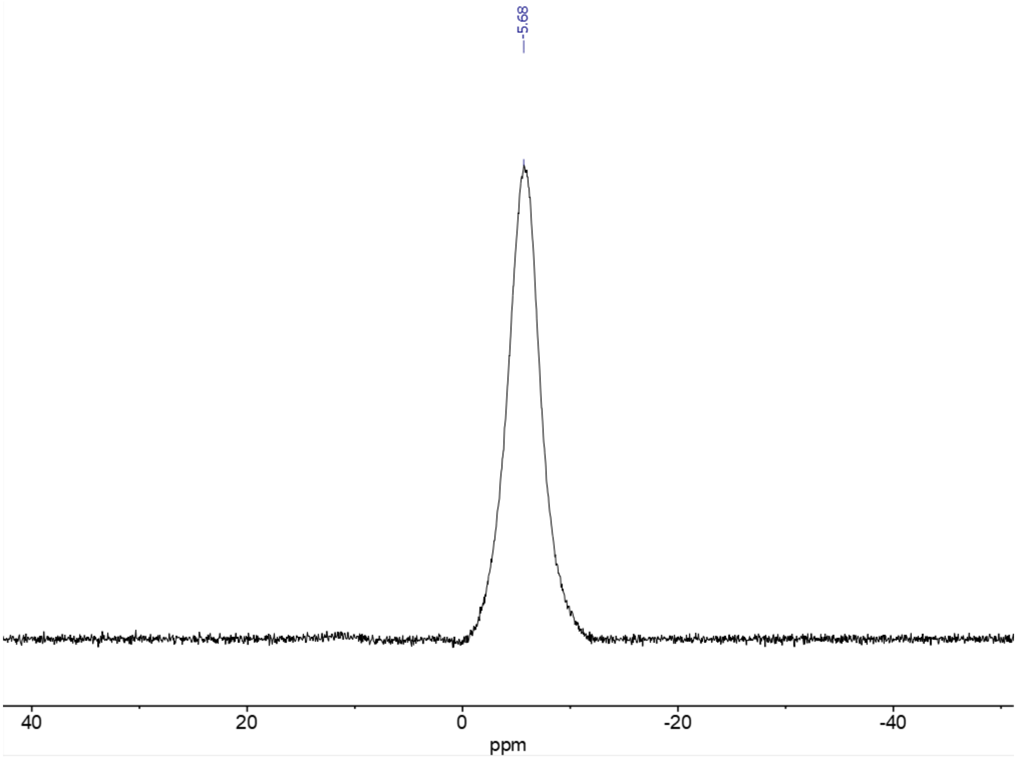

Figure S32. ^{11}B NMR (192 MHz, $CDCl_3$) spectrum of the polymer precipitated by the addition of pentane to the catalysis mixture, $(H_2B{\cdot}NMeH)_n$. Baseline corrected by the subtraction of the borosilicate glass ^{11}B signal.

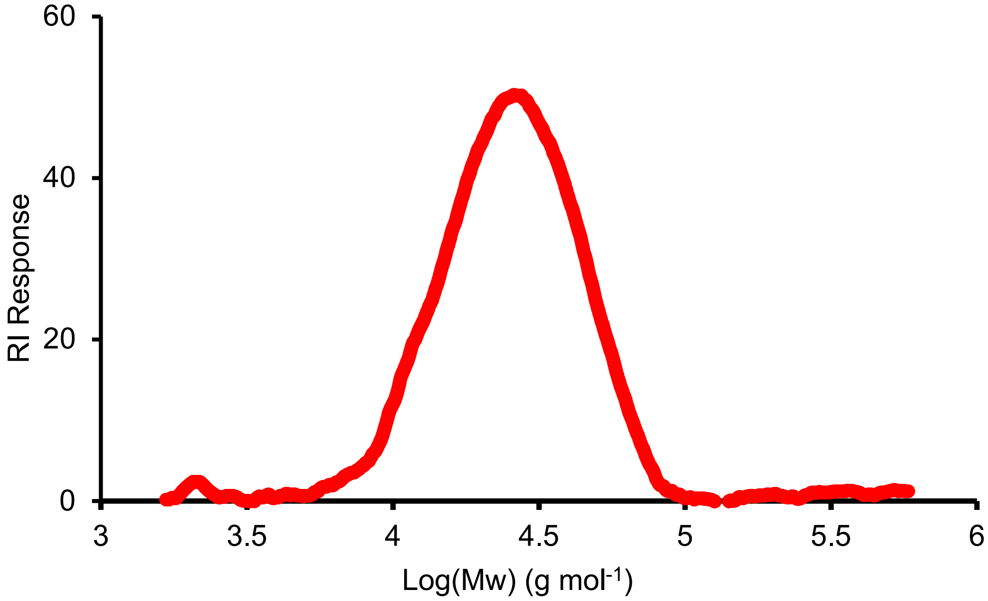

Figure S33. GPC trace of polymer, $(H_2B{\cdot}NMeH)_n$, from the dehydropolymerisation of $H_3B{\cdot}NMeH_2$ with **[6]OTf**.

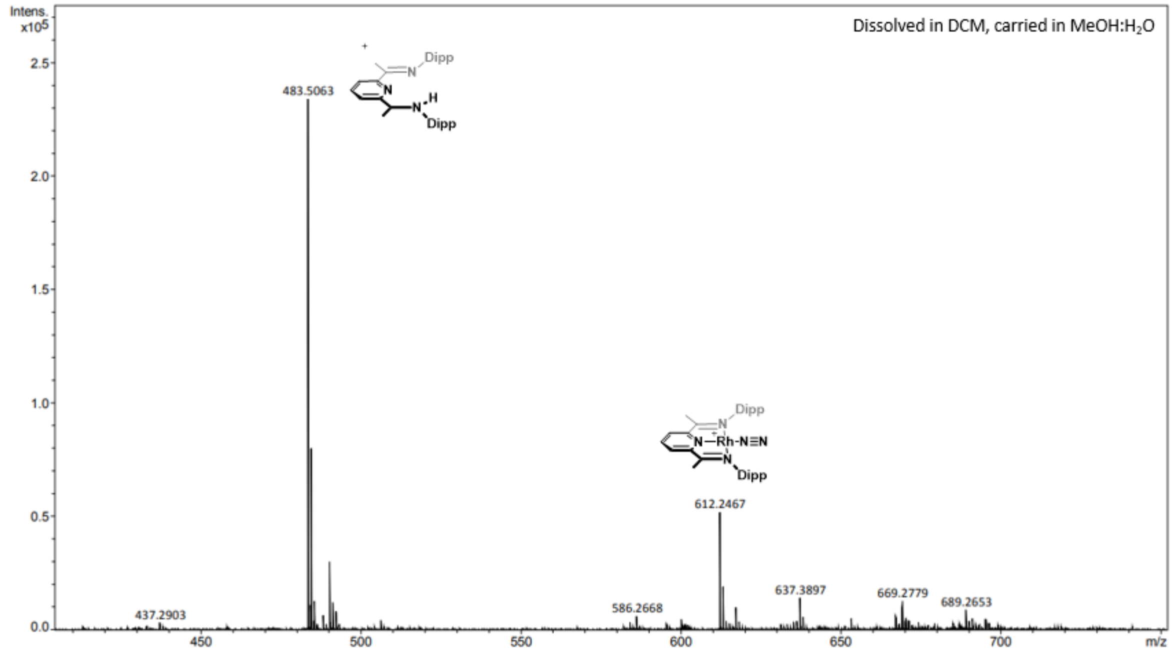

Figure S34. *In-situ* ESI+ mass spectrum recorded upon the completion of hydrogen evolution from the reaction mixture. Dissolved in CH_2Cl_2 and carried in 1:1 MeOH:H_2O. Partially hydrogenated ligand (**L1**H_2) and an adduct of [Rh(**L1**)]$^+$ and the N_2 carrier gas.

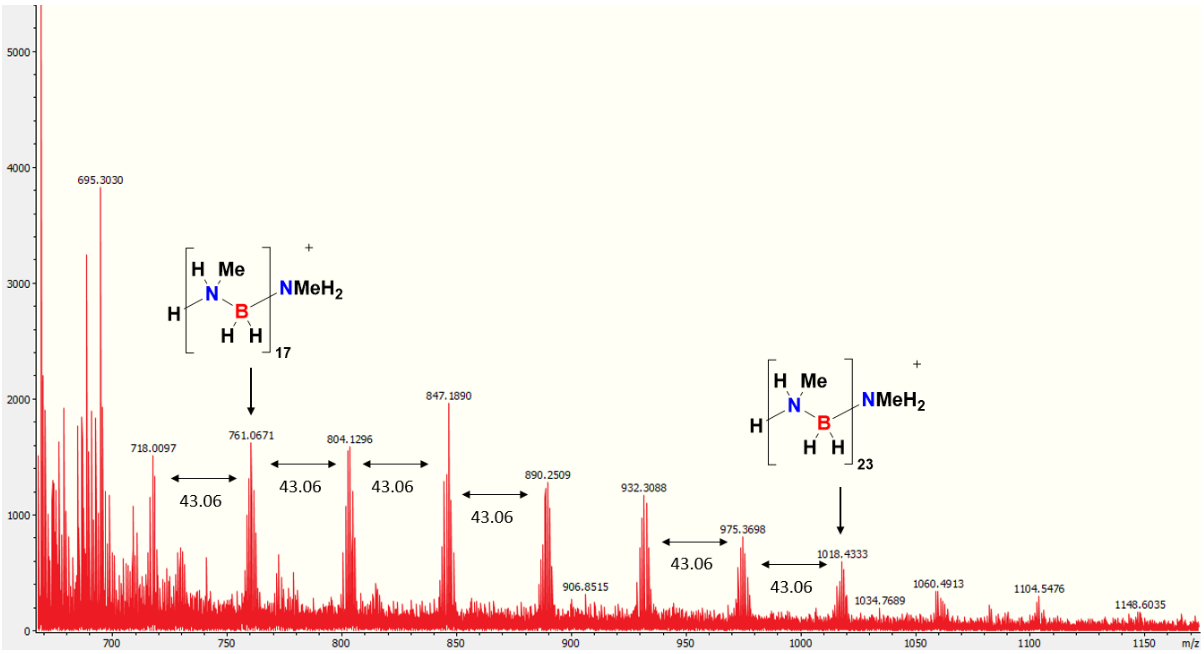

Figure 35. *In-situ* ESI+ mass spectrum recorded upon the completion of hydrogen evolution from the reaction mixture. Dissolved in CH_2Cl_2 and carried in 1:1 MeOH:H_2O. Expanded to show the presence of poly(N-methylaminoborane) capped with $MeNH_2$ end-groups.

1.6.3. Dehydropolymerisation of Methylamine-Borane with [6]OTf – Pre-activated catalyst with NMR investigation

[6]OTf (4.1 mg, 0.0055 mmol), DABCO (1.2 mg, 0.011 mmol) and $H_3B{\cdot}NMeH_2$ (0.5 mg, 0.011 mmol) were dissolved in 1,2-$C_6H_4F_2$ (0.5 ml) in a Youngs tap NMR tube. ^1H NMR spectra were then recorded every minute for 5 minutes with 30 seconds of shaking to mix between spectra, during which the dark-green solution became dark-red and **[2]OTf** is observed and then decays. After 5 minutes, a 30-minute-long ^1H NMR spectrum as recorded where a low concentration of **7** is observed. $H_3B{\cdot}NMeH_2$ (50 mg, 1.11 mmol) was dissolved in 1,2-$C_6H_4F_2$ (2 ml) in a jacketed three-neck Schlenk flask connected to a recirculating cooler and the temperature set at 25 °C. The jacketed Schlenk was then sealed off from the Ar supply and connected to a water-filled gas burette. The pre-activated catalyst (35 minutes after initial mixing) solution was then transferred from the NMR tube into the jacketed Schlenk (effective 0.5 mol% catalyst) and the resulting solution was stirred at 400 rpm (0.5 mol% due to solubility limits of **[6]OTf** in 0.5 cm^3 of 1,2-$C_6H_4F_2$). The total volume of 1,2-$C_6H_4F_2$ was 2.5 ml, giving a $H_3B{\cdot}NMeH_2$ concentration of 0.446 M. The time taken for H_2 gas to be evolved was recorded. Upon completion of gas evolution, the solution was decanted into 50 ml of rapidly stirring pentane to give an off-white suspension which was stirred for 5 minutes to allow polymer precipitation, then isolated by filtration. The grey solid $(H_2B{\cdot}NMeH)_n$ was dried under vacuum overnight. The molecular weight of the polymer produced was investigated by GPC: M_n: 24,500 (Da), M_w: 36,100 (Da), PDI: 1.47.

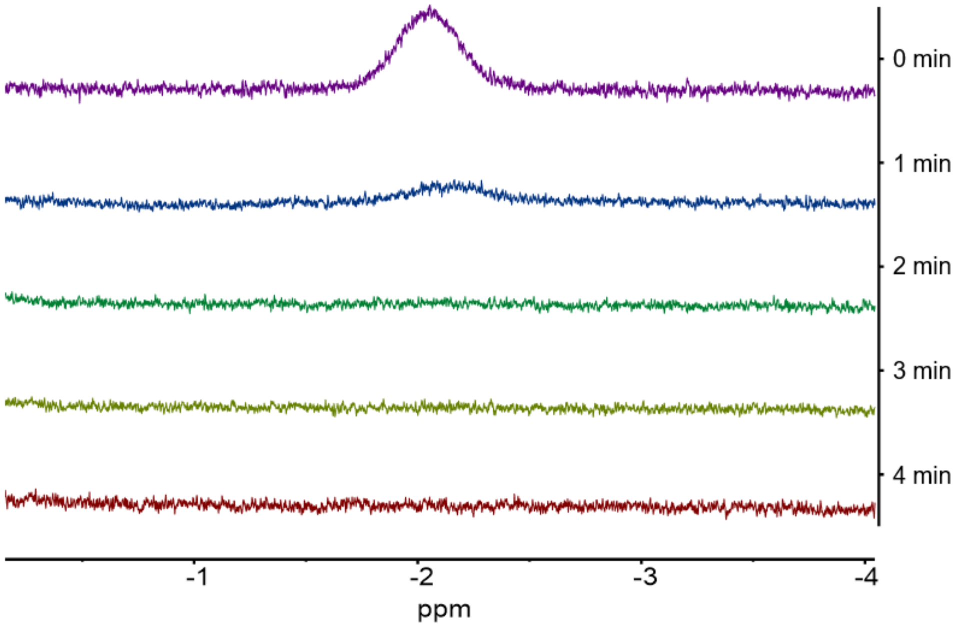

Figure S36. Stacked ^1H NMR (1,2-$C_6H_4F_2$) plots of the precatalyst solution **[6]OTf**/DABCO activation showing initially the presence of σ-amine-borane complex **[2]OTf** (broad singlet at -2 ppm, BH_3) formed by the displacement of $NMeH_2$ from **[6]OTf** by $H_3B{\cdot}NMeH_2$. The subsequent decay over time results from the σ-amine-borane complex intermediate (**[2]OTf**) being converted into the catalytically-active species.

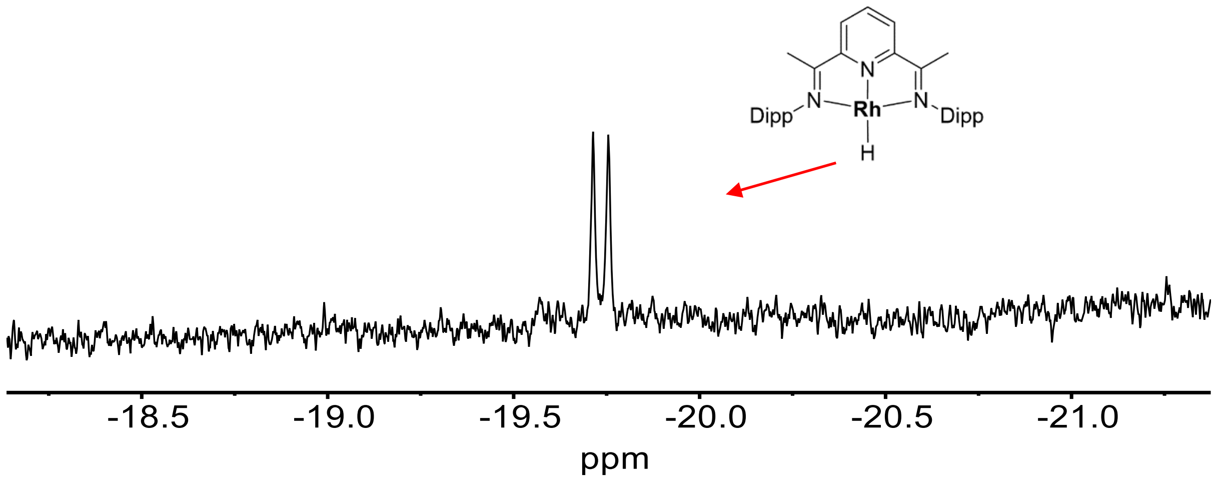

Figure S37. ^{1}H NMR (1,2-$C_6H_4F_2$) excerpt of the precatalyst solution showing a doublet at -19.7 ppm with J = 24.7 Hz. Likely corresponding to the transient intermediate [Rh(L1)H] (**7**). Spectrum recorded over 30 minutes due to low concentration of the intermediate. Rest of the spectrum omitted for clarity due to the relative low concentration of **7**.

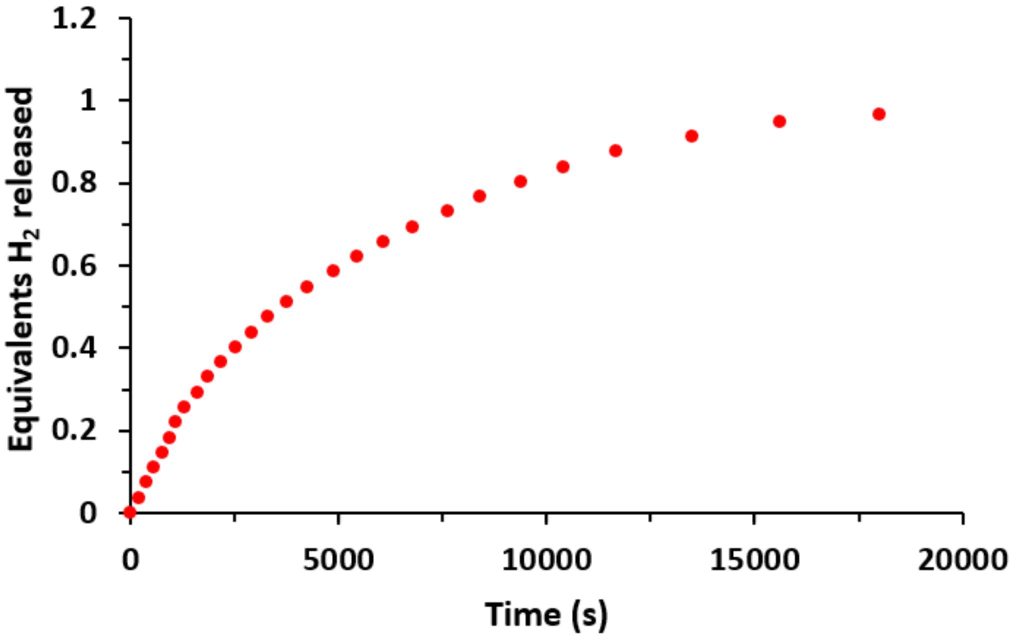

Figure S38. Equivalents of H_2 released over time for dehydropolymerisation of $H_3B{\cdot}NMeH_2$ (0.446 M) in 1,2-difluorobenzene, by **[6]OTf**/DABCO (0.5 mol% cat).

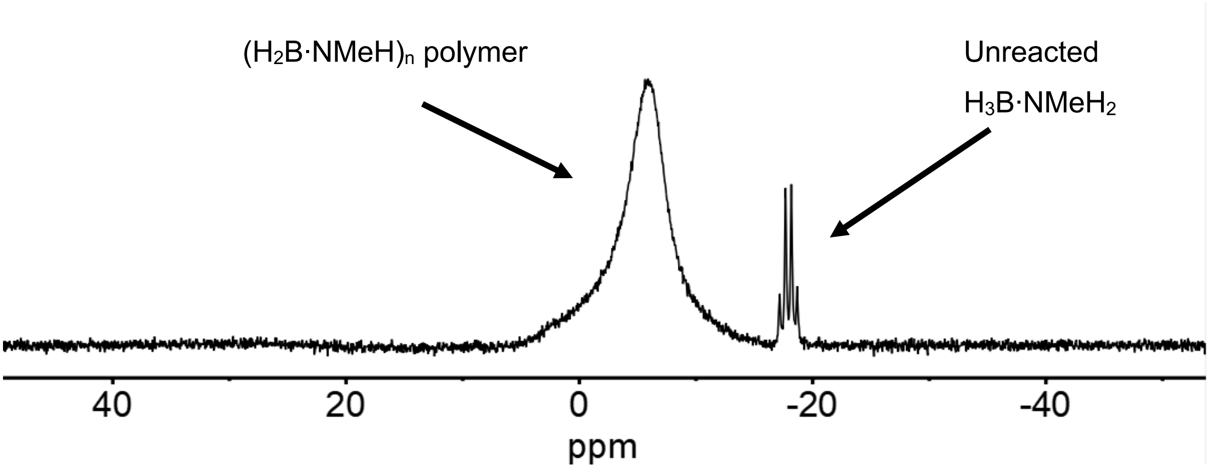

Figure S39. ^{11}B NMR (192 MHz, 1,2-$C_6H_4F_2$) *in-situ* of the catalysis reaction using **[6]OTf**/DABCO (0.5 mol% cat) after 20,000 seconds showing polymer ($H_2B{\cdot}NMeH)_n$ and unreacted $H_3B{\cdot}NMeH_2$. Baseline corrected by the subtraction of the borosilicate glass [11]B signal.

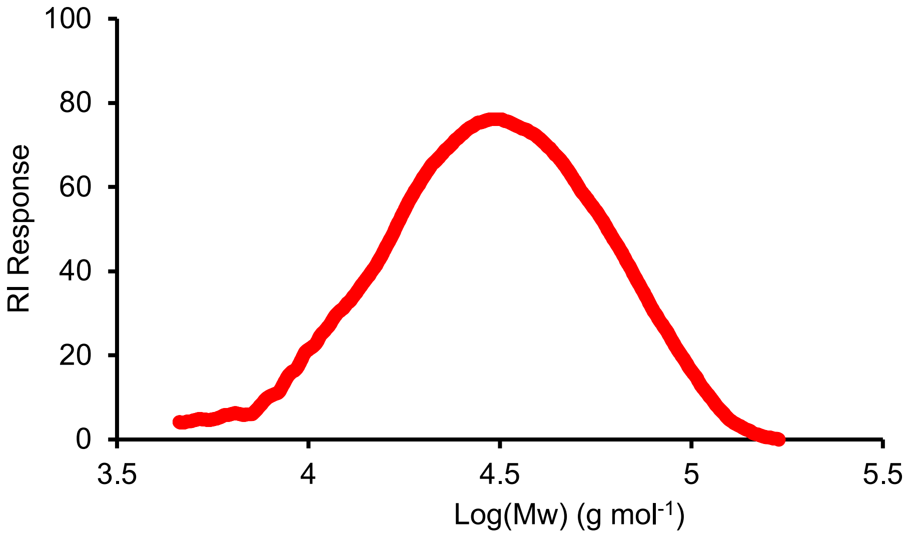

Figure S40. GPC trace of polymer, $(H_2B{\cdot}NMeH)_n$, from the dehydropolymerisation of $H_3B{\cdot}NMeH_2$ with DABCO / **[6]OTf**.

1.6.4. Dehydropolymerisation of Methylamine-Borane with [6]OTf – Recharge experiment

$H_3B{\cdot}NMeH_2$ (50 mg, 1.11 mmol) was suspended in 1,2-$C_6H_4F_2$ (2 ml) in a jacketed three-necked Schlenk flask connected to a recirculating cooler and the temperature set to 25 °C. The jacketed Schlenk was then sealed off from the Ar supply and connected to a water-filled gas burette. Separately, **[6]OTf** (8.5 mg, 0.011 mmol) was dissolved in 1,2-$C_6H_4F_2$ (0.5 ml). The **[6]OTf** precatalyst solution (1 mol% relative to $H_3B{\cdot}NMeH_2$) was then transferred into the jacketed Schlenk and the resulting dark blue-green suspension was stirred at 400 rpm. The time taken for H_2 gas to be evolved was recorded. The total volume of 1,2-$C_6H_4F_2$ was 2.5

ml, giving a nominal $H_3B{\cdot}NMeH_2$ concentration of 0.446 M. Upon completion of this first evolution, the reaction mixture was transferred into a second jacketed three-necked Schlenk flask connected to a recirculating cooler with the temperature set to 25 °C and containing $H_3B{\cdot}NMeH_2$ (50 mg, 1.11 mmol). This Schlenk flask was also connected to a water-filled gas burette and so a second evolution of hydrogen was measured. The second evolution of hydrogen slowed considerably toward the end of catalysis and the reaction mixture was left overnight. Upon completion of the second gas evolution, the produced poly(N-methylaminoborane) was precipitated by the addition of pentane (~50 ml), which was filtered and dried under vacuum overnight yielding a grey solid (55 mg, 59 %). The molecular weight of the polymer produced was investigated by GPC: M_n: 26,800 (Da), M_w: 39,200(Da), PDI: 1.5.

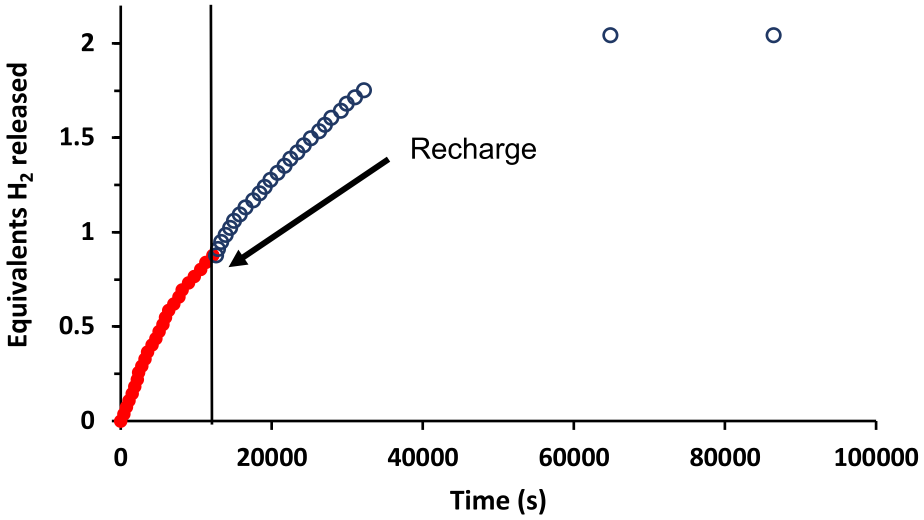

Figure S41. Equivalents of H_2 released over time for the recharge dehydropolymerisation of $H_3B{\cdot}NMeH_2$ (0.446 M) in 1,2-difluorobenzene, by **[6]OTf** (1 mol% cat). Charge 1 (●). Charge 2 started at 13,000 seconds (○)

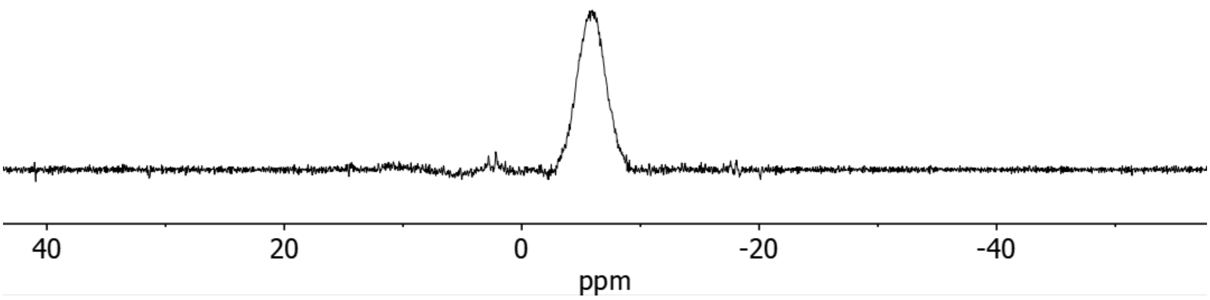

Figure S42. ^{11}B NMR (192 MHz, $CDCl_3$) spectrum of the polymer precipitated by the addition of pentane to the recharge catalysis mixture, $(H_2B{\cdot}NMeH)_n$. Baseline corrected by the subtraction of the borosilicate glass ^{11}B signal.

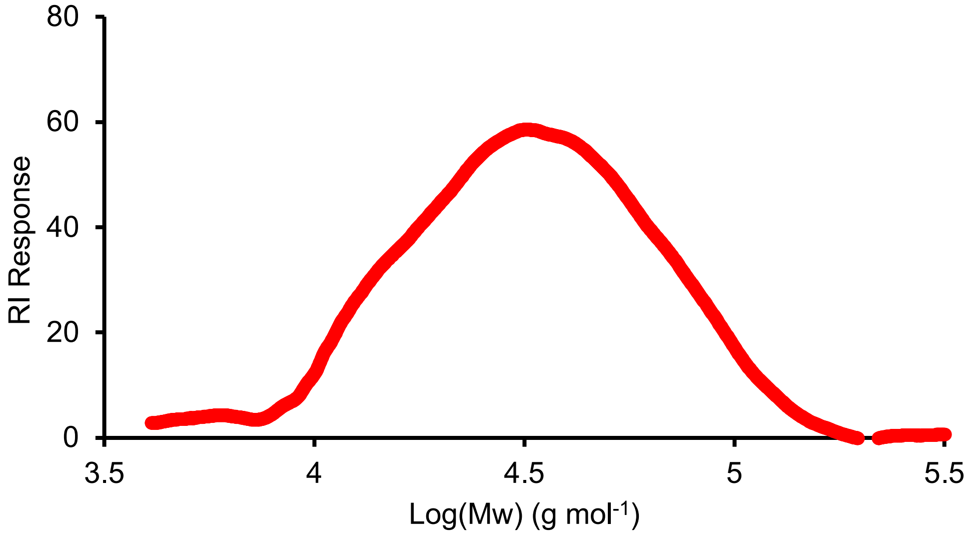

Figure S43. GPC trace of polymer, $(H_2B{\cdot}NMeH)_n$, from the recharge dehydropolymerisation of $H_3B{\cdot}NMeH_2$ with **[6]OTf**.

1.6.5. Dehydropolymerisation of Methylamine-Borane with [6]OTf – Mercury drop test for nanoparticles

$H_3B{\cdot}NMeH_2$ (50.0 mg, 1.11 mmol) was suspended in 1,2−$C_6H_4F_2$ (2.2 mL for 0.446 M [$H_3B{\cdot}NMeH_2$]) in a jacketed three-necked Schlenk flask connected to a recirculating cooler and the temperature set at 25 °C. Precatalyst **[6]OTf** (8.5 mg, 0.011 mmol) was weighed into a separate flask and dissolved in 1,2−$C_6H_4F_2$ (0.3 ml). The $H_3B{\cdot}NMeH_2$-containing flask was sealed off from the Ar supply and connected to a water-filled gas burette. The precatalyst solution was added to the reaction mixture to give loading 1 mol% and the resultant solution was stirred at 400 rpm. The time taken for specified volumes of gas to evolve was recorded. After the evolution of ≈0.25 Eq. H_2 at 1800 s, 2000 Eq. of Hg was added to the flask and the time taken for the evolution of gas continued to be recorded until approx. 1 Eq. of H_2 had evolved. No significant inhibition was observed.

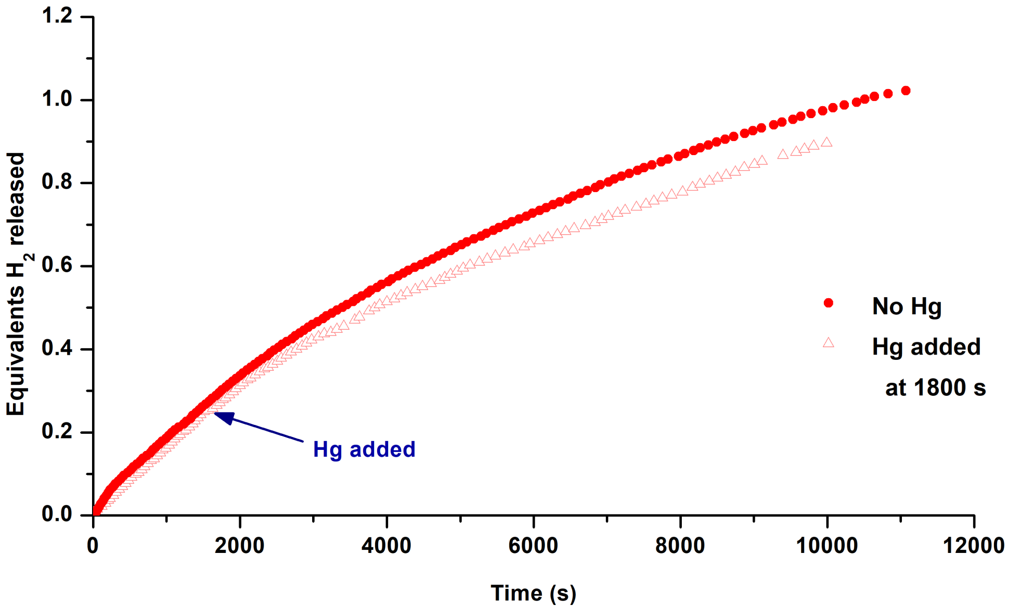

Figure S44. Equivalents of H_2 released over time for dehydropolymerisation of $H_3B·NMeH_2$ (0.446 M) in 1,2-difluorobenzene, by **[6]OTf** (1 mol% cat) with and without added elemental mercury (2000 Eq.), showing no significant inhibition.

1.6.6. Dehydrocoupling of dimethylamine-borane with [6]OTf - Mercury drop test for nanoparticles

$H_3B·NMe_2H$ (65.8 mg, 1.11 mmol) was suspended in 1,2−$C_6H_4F_2$ (2.2 mL for 0.446 M [$H_3B·NMe_2H$]) in a jacketed three-necked Schlenk flask connected to a recirculating cooler and the temperature set at 25 °C. Precatalyst **[6]OTf** (8.5 mg, 0.011 mmol) was weighed into a separate flask and dissolved in 1,2−$C_6H_4F_2$ (0.3 ml). The $H_3B·NMe_2H$-containing flask was sealed off from the Ar supply and connected to a water-filled gas burette. The precatalyst solution was then added to the reaction mixture to give loading 1 mol% and the resultant solution was stirred at 400 rpm. The time taken for specified volumes of gas to evolve was recorded. After the evolution of ≈0.25 Eq. H_2 at 8600 s, 2000 Eq. of Hg was added to the flask and the time taken for the evolution of gas continued to be recorded until approx. 1 Eq. of H_2 had evolved. This was repeated with the Hg added at the start of the reaction. The addition of Hg shuts down the reaction after a short time.

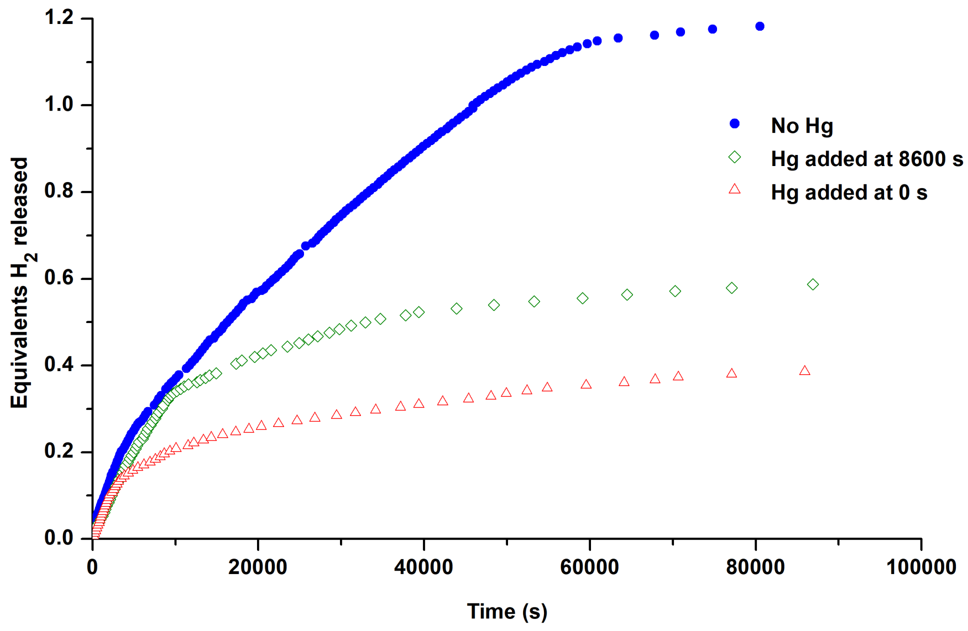

Figure S45. Equivalents of H_2 released over time for dehydrocoupling of $H_3B{\cdot}NMe_2H$ (0.446 M) in 1,2-difluorobenzene, by **[6]OTf** (1 mol% cat) with and without added elemental mercury (2000 Eq.), showing significant catalysis inhibition.

1.6.7. Dehydropolymerisation of methylamine-borane with [6]OTf – PMe_3 poisoning test for heterogenous catalysis

$H_3B{\cdot}NMeH_2$ (50.0 mg, 1.11 mmol) was suspended in 1,2−$C_6H_4F_2$ (2.2 mL for 0.446 M [$H_3B{\cdot}NMeH_2$]) in a jacketed three-necked Schlenk flask connected to a recirculating cooler and the temperature set at 25 °C. Precatalyst **[6]OTf** (8.5 mg, 0.011 mmol) was weighed into a separate flask and dissolved in 1,2−$C_6H_4F_2$ (0.3 ml). The $H_3B{\cdot}NMeH_2$-containing flask was sealed off from the Ar supply and connected to a water-filled gas burette. The precatalyst solution was then added to the reaction mixture to give loading 1 mol% and the resultant solution was stirred at 400 rpm. The time taken for specified volumes of gas to evolve was recorded. After 1950 s, 0.3 Eq. per Rh of PMe_3 was added to the flask and the time taken for the evolution of gas continued to be recorded until approx. 1 Eq. of H_2 had evolved.

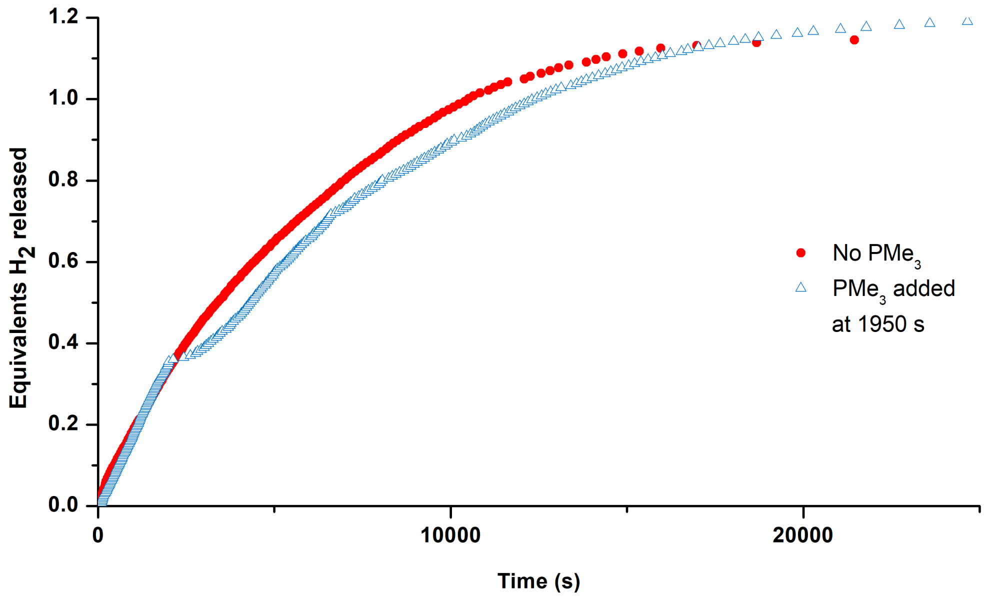

Figure S46. Equivalents of H_2 released over time for dehydropolymerisation of $H_3B{\cdot}NMeH_2$ (0.446 M) in 1,2-difluorobenzene, by **[6]OTf** (1 mol% cat) with and without added PMe_3 (0.3 Eq.), showing brief catalyst inhibition followed by the resumption of catalytic activity after 20 minutes.

1.6.8. Dehydropolymerisation of methylamine-borane with [6]OTf – poisoning with Dibenzo(a,e)cyclooctene (DBCOT) to test for homogenous catalysis

$H_3B{\cdot}NMeH_2$ (50.0 mg, 1.11 mmol) was suspended in 1,2−$C_6H_4F_2$ (2.2 mL for 0.446 M [$H_3B{\cdot}NMeH_2$]) in a jacketed three-necked Schlenk flask connected to a recirculating cooler and the temperature set at 25 °C. Precatalyst **[6]OTf** (8.5 mg, 0.011 mmol) was weighed into a separate flask and dissolved in 1,2−$C_6H_4F_2$ (0.3 ml). The $H_3B{\cdot}NMeH_2$-containing flask was sealed off from the Ar supply and connected to a water-filled gas burette. The precatalyst solution was then added to the reaction mixture to give loading 1 mol% and the resultant solution was stirred at 400 rpm. The time taken for specified volumes of gas to evolve was recorded. After 1800 s, 1 Eq. per Rh of dibenzo(a,e)cyclooctene (DBCOT) dissolved in 1,2-$C_6H_4F_2$ (0.2 ml) was added to the flask by syringe and the time taken for the evolution of gas continued to be recorded until approx. 1 Eq. of H_2 had evolved.

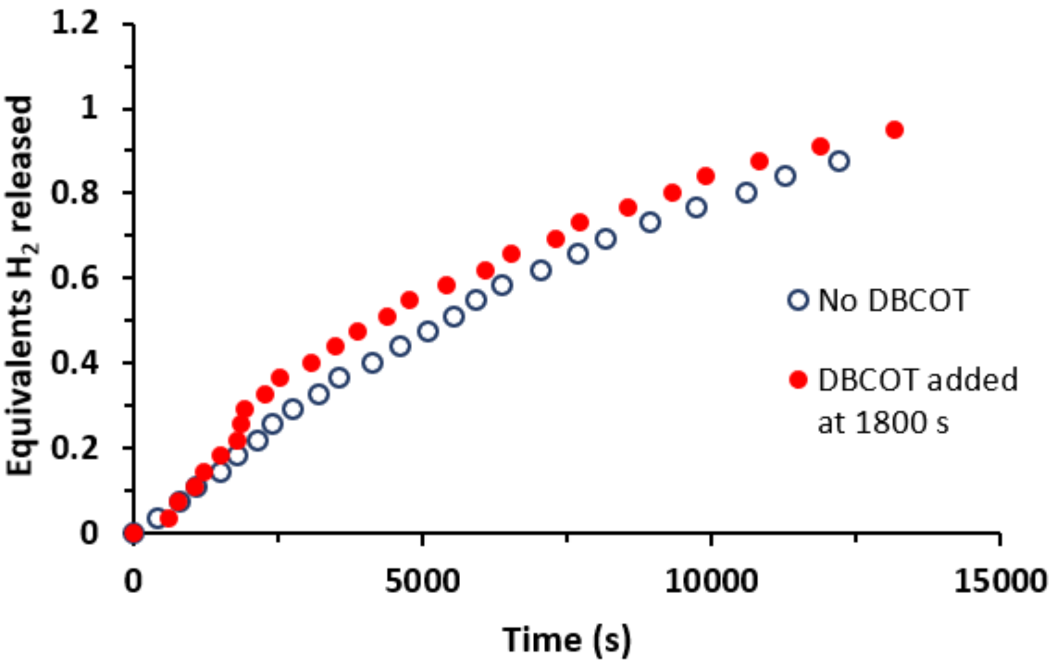

Figure S47. Equivalents of H_2 released over time for dehydropolymerisation of $H_3B{\cdot}NMeH_2$ (0.446 M) in 1,2-difluorobenzene, by **[6]OTf** (1 mol% cat) with and without dibenzo(a,e)cyclooctene (DBCOT, 1 Eq., dissolved in 0.2 ml of 1,2-$C_6H_4F_2$ and added by syringe), showing no inhibition of catalytic activity. The small increase in volume at 1800s is ascribed to the injection of DBCOT at this point and the associated perturbation of the eudiometric measurements.

1.6.9. Dehydropolymerisation of methylamine-borane with [6]OTf – Recharge experiment with filtration, test for homogenous and heterogenous catalysis

$H_3B{\cdot}NMeH_2$ (50.0 mg, 1.11 mmol) was suspended in 1,2−C_6H_4 F_2 (2.2 mL for 0.446 M [$H_3B{\cdot}NMeH_2$]) in a jacketed three-necked Schlenk flask connected to a recirculating cooler and the temperature set at 25 °C. Precatalyst **[6]OTf** (8.5 mg, 0.011 mmol) was weighed into a separate flask and dissolved in 1,2−$C_6H_4F_2$ (0.3 ml). The $H_3B{\cdot}NMeH_2$-containing flask was sealed off from the Ar supply and connected to a water-filled gas burette. The precatalyst solution was added to the reaction mixture to give loading 1 mol% and the resultant solution was stirred at 400 rpm. The time taken for specified volumes of gas to evolve was recorded. Upon completion of gas evolution (after evolution of ≈ 1 Eq. H_2), the reaction solution was filtered through a 0.2 μm PTFE syringe filter in a glove box and placed into a second jacketed three-necked Schlenk flask. The flask was connected to a water-filled gas burette and additional $H_3B{\cdot}NMeH_2$ (50.0 mg, 1.11 mmol) added. The time taken for specified volumes of gas to evolve was recorded. This procedure was repeated twice.

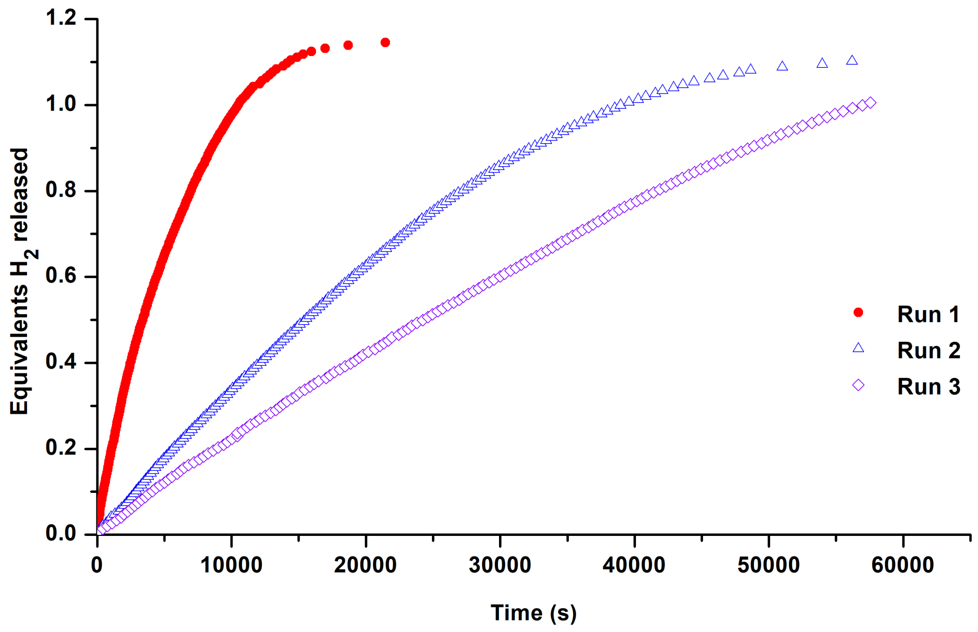

Figure S48. Equivalents of H_2 released over time for dehydropolymerisation of $H_3B{\cdot}NMeH_2$ (0.446 M) in 1,2-difluorobenzene, by **[6]OTf** (1 mol% cat). Recharged with filtration of the reaction mixture between charges, showing decreasing reaction rate with subsequent filtrations.

1.6.10. Large-scale dehydropolymerisation of methylamine-borane with [6]OTf and polymer purification

$H_3B{\cdot}NMeH_2$ (2.0 g, 44 mmol) and **[6]OTf** (8.5 mg, 0.011 mmol, 0.025 mol%) were placed in a jacketed three-neck Schlenk flask under air, connected to a recirculating cooler and the temperature set at 25 °C. THF (5 ml) was added. The solution was stirred for 48 h and the reaction monitored by NMR. The solution was decanted into pentane (30 ml) to precipitate the polymer as a grey solid. The solid was collected and vacuum dried overnight (1.839 g). The molecular weight of the polymer produced was investigated by GPC: M_n: 30,900 (Da), M_w: 54,400 (Da), PDI: 1.76. The residual metal content was analysed by ICP-MS and found to be 532 μg/g of Rhodium.

1 g of the crude polymer was dissolved in THF (20 ml) and 1 g of activated carbon added and the mixture stirred for 30 min. The mixture was then centrifuged (5 min, 8000 rpm) to remove the carbon. The supernatant was passed through a 0.2 μm PTFE syringe filter to remove any residual carbon and the resultant colourless solution concentrated under vacuum to approx. 1 ml. Pentane (20 ml) was added to precipitate a white solid which was collected and vacuum dried overnight (0.875 g). The molecular weight of the polymer produced was investigated by GPC: M_n: 30,500 (Da), M_w: 51,200 (Da), PDI: 1.68. The residual metal content was analysed by ICP-MS and found to be 6 μg/g of Rhodium.

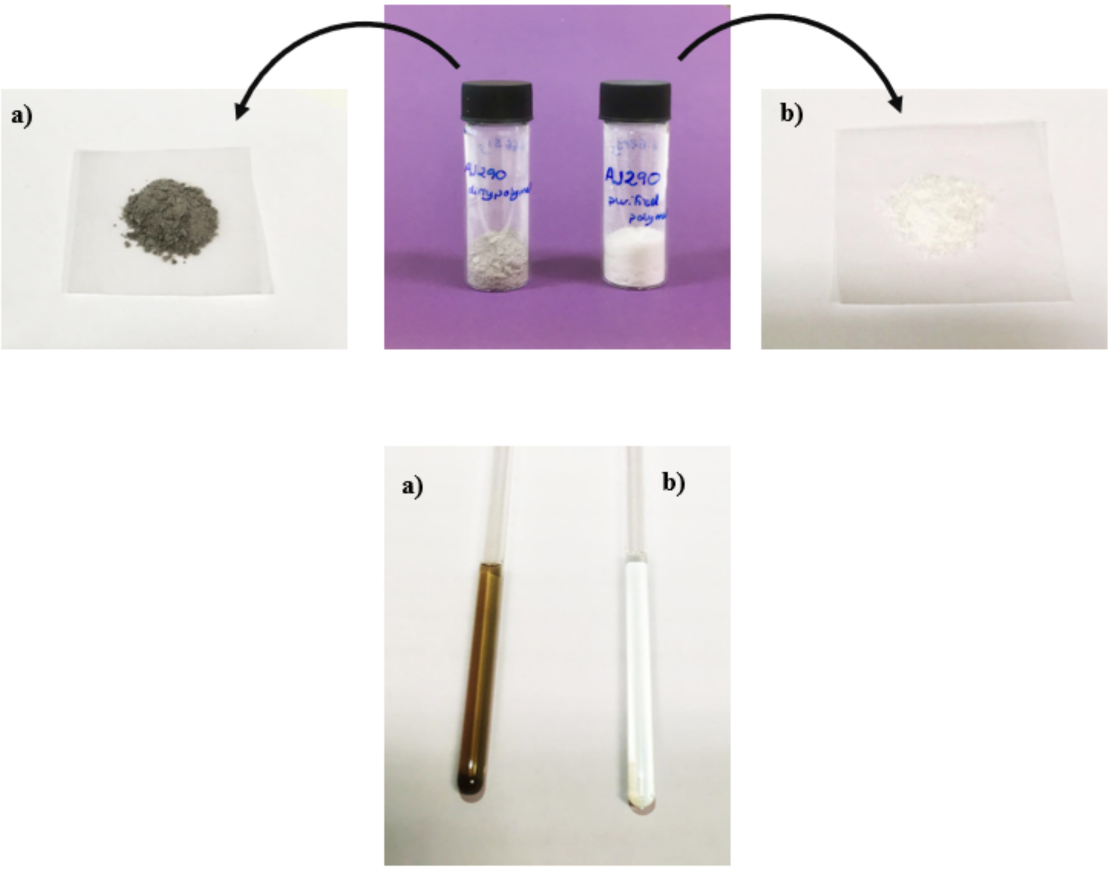

Figure S49. Photos showing (a) crude poly(N-methylaminoborane) and (b) purified poly(N-methylaminoborane) as a solid and as NMR solutions in THF.

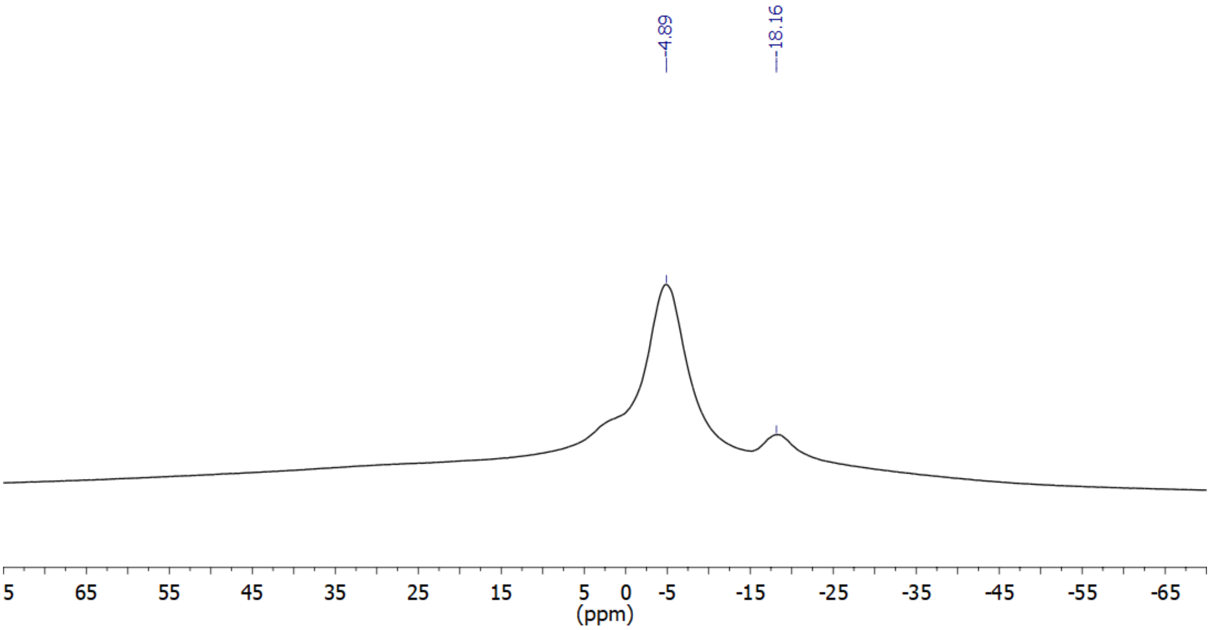

Figure S50. ^{11}B NMR (128 MHz, THF) of the crude poly(N-methylaminoborane) after precipitation from the reaction mixture with pentane.

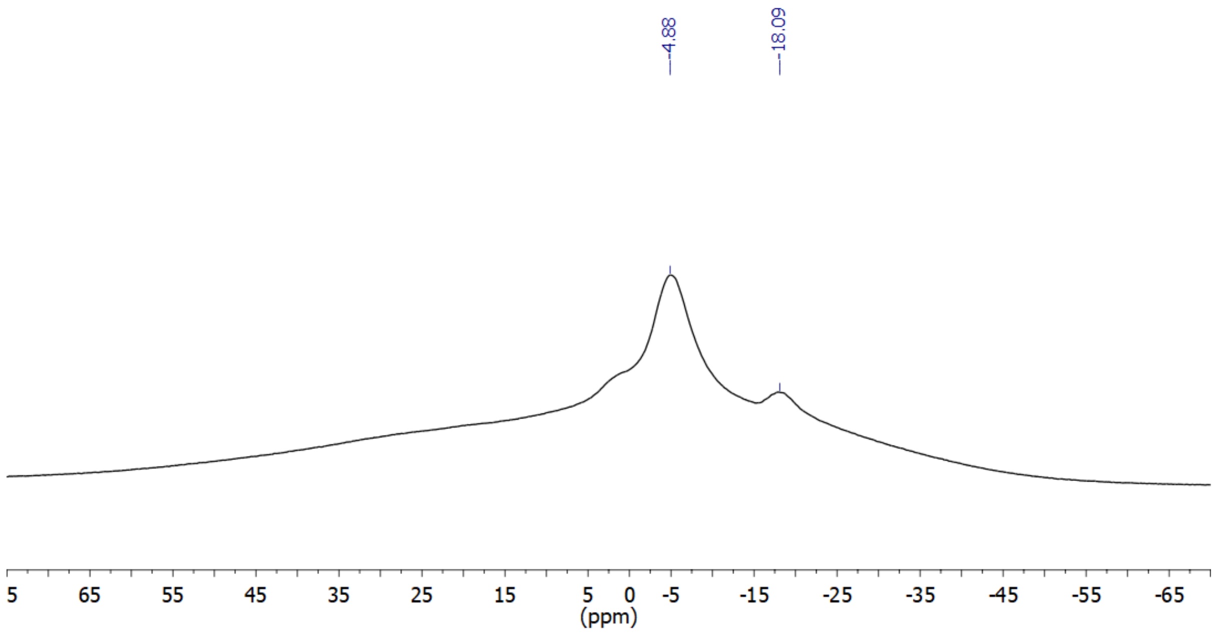

Figure S51. ^{11}B NMR (128 MHz, THF) of the poly(N-methylaminoborane) purified by stirring with activated charcoal and filtration followed by precipitation from THF with pentane.

1.6.11. Depolymerisation of poly(N-methylaminoborane) with catalytic NaHMDS

Activated-charcoal-treated Poly(N-methylaminoborane), $((H_2B{\cdot}NMeH)_n$, 10 mg, 0.023 mmol approximating as the monomer unit $H_2B{=}NMeH$) and sodium hexamethyldisilazide (NaHMDS, 4 mg, 0.023 mmol, 10 mol%) were dissolved in THF (1 ml) and stirred for 3 hours (400 rpm, 298 K). Poly(N-methylaminoborane) obtained from the dehydropolymerisation of methylamine-borane by **[6]OTf** (Mn = 20,500 g mol^{-1}). *In-situ* NMR spectra were recorded (^1H, ^{11}B) of a sample of the reaction mixture in THF. The NMR sample was then evaporated to dryness and the solids re-dissolved in $CDCl_3$, upon which more NMR spectra were recorded (^1H, ^{11}B, $^{11}B\{^1H\}$ & $^{13}C\{1H\}$). 74% conversion of the polymer to 1,3,5-*N*,*N*,*N*-trimethylborazane was observed by ^{11}B NMR. Only one isomer of 1,3,5-*N*,*N*,*N*-trimethylborazane (*e*,*e*,*e*) was observed ($^{13}C\{^1H\}$ and $^{11}B\{^1H\}$ NMR.

Depolymerisation was also attempted with activated-charcoal-treated poly(N-methylaminoborane) from the $[Rh(^iPr\text{-}PN^HP)(NBD)]Cl$.[10] Poly(N-methylaminoborane) produced by this catalyst system (Mn = 65,200 g mol^{-1}) required only 2.5 mol% NaHMDS for complete depolymerisation to 1,3,5-*N*,*N*,*N*-trimethylborazane (>90% selectivity).

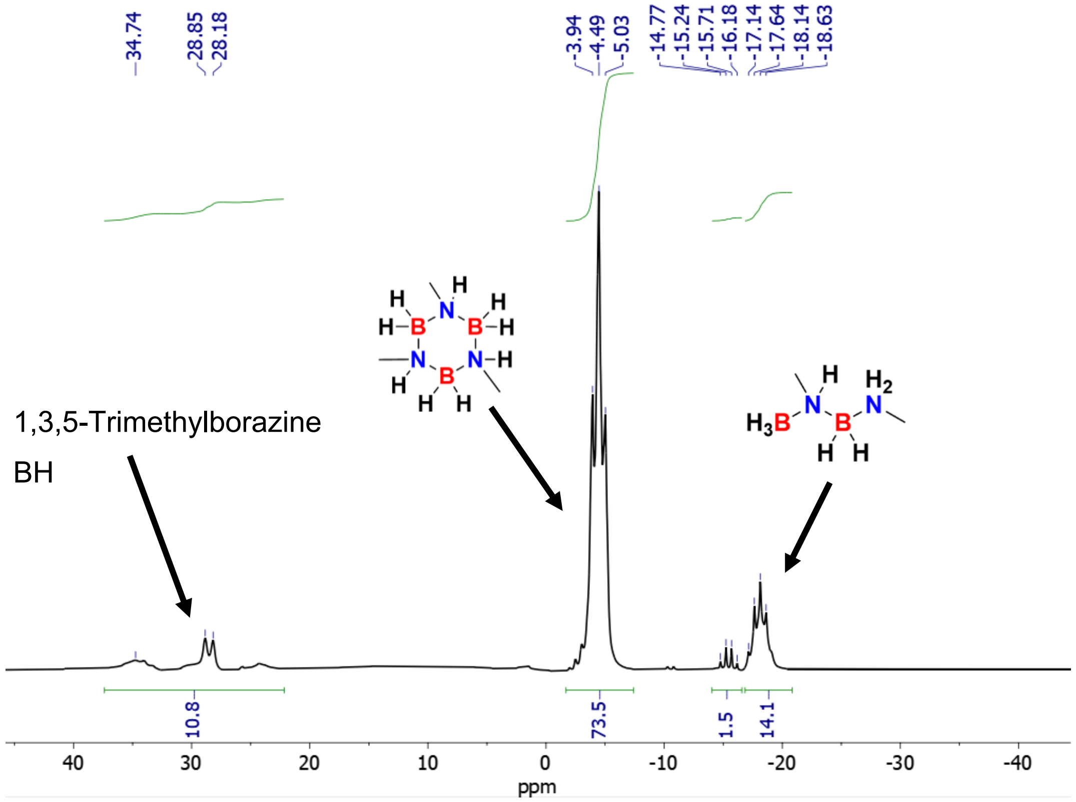

Figure S52. ^{11}B NMR (192 MHz, THF) *In-situ* of depolymerisation reaction mixture after 3 hours showing 1,3,5-*N*,*N*,*N*-trimethylborazane and other BN side products (polymer from **[6]OTf**). Baseline corrected by the subtraction of the borosilicate glass ^{11}B signal.

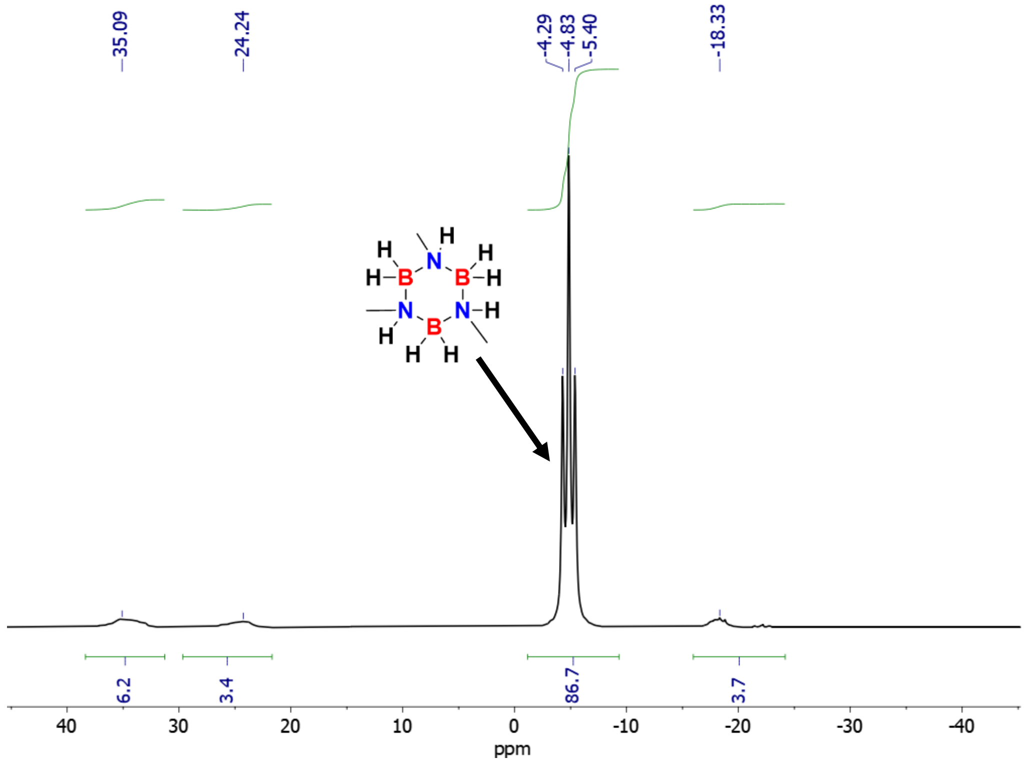

Figure S53. ^{11}B NMR (192 MHz, $CDCl_3$) Spectrum of depolymerisation reaction products showing 1,3,5-*N*,*N*,*N*-trimethylborazane and small amounts of other BN side products (polymer from **[6]OTf**). Baseline corrected by the subtraction of the borosilicate glass ^{11}B signal.

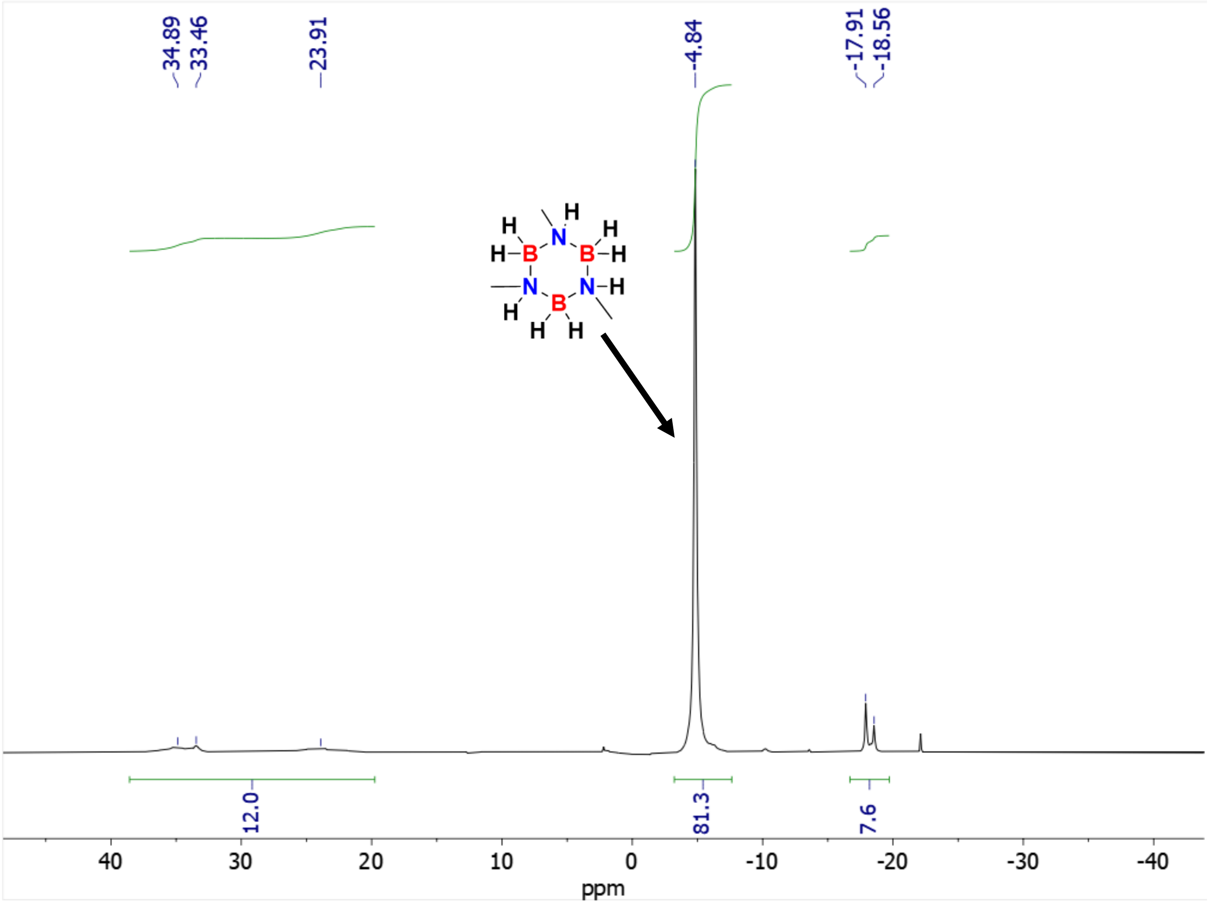

Figure S54. $^{11}B\{^1H\}$ NMR (192 MHz, $CDCl_3$) Spectrum of depolymerisation reaction products showing one isomer of 1,3,5-*N*,*N*,*N*-trimethylborazane and small amounts of other BN side products (polymer from **[6]OTf**). Baseline corrected by the subtraction of the borosilicate glass ^{11}B signal.

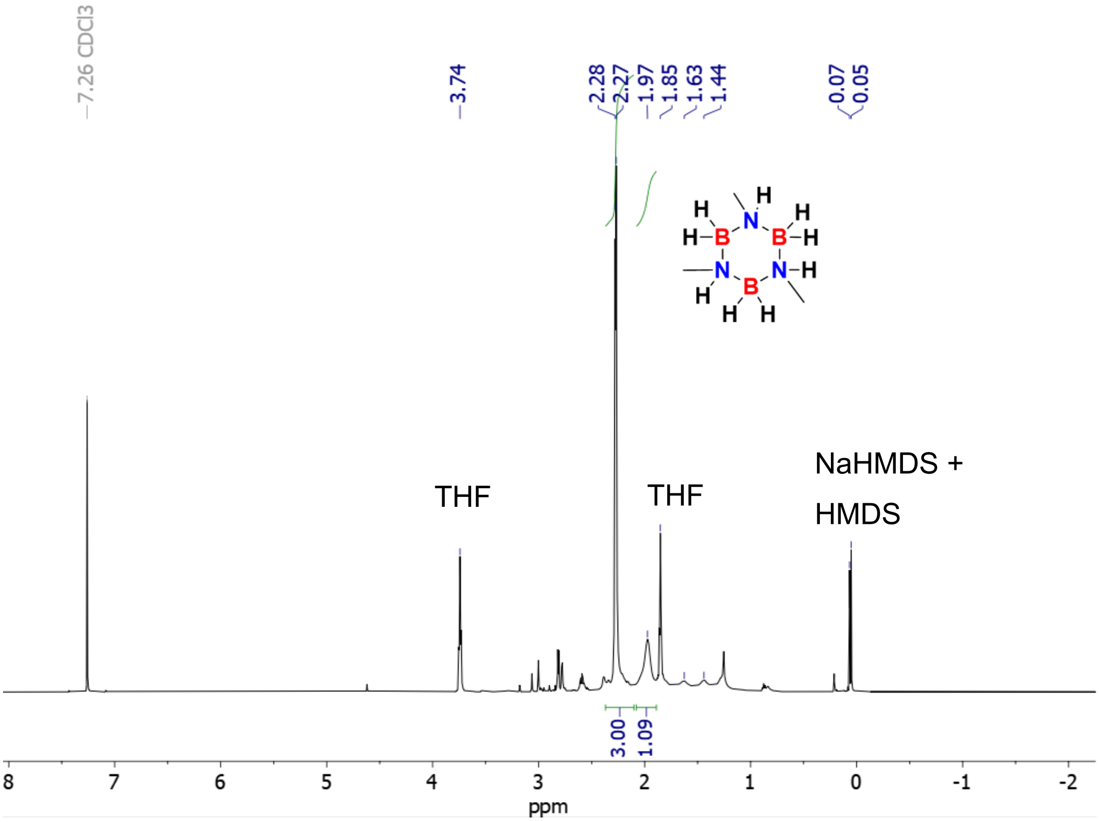

Figure S55. ^{1}H NMR (600 MHz, $CDCl_3$) Spectrum of depolymerisation reaction products showing 1,3,5-*N*,*N*,*N*-trimethylborazane. δ 2.27 (d, 5.6 Hz, 9H, CH_3), 1.97 (s, br, 3H, NH). Broad triplet, 113 Hz centred around 1.6 ppm corresponds to BH_2. Polymer from **[6]OTf.**

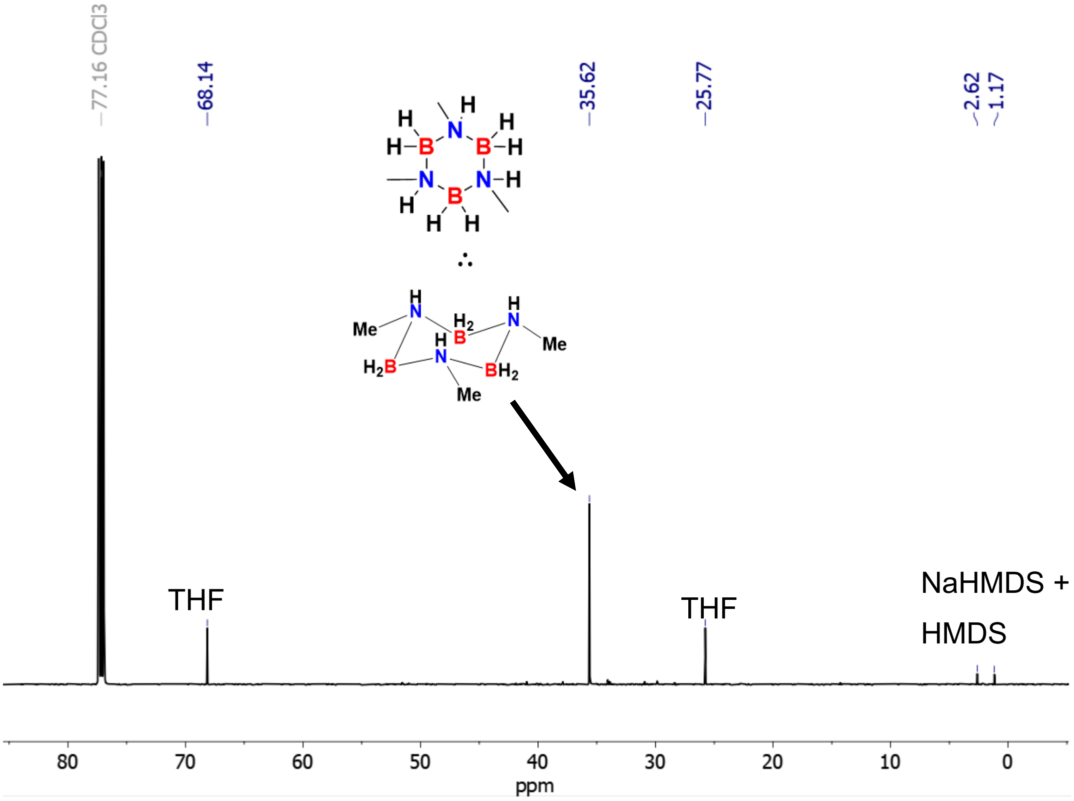

Figure S56. ^{13}C{^{1}H} NMR (151 MHz, $CDCl_3$) Spectrum of depolymerisation reaction products showing one isomer (*e*,*e*,*e*) of 1,3,5-*N*,*N*,*N*-trimethylborazane. Polymer from **[6]OTf**.

1.6.12. Dehydropolymerisation of Ethylamine-Borane with [6]OTf - Pre-activated catalyst

[6]OTf (8.5 mg, 0.011 mmol) and $H_3B{\cdot}NMeH_2$ (1.5 mg, 0.033 mmol) were dissolved in 1,2-$C_6H_4F_2$ (2.5 ml) then $EtNH_2$ (50 µl, 0.11 mmol, 2.0 M THF) was added and the reaction mixture was stirred at 800 rpm for 15 minutes. After 15 minutes the dark-green solution became dark-red, forming the pre-activated catalyst solution. $H_3B{\cdot}NEtH_2$ (66 mg, 1.11 mmol) was added to a jacketed three-neck Schlenk flask connected to a recirculating cooler and the temperature set at 25 °C. The jacketed Schlenk was then sealed off from the Ar supply and connected to a water-filled gas burette. The pre-activated catalyst solution was then transferred into the jacketed Schlenk (1 mol% catalyst) and the resulting solution was stirred at 400 rpm. The total volume of 1,2-$C_6H_4F_2$ was 2.5 ml, giving a $H_3B{\cdot}NEtH_2$ concentration of 0.446 M. The time taken for H_2 gas to be evolved was recorded. Upon completion of gas evolution, the reaction mixture solvent was partially removed in-vacuo, then the produced poly(N-ethylaminoborane) was precipitated by the addition of pentane (~50 ml), which was filtered and dried under vacuum overnight yielding a grey solid (5 mg, 8%). The molecular weight of the polymer produced was investigated by GPC: M_n: 35,500 (Da), M_w: 41,200 (Da), PDI: 1.3.

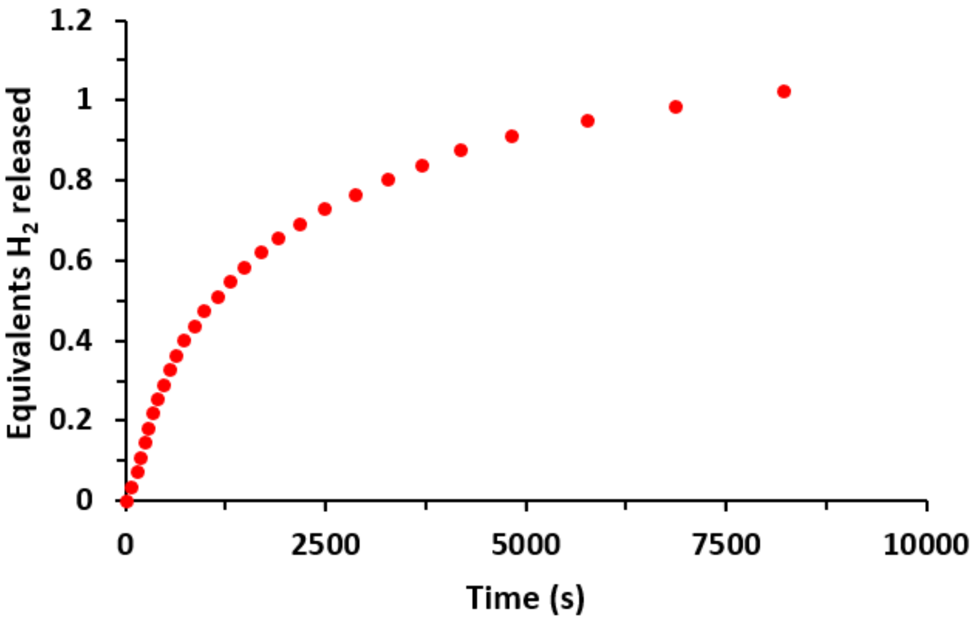

Figure S57. Equivalents of H_2 released over time for dehydropolymerisation of $H_3B{\cdot}NEtH_2$ (0.446 M) in 1,2-difluorobenzene, with **[6]OTf** (1 mol% cat) and $EtNH_2$.

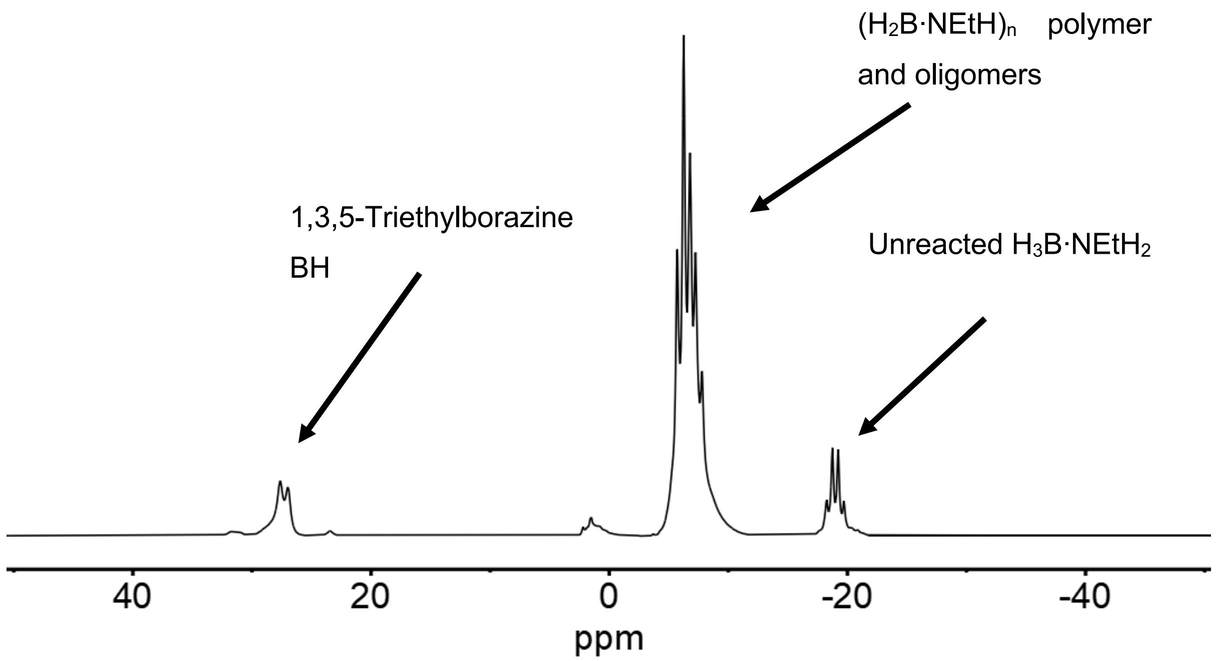

Figure S58. ^{11}B NMR (192 MHz, 1,2-$C_6H_4F_2$) *In-situ* of catalysis reaction mixture after 10,000 seconds showing starting material ($H_3B{\cdot}NEtH_2$), polymer $(H_2B{\cdot}NEtH)_n$ and other BN side products. Baseline corrected by the subtraction of the borosilicate glass [11]B signal.

1.6.13. Dehydropolymerisation of *n*-Propylamine-Borane with [6]OTf - Pre-activated catalyst

[6]OTf (8.5 mg, 0.011 mmol and $H_3B{\cdot}N^nPrH_2$ (1.5 mg, 0.033 mmol) were dissolved in 1,2-$C_6H_4F_2$ (2.5 ml) then $^nPrNH_2$ (10 µl, 0.11 mmol, neat) was added and the reaction mixture was stirred at 800 rpm for 15 minutes. After 15 minutes the dark-green solution became dark-red, forming the pre-activated catalyst solution. $H_3B{\cdot}N^nPrH_2$ (82 mg, 1.11 mmol) was added to a jacketed three-necked Schlenk flask connected to a recirculating cooler and the temperature set at 25 °C. The jacketed Schlenk was then sealed off from the Ar supply and connected to a water-filled gas burette. The pre-activated catalyst solution was then transferred into the jacketed Schlenk (1 mol% catalyst) and the resulting solution was stirred at 400 rpm. The total volume of 1,2-$C_6H_4F_2$ was 2.5 ml, giving a $H_3B{\cdot}N^nPrH_2$ concentration of 0.446 M. The time taken for H_2 gas to be evolved was recorded. Upon completion of gas evolution, the reaction mixture solvent was partially removed in-vacuo, then the produced poly(N-propylaminoborane) was precipitated by the addition of pentane (~50 ml), which was filtered and dried under vacuum overnight yielding a grey solid (15 mg, 24%). The molecular weight of the polymer produced was investigated by GPC: M_n: 23,400 (Da), M_w: 31,800 (Da), PDI: 1.4.

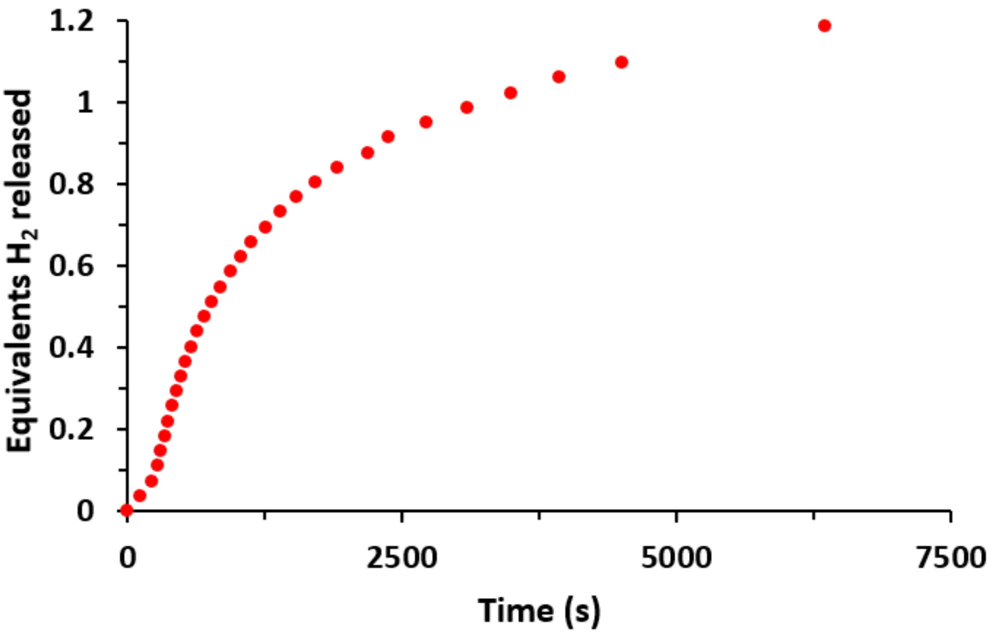

Figure S59. Equivalents of H_2 released over time for dehydropolymerisation of $H_3B{\cdot}N^nPrH_2$ (0.446 M) in 1,2-difluorobenzene, with **[6]OTf** (1 mol% cat) and $^nPrNH_2$.

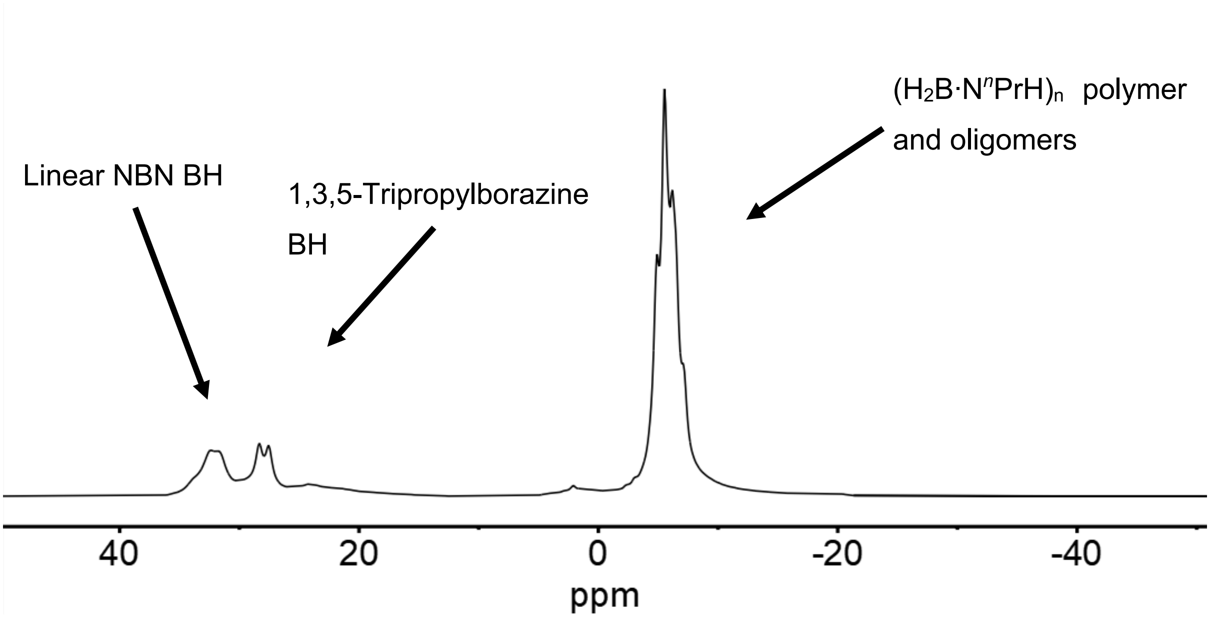

Figure 60. ^{11}B NMR (192 MHz, 1,2-$C_6H_4F_2$) *In-situ* of catalysis reaction mixture after 8000 seconds showing polymer $(H_2B{\cdot}N^nPrH)_n$ and other BN side products. Baseline corrected by the subtraction of the borosilicate glass ^{11}B signal.

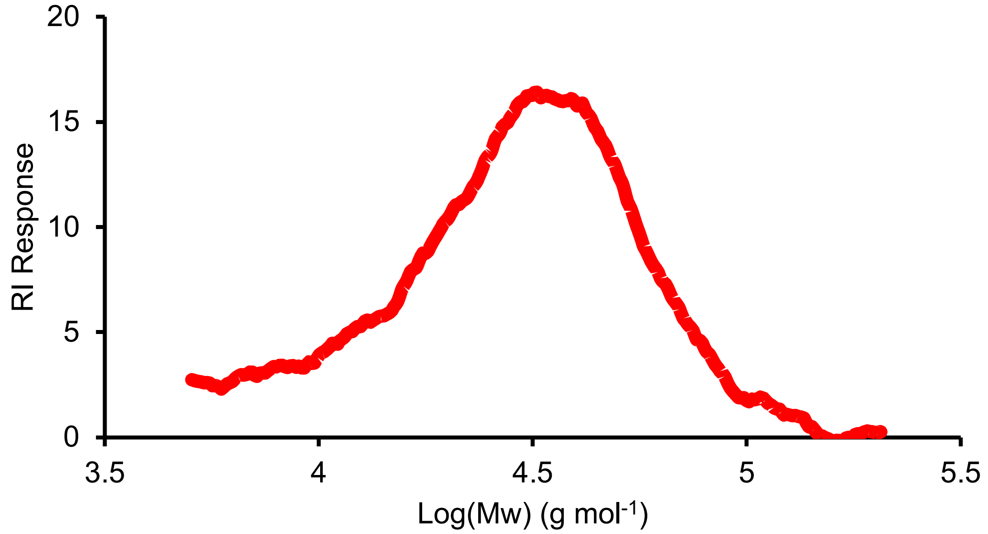

Figure S61. GPC trace of polymer, $(H_2B{\cdot}N^nPrH)_n$, from the dehydropolymerisation of $H_3B{\cdot}N^nPrH_2$ with **[6]OTf** in the presence of excess $^nPrNH_2$.

1.7. TEM Rh-nanoparticle images

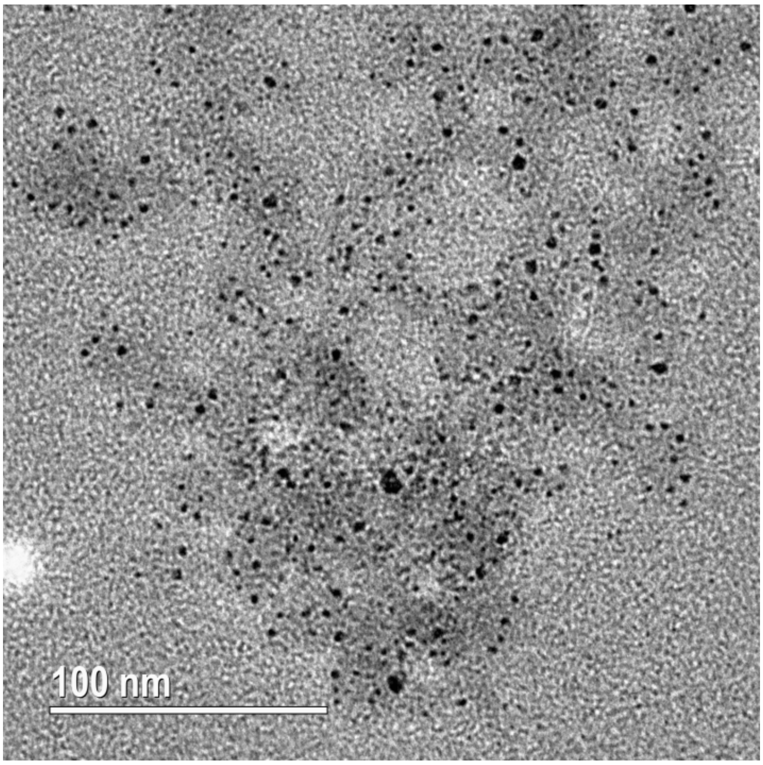

Figure S62. TEM image of crude poly(N-methylaminoborane) containing polymer-entrained Rh nanoparticles

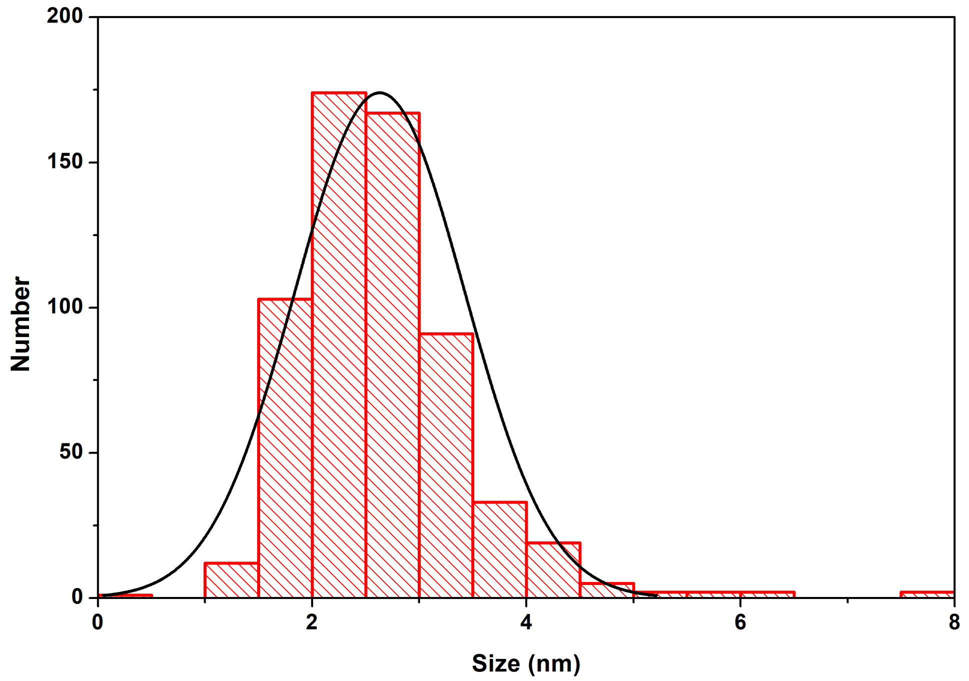

Figure S63. Size distribution histogram of 617 nanoparticles from TEM images of Crude poly(N-methylaminoborane).

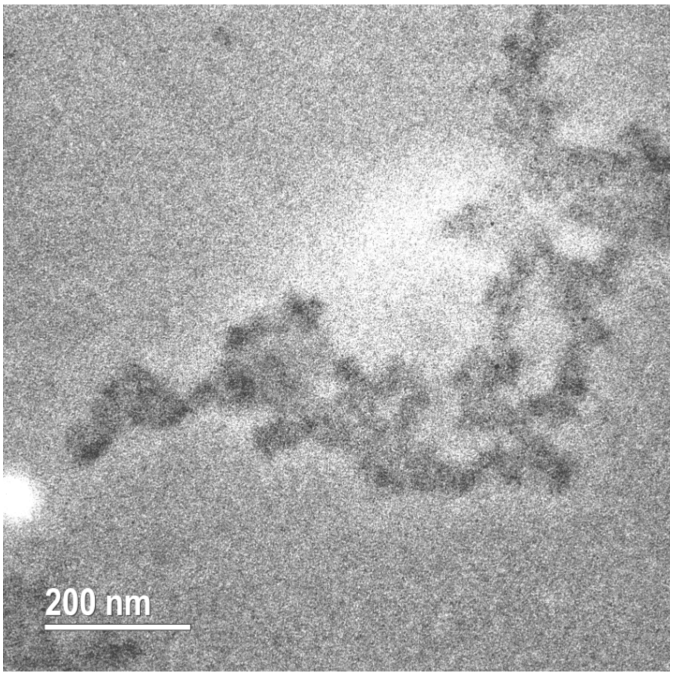

Figure S64. TEM image of poly(N-methylaminoborane) post-purification by stirring with charcoal and filtration through a 0.2 μm PTFE filter.

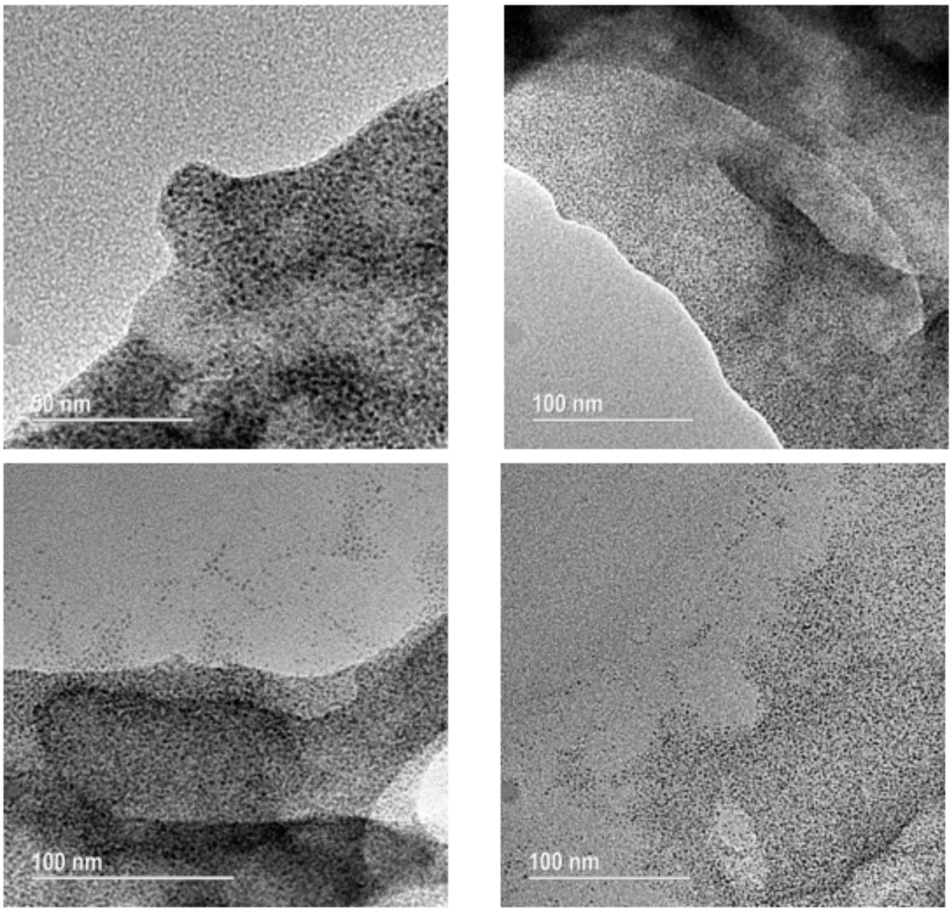

Figure S65. TEM images of the 0.2 μm PTFE filter used for polymer purification.

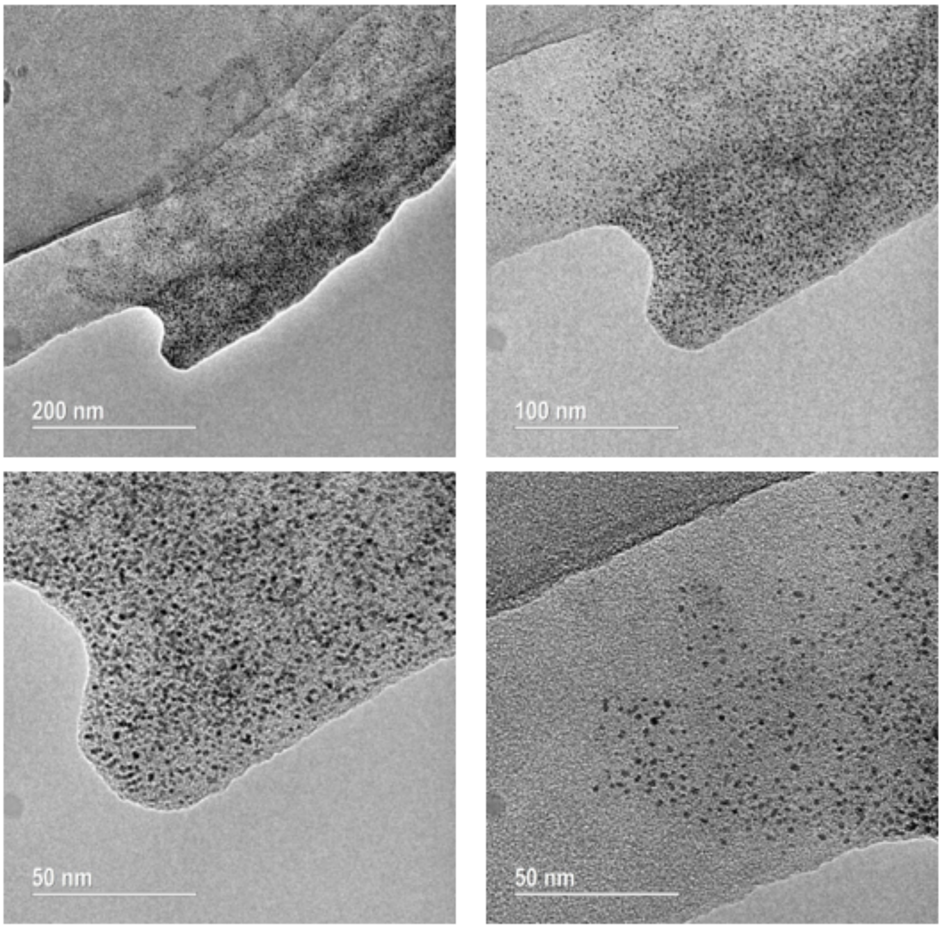

Figure S66. TEM images of the 0.2 μm PTFE filters used for reaction mixture filtration during the filtered recharge dehydropolymerisation (see **1.6.8.**).

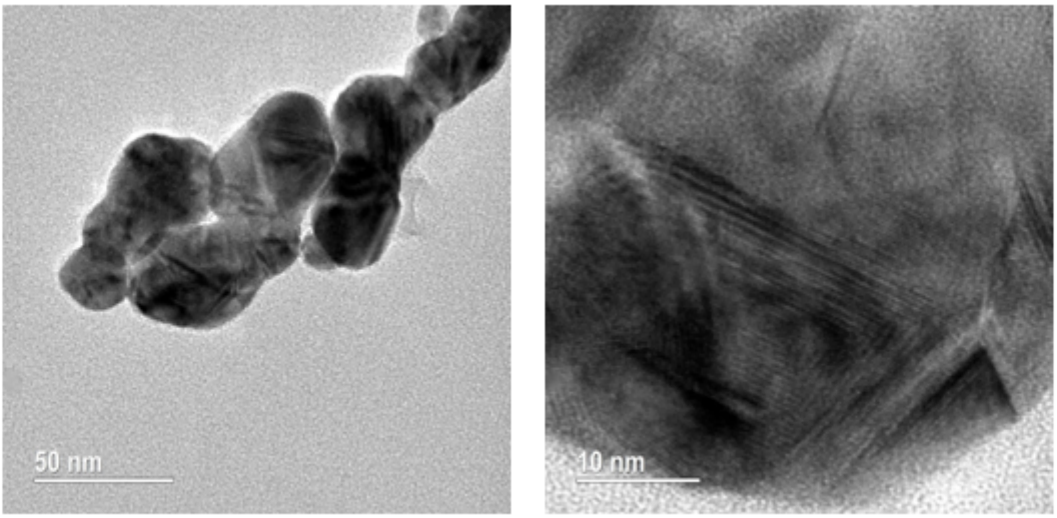

Figure S67. TEM images of crystalline precatalyst **[6]OTf**, samples prepared in a glovebox (as used in catalysis).

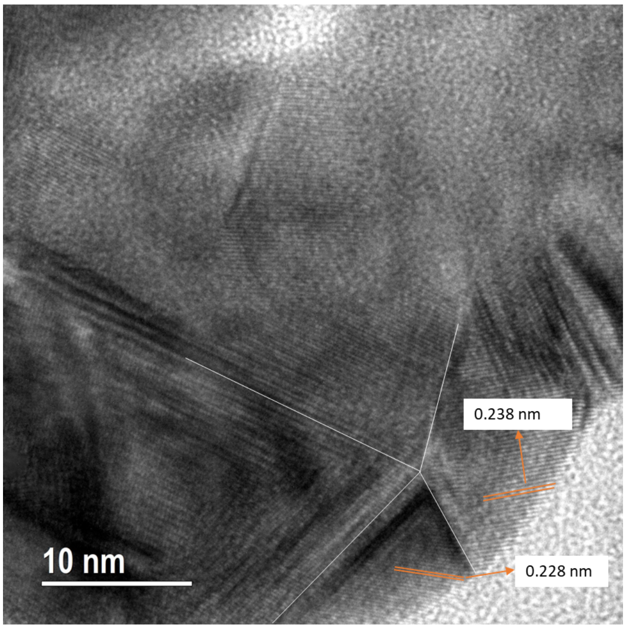

Figure S68. TEM image of crystalline precatalyst **[6]OTf** prepared under inert atmosphere showing lattice fringes of ~0.23 nm, assigned to the (111) plane of fcc Rh. This suggests beam degradation of **[6]OTf**.[11–13]

1.8. Computational Calculations using Density Functional Theory (DFT) Methods

All calculations were performed using the TURBOMOLE V6.4 package using the resolution of identity (RI)[14–21] approximation. Initial optimisations were performed at the (RI-)BP86/SV(P) level of theory, followed by vibrational frequency calculations at the same level. All minima were confirmed by the absence of imaginary vibrational frequencies. Single-point energies were calculated on the (RI-)BP86/SV(P) optimised geometries using the PBE0 hybrid functional and def2-TZVPP basis set. The energies were corrected in post for dispersion with Grimme's D3 method with BJ dampening.[22,23] Solvation effects were modelled using COSMO,[24] with the dielectric constant of 7.58 for tetrahydrofuran.

1.8.1. DFT Optimised *xyz* Coordinates and Collated Energies of 1e,3e,5a-trimethylborazine

SCF Energy (au) BP86/SV(P)	-363.7842376644
SCF Energy (au) PBE0/def2-TZVPP	-363.7676843835
SCF Energy (au) PBE0/def2-TZVPP COSMO THF	-363.7839955087
Zero Point Energy (au)	0.2360701
Chemical potential (kJ mol^{-1})	527.72
Dispersion correction (au) PBE0/def2-TZVPP	-0.02405903

xyz coordinates

```
27
B     1.5501450   -0.0581654   -0.2222046
H     2.1764366   -0.0477190   -1.2881937
H     2.2819495   -0.0454538    0.7756834
N     0.5924290    1.2240586   -0.1765248
C     1.2872591    2.4192455   -0.7154248
H     0.4076782    1.4117730    0.8233027
B    -0.8082334    0.9908900   -0.8890866
N    -1.6104489   -0.1096983   -0.0335169
H    -0.5987393    0.5378997   -2.0217800
H     2.2834872    2.5329971   -0.2362168
H     0.6768297    3.3314539   -0.5438907
H     1.4316108    2.2843065   -1.8057321
N     0.6392063   -1.3740095   -0.1699096
H    -1.4557730    2.0446922   -0.9116035
B    -0.6766641   -1.1929818    0.7010728
C     1.4318397   -2.5432990    0.2849519
H     0.3512717   -1.5689885   -1.1437920
C    -2.5838706    0.4648167    0.9321646
H    -2.1664271   -0.6278009   -0.7307729
H    -1.2933819   -2.2648594    0.7768963
H    -0.3488289   -0.7532186    1.8096245
H     2.3700153   -2.6234806   -0.3049097
H     0.8380911   -3.4768687    0.1824354
H     1.6943579   -2.4006568    1.3520262
H    -3.3392532    1.0803373    0.3999762
H    -2.0437363    1.1110230    1.6533005
H    -3.0872503   -0.3462930    1.5021242

$vibrational spectrum
#  mode     symmetry     wave number   IR intensity    selection rules
#                         cm**(-1)        km/mol         IR     RAMAN
```

1		0.00	0.00000	-	-
2		0.00	0.00000	-	-
3		0.00	0.00000	-	-
4		0.00	0.00000	-	-
5		0.00	0.00000	-	-
6		0.00	0.00000	-	-
7	a	85.18	5.94762	YES	YES
8	a	120.28	6.60924	YES	YES
9	a	143.53	3.95710	YES	YES
10	a	233.79	3.35625	YES	YES
11	a	235.66	1.68604	YES	YES
12	a	243.96	0.29904	YES	YES
13	a	253.09	0.99845	YES	YES
14	a	277.83	0.41289	YES	YES
15	a	325.19	18.98568	YES	YES
16	a	336.09	6.54242	YES	YES
17	a	354.08	1.13888	YES	YES
18	a	384.39	0.41484	YES	YES
19	a	397.57	3.11084	YES	YES
20	a	419.09	6.36710	YES	YES
21	a	484.27	0.78799	YES	YES
22	a	705.55	0.93015	YES	YES
23	a	734.86	2.75461	YES	YES
24	a	754.98	0.25400	YES	YES
25	a	778.13	6.26990	YES	YES
26	a	831.64	5.75226	YES	YES
27	a	832.92	7.21244	YES	YES
28	a	875.27	23.14353	YES	YES
29	a	888.52	30.38809	YES	YES
30	a	906.40	26.50888	YES	YES
31	a	943.91	7.93980	YES	YES
32	a	969.08	11.69718	YES	YES
33	a	999.57	0.57641	YES	YES
34	a	1043.36	22.27182	YES	YES
35	a	1049.03	8.09990	YES	YES
36	a	1063.00	18.35067	YES	YES
37	a	1069.63	2.27569	YES	YES
38	a	1092.03	12.97002	YES	YES
39	a	1110.39	5.90578	YES	YES
40	a	1116.69	1.70098	YES	YES
41	a	1124.85	30.84501	YES	YES
42	a	1130.36	8.37730	YES	YES
43	a	1138.89	4.73013	YES	YES
44	a	1156.07	97.44408	YES	YES
45	a	1159.13	119.38209	YES	YES
46	a	1167.46	33.62771	YES	YES
47	a	1175.00	43.65584	YES	YES
48	a	1178.40	139.07734	YES	YES
49	a	1206.12	37.61115	YES	YES
50	a	1222.25	52.22768	YES	YES
51	a	1230.35	25.84563	YES	YES
52	a	1360.92	39.04117	YES	YES
53	a	1363.01	51.63075	YES	YES
54	a	1382.53	11.92959	YES	YES
55	a	1400.49	3.64412	YES	YES
56	a	1401.04	3.24109	YES	YES
57	a	1405.59	3.80579	YES	YES
58	a	1436.15	9.57116	YES	YES
59	a	1440.46	9.21494	YES	YES
60	a	1440.89	18.56759	YES	YES
61	a	1443.32	8.95113	YES	YES
62	a	1443.78	22.08639	YES	YES
63	a	1449.39	17.87615	YES	YES
64	a	2388.97	210.47255	YES	YES
65	a	2392.22	161.17399	YES	YES
66	a	2407.10	35.43727	YES	YES

67	a	2438.01	87.46954	YES	YES
68	a	2439.00	186.40453	YES	YES
69	a	2450.48	278.58597	YES	YES
70	a	2958.60	36.61394	YES	YES
71	a	2959.72	58.78433	YES	YES
72	a	2960.35	8.75433	YES	YES
73	a	3044.30	10.25700	YES	YES
74	a	3044.69	18.20597	YES	YES
75	a	3046.18	16.59167	YES	YES
76	a	3066.04	13.42955	YES	YES
77	a	3072.62	12.30473	YES	YES
78	a	3072.71	1.30567	YES	YES
79	a	3309.65	7.59971	YES	YES
80	a	3310.29	0.55128	YES	YES
81	a	3346.48	10.11881	YES	YES

$end

1.8.2. DFT Optimised *xyz* Coordinates and Collated Energies of 1e,3e,5e-trimethylborazine

SCF Energy (au) BP86/SV(P)	-363.7837545770
SCF Energy (au) PBE0/def2-TZVPP	-363.7676924197
SCF Energy (au) PBE0/def2-TZVPP COSMO THF	-363.7885484710
Zero Point Energy (au)	0.2358706
Chemical potential (kJ mol^{-1})	527.52
Dispersion correction (au) PBE0/def2-TZVPP	-0.02367427

xyz coordinates

```
27
B     1.5615935    0.0068335    0.0674242
H     2.6493653    0.0080535   -0.5327293
H     1.6775078    0.0140506    1.2929725
N     0.7409821    1.3003107   -0.3775096
H     0.7618152    1.3481629   -1.4119424
C     1.4444027    2.5247918    0.0914797
B    -0.7853577    1.3488754    0.0831269
N    -1.5038881   -0.0083324   -0.3488902
H    -1.3420661    2.2911983   -0.5052382
N     0.7518542   -1.2983208   -0.3631466
H    -0.8256859    1.4449269    1.3099766
B    -0.7738732   -1.3543153    0.0986795
C     1.4655923   -2.5114808    0.1194962
H     0.7730448   -1.3575749   -1.3969815
C    -2.9064363   -0.0113220    0.1470120
H    -1.5755520   -0.0142852   -1.3818599
H    -1.3229294   -2.3085054   -0.4775621
H    -0.8125636   -1.4356691    1.3267117
H     2.5153896   -2.5077983   -0.2435118
H     0.9432197   -3.4276471   -0.2296717
H     1.4678475   -2.4984286    1.2266745
H    -3.4387725    0.8980604   -0.2046614
H    -2.8862767   -0.0105713    1.2542435
H    -3.4345090   -0.9236801   -0.2033551
H     1.4486541    2.5230970    1.1987267
H     0.9130586    3.4325332   -0.2661037
H     2.4935830    2.5270371   -0.2733606
```

```
$vibrational spectrum
#  mode     symmetry     wave number   IR intensity    selection rules
#                         cm**(-1)        km/mol         IR     RAMAN
     1                       0.00         0.00000         -       -
     2                       0.00         0.00000         -       -
     3                       0.00         0.00000         -       -
     4                       0.00         0.00000         -       -
     5                       0.00         0.00000         -       -
     6                       0.00         0.00000         -       -
     7        a            121.73         0.89483        YES     YES
     8        a            126.79         0.86615        YES     YES
     9        a            144.08         5.17745        YES     YES
    10        a            227.75         0.04421        YES     YES
    11        a            227.91         0.30085        YES     YES
    12        a            237.50         0.01613        YES     YES
    13        a            239.21         3.90499        YES     YES
    14        a            240.69         4.04774        YES     YES
    15        a            337.27         8.26716        YES     YES
    16        a            338.57         8.48813        YES     YES
    17        a            360.66         8.00165        YES     YES
    18        a            390.31         0.00634        YES     YES
```

19	a	401.69	2.58775	YES	YES
20	a	403.23	2.50461	YES	YES
21	a	450.86	2.49364	YES	YES
22	a	742.18	0.53375	YES	YES
23	a	743.28	0.51380	YES	YES
24	a	745.50	0.24389	YES	YES
25	a	784.45	6.17174	YES	YES
26	a	785.07	6.08316	YES	YES
27	a	847.22	0.00337	YES	YES
28	a	893.24	36.27765	YES	YES
29	a	893.32	36.33801	YES	YES
30	a	926.84	0.00611	YES	YES
31	a	934.47	22.57963	YES	YES
32	a	995.15	6.84705	YES	YES
33	a	996.96	7.74345	YES	YES
34	a	1029.80	35.05061	YES	YES
35	a	1030.02	36.68929	YES	YES
36	a	1064.44	0.55668	YES	YES
37	a	1073.43	0.00498	YES	YES
38	a	1090.44	0.00542	YES	YES
39	a	1091.20	0.45127	YES	YES
40	a	1123.30	3.32082	YES	YES
41	a	1128.03	0.31981	YES	YES
42	a	1131.91	1.17461	YES	YES
43	a	1132.74	1.94427	YES	YES
44	a	1143.92	88.34244	YES	YES
45	a	1144.58	78.24827	YES	YES
46	a	1161.12	11.97156	YES	YES
47	a	1179.65	146.15336	YES	YES
48	a	1180.88	149.69742	YES	YES
49	a	1212.92	0.01256	YES	YES
50	a	1241.02	81.97999	YES	YES
51	a	1242.74	77.37158	YES	YES
52	a	1352.80	31.83244	YES	YES
53	a	1354.09	31.97365	YES	YES
54	a	1381.54	0.89100	YES	YES
55	a	1394.95	8.16367	YES	YES
56	a	1395.60	8.73886	YES	YES
57	a	1396.38	3.63190	YES	YES
58	a	1439.72	2.58437	YES	YES
59	a	1440.12	18.23973	YES	YES
60	a	1440.73	7.31552	YES	YES
61	a	1441.83	11.62351	YES	YES
62	a	1442.65	29.08332	YES	YES
63	a	1443.04	18.12614	YES	YES
64	a	2370.33	285.03455	YES	YES
65	a	2371.54	284.37111	YES	YES
66	a	2383.78	36.65160	YES	YES
67	a	2475.14	2.29599	YES	YES
68	a	2476.34	3.01374	YES	YES
69	a	2488.06	314.54965	YES	YES
70	a	2962.62	52.95413	YES	YES
71	a	2962.76	53.02114	YES	YES
72	a	2963.52	0.67389	YES	YES
73	a	3046.45	5.88717	YES	YES
74	a	3046.71	16.85173	YES	YES
75	a	3047.01	17.15041	YES	YES
76	a	3080.16	5.13392	YES	YES
77	a	3081.12	5.57712	YES	YES
78	a	3081.21	4.00936	YES	YES
79	a	3277.42	0.20939	YES	YES
80	a	3278.01	0.24829	YES	YES
81	a	3281.54	0.24451	YES	YES

$end

References

1 E. Framery and M. Vaultier, *Heteroat. Chem.,* 2000, **11**, 218–225.

2 A. Johnson, Antonio. J. Martinez-Martinez, Stuart. A. Macgregor and Andrew. S. Weller, *Dalton Trans.*, 2019, **48**, 9776.

3 A. J. Martinez-Martinez and A. S. Weller, Datlon Trans, 2019, **48**, 3551-3554

4 A. T. Lubben, J. Scott McIndoe and A. S. Weller, *Organometallics*, 2008, **27**, 3303–3306.

5 B. J. Cosier and A. M. Glazer, *J. Appl. Crystallogr.*, 1986, **19**, 105–107.

6 George M. Sheldrick, *Acta. Crystallogr.*, 2015, **71**, 3–8.

7 George M. Sheldrick, *Acta. Crystallogr.*, 2008, **64**, 112–122.

8 O. V. Dolomanov, L. J. Bourhis, R. J. Gildea, J. A. K. Howard and H. Puschmann, *J. Appl. Crystallogr.*, 2009, **42**, 339–341.

9 P. van der Sluis and A. L. Spek, *Acta. Crystallogr. A*, 1990, **46**, 194–201.

10 D. E. Ryan, K. A. Andrea, J. J. Race, T. M. Boyd, G. C. Lloyd-Jones and A. S. Weller, *ACS. Catal.*, 2020, **10**, 7443–7448.

11 J. R. Vance, A. Schäfer, A. P. M. Robertson, K. Lee, J. Turner, G. R. Whittell and I. Manners, *J. Am. Chem. Soc.*, 2014, **136**, 3048–3064.

12 Y. Yang, J. Zhang, Y. Wei, Q. Chen, Z. Cao, H. Li, J. Chen, J. Shi, Z. Xie and L. Zheng, *Nano. Res.*, 2018, **11**, 656–664.

13 G. A. Volpato, D. Muneton Arboleda, R. Brandiele, F. Carraro, G. B. Sartori, A. Cardelli, D. Badocco, P. Pastore, S. Agnoli, C. Durante, V. Amendola and A. Sartorel, *Nanoscale Adv.*, 2019, **1**, 4296–4300.

14 M. von Arnim and R. Ahlrichs, *J. Chem. Phys.*, 1999, **111**, 9183–9190.

15 O. Treutler and R. Ahlrichs, *J. Chem. Phys.*, 1995, **102**, 346–354.

16 K. Eichkorn, F. Weigend, O. Treutler and R. Ahlrichs, *Theoretica. Chimica. Acta.*, 1997, **97**, 119–124.

17 K. Eichkorn, O. Treutler, H. Öhm, M. Häser and R. Ahlrichs, *Chem. Phys. Lett.*, 1995, **242**, 652–660.

18 P. Deglmann, K. May, F. Furche and R. Ahlrichs, *Chem. Phys. Lett.*, 2004, **384**, 103–107.

19 P. Deglmann, F. Furche and R. Ahlrichs, *Chem. Phys. Lett.*, 2002, **362**, 511–518.

20 R. Ahlrichs, M. Bär, M. Häser, H. Horn and C. Kölmel, *Chem. Phys Lett.*, 1989, **162**, 165–169.

21 P. Császár and P. Pulay, *J. Mol. Struct.*, 1984, **114**, 31–34.

22 S. Grimme, J. Antony, S. Ehrlich and H. Krieg, *J. Chem. Phys.*, 2010, **132**, 154104.

23 S. Grimme, S. Ehrlich and L. Goerigk, *J. Comput. Chem.*, 2011, **32**, 1456–1465.

24 A. Klamt and G. Schüürmann, *J. Chem. Soc., Perkin Trans. 2*, 1993, 799–805.
